# Innovative Photon‐Engineered Fluorescent Tri‐Layer Polymeric Coatings for Sub‐Ambient Colored Radiative Cooling

**DOI:** 10.1002/advs.202511599

**Published:** 2025-09-03

**Authors:** Tao Wang, Ying Liu, Qingdong Xuan, Xue Ma, Yanbo Fang, You Dong, Dangyuan Lei, Jian‐Guo Dai

**Affiliations:** ^1^ Department of Civil and Environmental Engineering The Hong Kong Polytechnic University Kowloon Hong Kong 999077 China; ^2^ Department of Materials Science and Engineering City University of Hong Kong Hong Kong 999077 China; ^3^ Department of Refrigeration and Cryogenics Engineering Hefei University of Technology 193 Tunxi Road Hefei 230009 China; ^4^ Department of Physics Center for Functional Photonics, Hong Kong Branch of National Precious Metals Material Engineering Research Centreand Hong Kong Institute of Clean Energy City University of Hong Kong Hong Kong 999077 China; ^5^ Department of Architecture and Civil Engineering City University of Hong Kong Kowloon Hong Kong 999077 China

**Keywords:** colored radiative cooling, effective solar reflectance, fluorescent cooling, self‐cleaning coating

## Abstract

Colored radiative cooling (CRC) materials provide a sustainable solution to thermal management, mitigating global warming while maintaining aesthetic appeal. Nevertheless, conventional CRC materials exhibit reduced cooling efficiency due to their significant sunlight absorption and degraded optical performance in dusty outdoor environments. Developing self‐cleaning CRC materials with high cooling performance and vibrant color remains challenging. Here, photon‐engineered fluorescent tri‐layer polymeric coatings (PFTPCs) is presented, which have an effective reflectance >100% at fluorescent emission wavelengths, yielding a historically high colored cooling capacity. By leveraging Purcell‐enhanced fluorescent emission along with optimized photonic structures, the PFTPCs exhibit an effective solar reflectance of 94%, 96.3%, and 96.1% for red, yellow, and green colors, respectively. These coatings also demonstrate long‐wave infrared emissivity surpassing 96%. Consequently, the PFTPCs achieve daytime sub‐ambient cooling of 5.4–7.2 °C, outperforming commercial colored counterparts by 3.7–5.1 °C. Simulations indicate that when applied as roof and wall coatings, PFTPCs can significantly contribute to building energy savings across diverse climate zones. PFTPCs also exhibit excellent superhydrophobic properties, anti‐fouling capability, and durability, providing strong resistance to ultraviolet irradiation, mechanical abrasion, rain, and soiling. This research paves the way for the rational design of high‐performance fluorescence‐assisted colored radiative cooling materials, promoting energy conservation and sustainability.

## Graphical abstract

This work provides a photoluminescence‐enhanced coating material solution for enhancing solar reflectance and coloration, systematically investigating the fluorescence’s role on the optical performance. All colored coatings exhibit excellent sub‐ambient cooling capacity (historically high solar reflectance and over 0.96 infrared emittance), while preserving vivid color and self‐cleaning performance for aesthetic and real‐world application requirements.

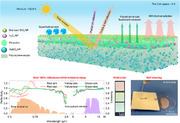

## 1 Introduction

The global demand for cooling solutions has surged due to climate change‐induced global warming. Excessive air conditioning usage exacerbates greenhouse gas emissions, thereby intensifying the greenhouse effect. Passive daytime radiative cooling (PDRC) operates by transferring heat to the cold outer space through passive thermal radiation. Conventional PDRC materials typically exhibit a white or mirror‐like appearance due to their high reflectivity across the solar wavelength range (0.25–2.5 µm) and high long‐wave infrared (LWIR) emittance within the atmospheric window (8–13 µm). With the increasing commercialization of PDRC products such as paints,^[^ ^1^, ^2^ ^]^ textiles,^[^ ^3^, ^4^, ^5^ ^]^ and ceramics,^[^ ^6^, ^7^ ^]^ color has become a primary consideration not only for aesthetics but also for functional applications such as visual camouflage with radiative cooling^[^ ^8^, ^9^ ^]^ and colored cool roads with retroreflective properties.^[^ ^10^, ^11^ ^]^

One effective strategy for achieving color while maintaining high radiative cooling performance involves the use of well‐designed photonic structures. These include multilayer photonic crystals,^[^ ^12^, ^13^, ^14^ ^]^ localized surface plasmon resonant structures,^[^ ^15^, ^16^ ^]^ and periodic iridescent micro/nano structures.^[^ ^17^, ^18^, ^19^ ^]^ However, sophisticated structure configurations often require equipment‐intensive manufacturing processes such as deposition and lithography, which are costly and undesirable for large‐scale fabrication. On the other hand, several colorant materials such as commercial solar‐absorptive dyes^[^ ^20^, ^21^, ^22^ ^]^ and fluorescent pigment materials,^[^ ^23^, ^24^, ^25^, ^26^ ^]^ can be facilely hybridized into scalable particle‐embedded coating structures. In these systems, a “thin colored top with a solar‐reflective white bottom” bilayer design is typically employed for colored radiative cooling (CRC) design. In contrast to commercial solar‐absorptive dyes, fluorescent materials can convert the absorbed light into fluorescent photons and re‐emit them at longer wavelengths, thereby reducing solar heating during coloration. Several photoluminescent properties, including fluorescent spectra, photoluminescence quantum yield (PLQY), and Stokes shift, have been crucial to the design of fluorescence‐assisted CRC coatings.^[^ ^27^ ^]^ PLQY is the number ratio of re‐emitted fluorescent photons to excitation photons. Stokes shift is defined as the wavelength difference between the maximum of the absorption and emission spectra. A small Stokes shift of fluorescent pigment is preferable for enhancing the efficiency of fluorescence in reducing solar heat,^[^ ^28^ ^]^ while low PLQY significantly weakens fluorescence's ability in enhancing solar reflectance and coloration. Compared to other fluorescent perovskite quantum dot materials^[^ ^24^, ^25^ ^]^ and organic fluorescent molecules,^[^ ^29^ ^]^ rare‐earth ions doped inorganic phosphors are nontoxic and suitable for colored fluorescence‐assisted cooling due to their moderate PLQY, large color rendition index, high resistance to degradation in the polymer matrix, and cost‐effectiveness.^[^ ^23^ ^]^

Although fluorescence provides a promising solution for high‐performance CRC material development, conventional UV–visible‐NIR spectrometry cannot accurately measure the effective solar reflectance of fluorescent coatings due to their intrinsic photon wavelength conversion,^[^ ^30^, ^31^ ^]^ hindering the optical performance optimization of fluorescent CRC coatings. Additionally, the self‐cleaning capacity and durability of colored radiative cooling materials have garnered considerable attention due to their long‐term optical performance requirements in real‐world applications. The self‐cleaning capacity can be achieved through creating a superhydrophobic surface, which necessitates hierarchical surface roughness and low surface‐free‐energy chemical properties.^[^ ^32^ ^]^ However, the micro/nano surface structure can intensify the visible light scattering to some extent,^[^ ^33^, ^34^ ^]^ whitening and compromising the colorful appearance of CRC coatings. Consequently, developing CRC coatings that simultaneously achieve high sub‐ambient cooling performance, vibrant coloration, and effective self‐cleaning functionality remains a grand challenge.

In this study, we present an innovative type of photon‐engineered fluorescent tri‐layer polymeric coating with high sub‐ambient colored radiative cooling performance. This tri‐layer coating structure employs poly‐styrene‐acrylic as the matrix to hybridize functional fillers, comprising a solar‐reflective bottom BaSO_4_ layer, a colored middle phosphor/Y_2_O_3_ layer, and a solar‐transparent uppermost silane‐modified SiO_2_ layer. Purcell enhancement was leveraged to facilitate the fluorescent emission of three phosphors: Sr_2_Si_5_N_8_:Eu^2+^ (for red), Y_3_Al_5_O_12_:Ce^3+^ (for yellow), and Lu_3_Al_5_O_12_:Ce^3+^ (for green). We also developed a modified Monte Carlo (MMC) method to simulate and optimize the spectral properties of the photon‐engineered fluorescent tri‐layer polymeric coatings (PFTPCs). Notably, the photon‐engineered PFTPCs could show historically high effective solar reflectance (ESR) of 94%, 96.3%, and 96.1% for the red, yellow, and green colors, respectively, as well as excellent infrared emittance of over 96% in the atmospheric window (8–13 µm). As a result, the PFTPCs demonstrate sub‐ambient cooling temperatures of 5.4–7.2 °C. The PFTPCs also outperform their commercial counterparts, achieving 3.7 to 5.1 °C cooler temperatures under high solar irradiation of ≈850 W m^−2^. Further, building modeling confirmed that the PFTPCs possess great energy‐saving potential in roof and wall coating applications. Additionally, the PFTPCs exhibit excellent self‐cleaning abilities, including a good anti‐fouling property and high resistance to solid dust and typical soiling in diverse climate zones. The PFTPCs also show good durability, with high ultraviolet (UV) aging resistance, good abrasion resistance, and high tolerance to high‐speed water impact. Our innovations offer a sustainable way for developing high‐performance fluorescence‐assisted CRC coatings while retaining competitive commercialization and application potential.

## 2 Results and Discussion

### 2.1 Design Rationale

**Figure 1:**
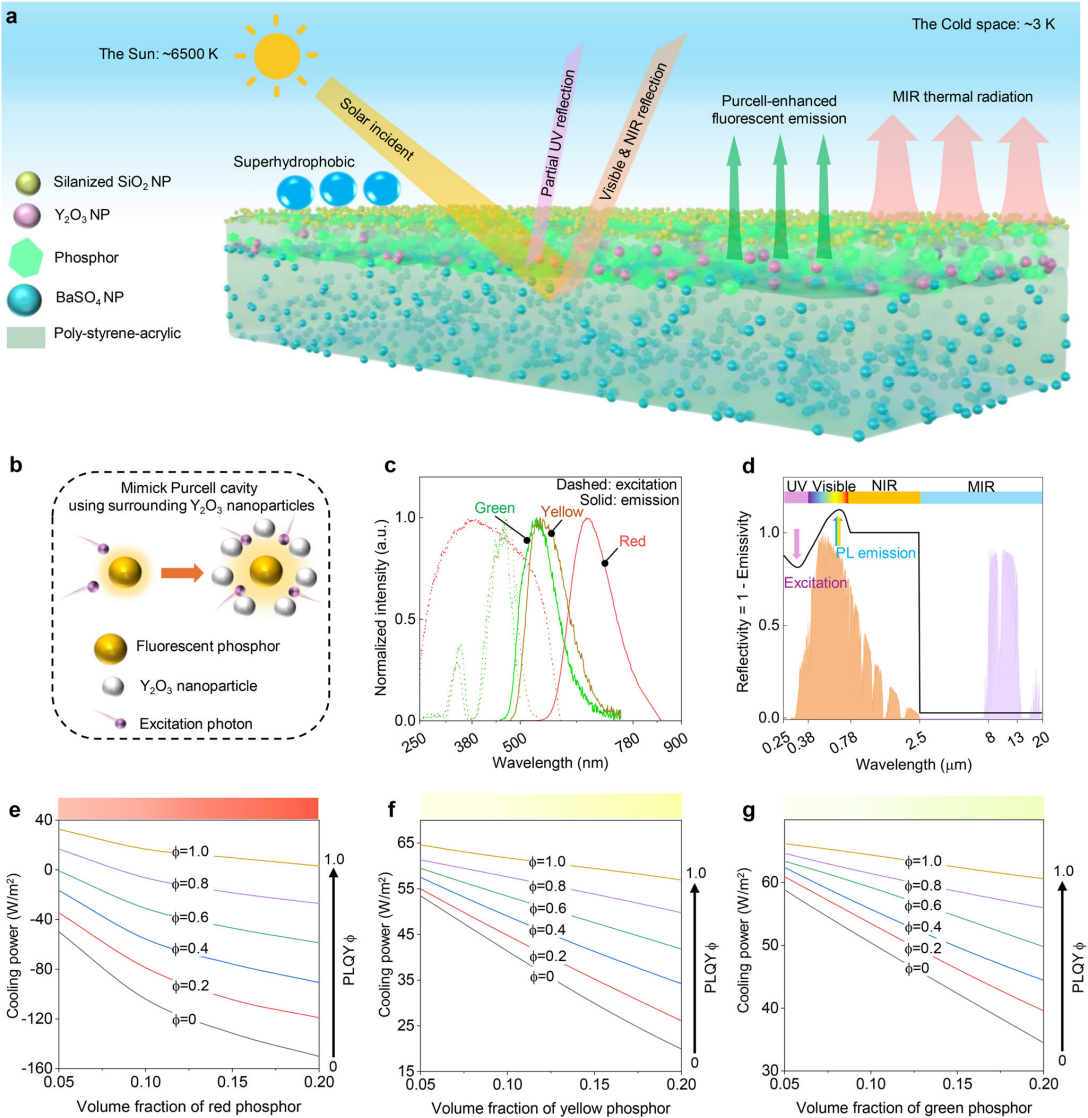
Design and modeling of the photon‐engineered fluorescent tri‐layer polymeric coatings (PFTPCs). a) Structure and working mechanism of the PFTPCs. b) Schematic for mimicking the Purcell cavity by utilizing Y_2_O_3_ nanoparticles that surround the phosphor. c) Excitation and emission spectra of three phosphors: Sr_2_Si_5_N_8_:Eu^2+^ for red, Y_3_Al_5_O_12_:Ce^3+^ for yellow, and Lu_3_Al_5_O_12_:Ce^3+^ for green (Background shade: Normalized AM 1.5 solar irradiance). d) Schematic for ideal reflectivity spectra of proposed fluorescent colored radiative cooling coating (Yellow shade: Normalized solar irradiance AM 1.5. Blue shade: atmospheric window). Cooling power of the e) red, f) yellow, and g) green fluorescent coatings with increasing volume fraction of phosphors and PLQYs, where the volume fraction of Y_2_O_3_ and the colored middle layer thickness for the three colored coating structures were 0% and 20 µm, respectively. The color bar on the upper side of the figure refers to the color variation with increasing phosphor content.

The photon‐engineered fluorescent coating structure is illustrated in **Figure** **1**a towards high optical performance, vivid colorful appearance, and self‐cleaning capacity. This tri‐layer coating architecture utilizes a poly‐styrene‐acrylic matrix for hybridization with different functional fillers, integrating a solar‐reflective bottom layer of BaSO_4_ nanoparticles (NPs), a colored middle layer of phosphor/Y_2_O_3_ NPs, and a solar‐transparent topmost layer of silane‐modified SiO_2_ NPs. Different from several reported colored bilayer coatings incorporating TiO_2_‐based bottom and colored top layer,^[^ ^21^, ^23^, ^35^ ^]^ an ultra‐white bottom layer was used to reflect visible and near‐infrared (NIR) light that passes through the colored middle layer, maximizing solar reflectance and providing a high infrared emissivity. BaSO_4_ NPs with the mean size of 1.5 µm were used in the bottom layer to facilitate multiple Mie scattering for solar light and high infrared emissivity by optimizing the particle size (Figures S1–S3, Supporting Information) for efficient solar‐scattering efficiency and effective electromagnetic absorption within the atmospheric window through intrinsic phonon resonance^[^ ^36^ ^]^ (Figure S4, Supporting Information). The colored middle layer serves the dual purpose of coloration and reducing heat load via efficient fluorescence re‐emission. Purcell enhancement was exploited to enhance fluorescence re‐emission and PLQY (Figure 1b). Y_2_O_3_ NPs with appropriate particle size distribution (mean size: 1 µm, Figure S3b, Supporting Information) were employed in the colored layer to optimize the surrounding dielectric environment of three phosphors by providing scattering efficiencies that cover peak emission wavelengths of the phosphors (Figures S5 and S6, Supporting Information). Then, harnessing the rough surface morphology of the colored middle layer and its low surface‐free‐energy properties, an ultrathin transparent uppermost layer can be positioned atop the middle layer for constructing a hierarchical micro/nano surface structure, which enables the superhydrophobic self‐cleaning ability of the PFTPC for long‐term optical properties. This uppermost layer design does not affect the pristine color properties, since the utilized silanized SiO_2_ NPs exhibit negligible light scattering within the solar wavelength range due to their low refractive index (≈1.56) and 20‐nm sizes. Figure 1c displays the excitation and emission spectra of the utilized red, yellow, and green phosphors, which indicates that these three phosphors exhibit inherent UV and visible light absorption while mainly emitting fluorescence in the visible light range. The excitation and emission profiles of these three phosphors show an ≈70 nm spectral overlap, spanning from the emission onset to the excitation cutoff. Such overlap could potentially reduce fluorescence conversion efficiency, since emitted photons within this shared wavelength range may undergo reabsorption by the phosphor material, resulting in additional heat generation during the fluorescence transformation. Nevertheless, given the unique spectral position of this overlapping region, both excitation and emission photon distribution densities remain relatively weak, suggesting that fluorescent photon reabsorption occurs at a minimal frequency and the chosen phosphors remain well‐suited for designing high‐performance CRC coatings. Additionally, the red phosphor has a much larger Stokes shift compared to the yellow and green phosphors. Then, the schematic for ideal spectra of this tri‐layer coating structure, depicted in Figure 1d, illustrates partial absorption of UV and visible light, accompanied by re‐emitted fluorescence in emission wavelength ranges. High NIR reflection is essential to prevent excessive solar heating. The ESR across solar wavelengths is rationally expressed as:^[^ ^26^ ^]^

(1) $$\mathrm{ESR}\left(\lambda \right)=\frac{f\_b\left(\lambda \right)}{f\_i\left(\lambda \right)}=\frac{f\_bf\left(\lambda \right)+f\_bsol\left(\lambda \right)}{I_{\mathrm{solar}}\left(\lambda \right)}$$

where f_i(λ) refers to the spectral incident solar flux, and *I*_solar_(*λ*) is the ASTM G173‐03 Global solar intensity spectrum, f_b(λ) denotes the backward energy flux within the emission wavelength range, incorporating the backward fluorescence flux (f_bf(λ)) and the reflected solar flux (f_bsol(λ)) at a given wavelength *λ*. For non‐emitted wavelengths, f_bf(λ)=0. As the backward fluorescence contributes to the backward energy flux, there exists a possibility that spectral reflectivity within the photoluminescence (PL) emission wavelength range can surpass 100% according to Equation (1).

In the mid‐infrared (MIR) wavelength range, broadband emissivity is favored to mitigate the over‐cooling effect during nighttime, thereby alleviating diurnal temperature variation‐induced structural deformation of buildings.^[^ ^37^ ^]^ Through heat transfer analysis (Figure S7, Supporting Information), broadband emissivity is beneficial for achieving a higher cooling power in real‐world applications (less than 3 °C, Figure S7b, Supporting Information). Figure S8 (Supporting Information) illustrates the relationship between cooling power and solar reflectance under diverse relative humidity (RH) conditions. A solar reflectance above 95% is optimal for achieving a sub‐ambient cooling effect in highly humid climate regions with over 50% RH,^[^ ^2^ ^]^ such as subtropical/tropical Hong Kong and Singapore that typically experience higher year‐round humidity and solar irradiation compared to mid‐altitude and sub‐arctic climate regions. Therefore, a high ESR over 0.95 is essential for colored fluorescent coatings to exhibit sub‐ambient cooling in highly humid regions.

### 2.2 Modeling and Optimization of PFTPCs

Based on the design rationales of the PFTPCs, we focused on optimizing the white bottom layer and colored middle layer. By optimizing the filler's volumetric content and the thickness of the bottom layer, the optimal volume fraction of BaSO_4_ NPs and the bottom layer thickness were determined to be ≈75% and ≈500 µm, respectively (Figure S9, Supporting Information). This configuration enables both optimal solar reflectance and LWIR emittance of the bottom coating. To estimate the effective spectral properties of the PFTPCs and eliminate resource‐intensive trial‐and‐error experimental screening in cooling capacity optimization, a MMC method was developed to simulate the spectral response of coating structures with fluorescent pigments (Figures S10–S12, Supporting Information). Its accuracy and correctness were further confirmed by comparing simulation results with experimental ESRs (Figure S13, Supporting Information), demonstrating its critical role as a generic method for optical simulation of colored radiative cooling coating materials with or without fluorescent pigments.

Regarding the colored middle layer, we investigated the effects of several key factors, including volume fractions of Y_2_O_3_ NPs and phosphors, as well as PLQY, on cooling performance and color appearance of the PFTPCs with the optimal white bottom through the MMC method. Given the phosphor's size distribution (Figure S14, Supporting Information), the colored layer thickness was set to 20 µm since a thin colored layer is preferred to reduce solar light absorption, meanwhile minimizing material costs. We modeled this tri‐layer coating structure and conducted a simulation for the effects of increasing volume fractions of Y_2_O_3_ NPs and phosphor, since the light scattering of Y_2_O_3_ NPs interacts with the excitation light absorption and fluorescence re‐emission processes of phosphors. As shown in Figures S15–S17 (Supporting Information), more Y_2_O_3_ NPs usually lead to more intensive solar light scattering and higher ESR. However, an exception was observed for the red phosphor. As shown in Figure S15 (Supporting Information), the ESR with 10 vol.% Y_2_O_3_ NPs is lower than without Y_2_O_3_ NPs. This can be attributed to the Mie scattering offered by adding a small amount of Y_2_O_3_ NPs. The strong absorption ability of the red phosphor makes the absorption of excitation photons easier than the yellow and green phosphors, leading to a decline in ESR. When the number of scattering centers (Y_2_O_3_ NPs) continues to increase, the scattering ability of Y_2_O_3_ NPs dominates in the coating composite, causing the ESR to rise again.

We also explored the effects of increasing the volume fraction of phosphors and PLQY, as more phosphors enhance color saturation, while higher PLQYs are crucial for mitigating heat generation during the photoluminescent process (Figures S18–S20, Supporting Information). As shown in Figure S18 (Supporting Information), the effect of PLQY on the red phosphor is more pronounced than on the yellow and green phosphors (Figures S19 and S20, Supporting Information), which can be attributed to the relatively higher wideband excitation light absorption properties of the red phosphor. Therefore, PLQY is significantly more impactful for phosphors with higher excitation light absorption properties. Notably, the distribution of PLQYs over the excitation wavelength range influences the heat load generation during the excitation light‐fluorescence conversion process. For simplicity and without losing generality, a uniform distribution of PLQYs was utilized in the aforementioned simulation.

Importantly, CRC coatings exhibit varying cooling capacities under skies with differing atmospheric transparency conditions. An ESR exceeding 0.95 is almost essential to achieve sub‐ambient cooling effects under tropical and subtropical regions such as Hong Kong and Singapore, where RH and ambient temperature are elevated (Figure S8, Supporting Information). Assuming that near‐unity PLQY could be attained through Purcell enhancement, and based on MMC simulation results (Figures S15–S20, Supporting Information), to achieve ESR exceeding 0.95 and produce conspicuous colors, the volume fractions of Y_2_O_3_ NPs and phosphors for the red fluorescent coating were selected as 60 vol.% and 5 vol.% respectively, for the outdoor cooling test. In contrast, the volume fractions of those two fillers for yellow and green coatings were both selected as 40 vol.% and 10 vol.%, respectively.

Additionally, to elucidate the impacts of PLQY of three phosphors on cooling capacity, simulations were performed on a coating structure with varied phosphor content and devoid of Y_2_O_3_ NPs within a 20‐µm‐thick colored middle layer, in the absence of Purcell enhancement. The results, illustrated in Figure 1e–g, reveal that PLQY plays a more pivotal role in cooling power as the number of phosphors increases. Notably, for highly excitation light‐absorptive phosphors, such as the red phosphor used in this study, PLQY predominantly influences cooling power. In terms of 20 vol.% phosphor concentration, increasing PLQY from 0% to 100% results in an approximate enhancement of cooling power by 180 W m^−2^ for the red fluorescent coating. In contrast, the yellow and green fluorescent coatings exhibit enhancements of ≈40 W m^−2^ and ≈30 W m^−2^, respectively. Therefore, leveraging Purcell enhancement to enhance fluorescent emission is imperative to mitigating solar heating during coloration.

### 2.3 PFTPCs Characterization

**Figure 2:**
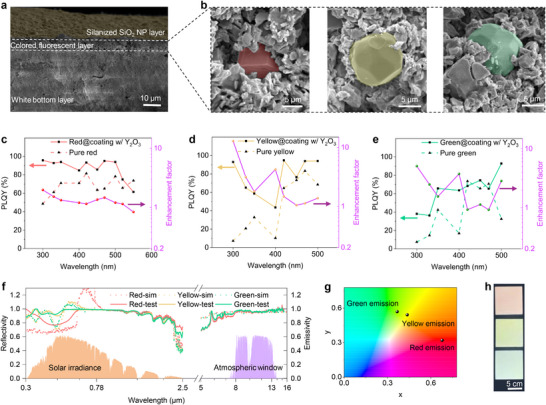
Structural, spectral, and color characterizations of the photon‐engineered fluorescent tri‐layer polymeric coatings (PFTPCs). a) Cross‐sectional morphology of the fluorescent tri‐layer polymeric coating structure. The uppermost silanized SiO_2_ NP layer is shown with a yellow false color. Scale bar, 10 µm. b). Morphologies of three phosphors embedded in the coating surrounded by Y_2_O_3_ nanoparticles. The phosphor is highlighted by the corresponding false color. From left to right: red, yellow, and green. Scale bar, 5 µm. PLQYs of the c) red phosphor, d) yellow phosphor, and e) green phosphor embedded in coating with and without Y_2_O_3_ NPs. f) Solar reflectivity and infrared emissivity spectra of the PFTPCs obtained by the conventional UV–visible‐NIR spectrometric test and the MMC simulation. g) The chromaticity of the fluorescent emission of three phosphors in the 1931 CIE color space. h) Optical images of the PFTPCs under sunshine. Samples from top to bottom are red, yellow, and green, respectively. Scale bar, 5 cm.

As shown in Figure S21 (Supporting Information), the optimal white bottom layer coating exhibited a solar reflectance of 97.6% with a high LWIR emittance of 94.0% and broadband emittance (2.5–15.4 µm) of 95.4%. The experimental optical properties of the white bottom coating closely align with the simulated result, which indicates a solar reflectance of 96.1% and an LWIR emittance of 95.1%. The marginally higher solar reflectance observed in the experimental results can be attributed to the broad size distribution of BaSO_4_ NPs and the presence of a few air pores in the bottom layer (Figure S22, Supporting Information). Additionally, the bottom layer can be applied on various substrates, including concrete, wood, steel, and plastic (Figure S23, Supporting Information). The ≈20‐µm‐thick colored middle layer was positioned atop the white base shown in **Figures** **2**a and S24 (Supporting Information). The cluster of phosphors surrounded by appropriately sized Y_2_O_3_ NPs (Figure 2b), forms the Purcell cavity, which accelerates fluorescence re‐emission and enhances PLQYs. Figure 2c–e shows the PLQYs across the excitation wavelength range, demonstrating good enhancement by the Purcell cavity. An enhancement factor of PLQY was rationally defined as:

(2) $$\begin{array}{ccc} & & Enhancement\;factor\left(\lambda_{\mathrm{ex},i}\right)\\ & & \;=\frac{\mathrm{PLQY}\;\mathrm{of}\;\mathrm{the}\;\mathrm{phosphor}\;\mathrm{in}\;\mathrm{coating}\;\left(\lambda_{\mathrm{ex},i}\right)}{\mathrm{PLQY}\;\mathrm{of}\;\mathrm{the}\;\mathrm{pure}\;\mathrm{phosphor}\;\left(\lambda_{\mathrm{ex},i}\right)} \end{array}$$

Interestingly, the evolving trend of enhancement factors of the phosphor along with excitation wavelength completely converses with that of PLQYS of the pure phosphor. The reason is straightforward: the pristine lower PLQYs of these phosphors at several excitation wavelengths indicate considerable non‐radiative decay, which can be efficiently suppressed by the Purcell effect. In other words, the high pristine PLQYs limit the Purcell effect, thereby leading to relatively low enhancement factors. Additionally, the Purcell effect efficiently improves the PLQY in the UV range, for instance, the enhancement factor of the yellow phosphor at 300 nm is over 12, which suggests more UV photons are converted into fluorescence, which also alleviates the UV aging of the organic polymer coating matrix. The obvious improvement of PLQY in the UV range can be ascribed to the high scattering efficiencies supported by the surrounding Y_2_O_3_ NPs (Figure S6, Supporting Information).

At peak excitation wavelengths, PLQYs increased by ≈40% for the red phosphor (≈350 nm) and by 5% and 10% for the yellow and green phosphors (≈450 nm), respectively. The relatively low PLQY enhancement percentage for the yellow and green phosphors can be attributed to imperfect matching between the scattering efficiency peak wavelength of the 1‐µm Y_2_O_3_ NP and the PL emission peak wavelengths of these two phosphors. Additionally, the acceleration extent of PL lifetime decay is also consistent with the PLQY enhancement difference among these three phosphors (Figure S25 and Tables S1–S3, Supporting Information). The acceleration of PL lifetime decay can be ascribed to the dielectric environment modification induced by Y_2_O_3_ NPs surrounding the phosphors. To further enhance long‐term performance, particularly self‐cleaning properties for maintaining optical properties, a thin (≈3 µm) transparent uppermost layer was added to the as‐fabricated bilayer structure. This silanized SiO_2_ NP layer had a negligible effect on the solar reflectivity of the middle‐bottom bilayer structure (Figure S26, Supporting Information), but increased LWIR emittance by ≈3% (Figure S27 and Table S4, Supporting Information) due to the intensive inherent infrared‐lossy properties of the silane‐modified SiO_2_ NPs within the atmospheric window (Figure S28, Supporting Information).

Finally, the solar reflectivity and infrared emissivity spectra of fluorescent tri‐layer polymeric cooling coatings are presented in Figure 2f. The simulated solar reflectance, determined by PLQYs of phosphors embedded with Y_2_O_3_ NPs in the coating matrix, was 93.0%, 95.8%, and 95.7% for red, yellow, and green coatings, respectively. Some simulated reflectivity data points within the emission wavelength range surpass 100%, indicating an effective contribution of fluorescence to ESR and coloration. For example, the red fluorescent coating achieves an ESR over 120% at its emission peak wavelength. It is interesting to note a discrepancy in ESR and the measured solar reflectivity within excitation wavelengths, which can be ascribed to the working mechanism of commercial spectrometers. The conventional UV–Vis‐NIR spectrophotometer utilizes a photodetector to quantify reflectance by illuminating the sample with monochromatic radiation at a given wavelength. Although suitable for non‐fluorescent materials, this approach fails to accurately analyze fluorescent coatings. When excited by incident light, the emission from a fluorescent surface contains both unconverted excitation light and generated fluorescence. The instrument measures the combined signal at the excitation wavelength without distinguishing the fluorescence contribution, leading to pseudo reflectance spectra for fluorescent coating materials. Moreover, the simulated effective solar reflectance can be validated by a fitting method, as detailed in the Supporting Information. In contrast with the non‐fluorescent counterparts, the solar reflectance of non‐fluorescent colored coatings was 87.6%, 94.0%, and 94.5% for the red, yellow, and green colors, respectively, obtained by setting PLQY to be zero. Therefore, the fluorescence's contributions to effective solar reflectance are 5.4%, 1.8%, and 1.2% for the red, yellow, and green colors, respectively. Additionally, all the PFTPCs exhibited LWIR emittance exceeding 96%, facilitating efficient radiation heat transfer to outer space.

Based on the PL emission spectra, we calculated the chromaticity, which is shown in Figure 2g. Figure 2h shows the appearance of the PFTPCs under sunlight. It is evident that fluorescence effectively contributes to coloration, as PLQY is enhanced by the Purcell cavity when exposed to sunlight. For instance, the green fluorescent coating exhibited a light cyan color, which could be attributed to the strong fluorescent emission in the 490‐520 nm range of the green phosphor (Figure S5c, Supporting Information).

### 2.4 Radiative Cooling and Energy‐saving Performance

**Figure 3:**
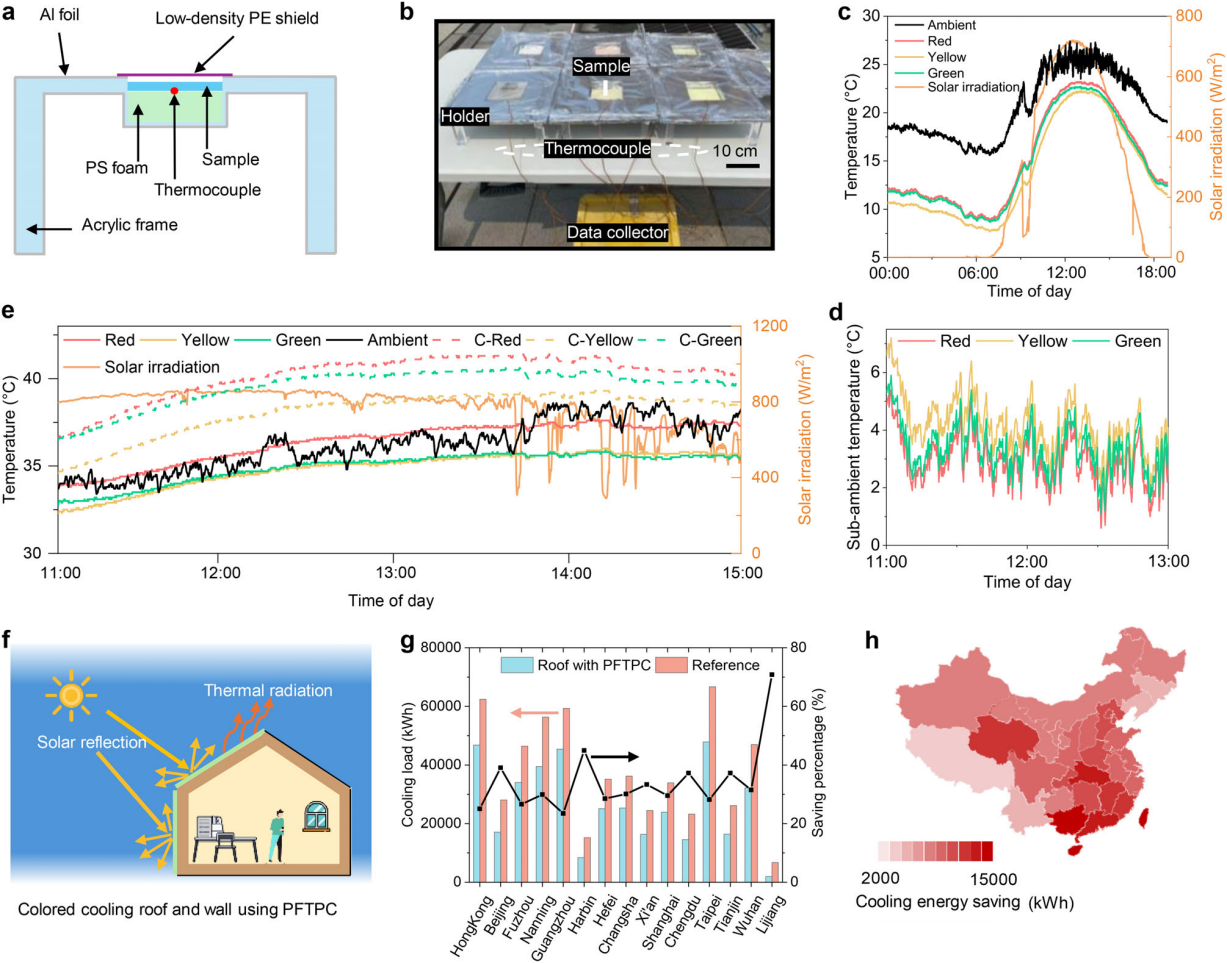
Outdoor cooling measurements and energy‐saving potential. a) Schematic of test holder configuration. b) Photograph of field test setup. c) Temperature of the PFTPCs on a sunny day with moderate relative humidity of 32% (6 Jan 2025). The labels “Red”, “Yellow”, and “Green” indicate fluorescent coatings. d) Temperature drops of the PFTPCs compared to ambient on 6 Jan 2025. e) Temperature of coating samples on a sunny day with a high relative humidity of 57% (13 April 2024). The prefix “C” refers to the commercial coating counterpart. f) Schematic of the PFTPCs applied as colored cooling roof or wall coating. g) Cooling load and energy saving percentages of the PFTPCs as a roof coating in primary cities in China. h) Energy saving of the PFTPCs as a roof coating mapping to different provinces in China.

The outdoor cooling assessment was conducted in Hong Kong, which is representative of a subtropical climate featured by high humidity and ambient temperature. The test holder and setup are shown in **Figure** **3**a,b, respectively. Outdoor tests for the PFTPCs were carried out over three days with varying weather conditions: sunny with moderate RH, sunny with high RH, and cloudy. Figures 3c,d present that all fluorescent coatings exhibited a sub‐ambient cooling effect. During the noontime of 11:00–13:00, the red fluorescent coating achieved a sub‐ambient temperature reduction up to 5.4 °C, while the yellow and green fluorescent coatings reduced the temperature by up to 7.2 and 5.9 °C below the ambient, under a solar intensity of ≈700 W m^−2^ and a moderate RH of ≈32% (Figure S29, Supporting Information). Additionally, the average sub‐ambient temperature drops were 2.9, 4.2, and 3.4 °C for red, yellow, and green colors, respectively (Figure 3d).

To demonstrate fluorescence's contribution to cooling and obtain reference data for fitting the ESR, commercial acrylic‐based coating counterparts of the comparable colors were fabricated into a bilayer configuration with identical bottom layers and compared with the PFTPCs (Figure S30, Supporting Information). Real‐time temperatures of all coating samples on a sunny day with high RH are shown in Figure 3e. During the noontime of 12:00–13:30, the red fluorescent coating exhibited a nearly ambient temperature, whereas both the yellow and green fluorescent coatings displayed sub‐ambient cooling of up to 2.1 and 1.9 °C, respectively under conditions of a solar irradiation power of ≈850 W m^−2^ and high RH of ≈57% (Figure S31, Supporting Information). Remarkably, the fluorescent coatings demonstrated significantly lower temperatures compared to their commercial counterparts, with a reduction of up to 4.3, 3.7, and 5.1 °C for red, yellow, and green colors, respectively. The fitted ESRs of the fluorescent red, yellow, and green coatings were 94.0%, 96.3%, and 96.1%, respectively, as shown in Figure S32 (Supporting Information). The simulated ESR values based on PLQYs of phosphors embedded in the matrix with Y_2_O_3_ NPs (93.0%, 95.8% and 95.7% for red, yellow and green, respectively) are in excellent agreement with our experimental results when considering a slight underestimated simulated value of the bottom layer's solar reflectance, providing unambiguous evidence that confirms the validity and accuracy of our MMC method, as well as the role of Purcell enhancement. Figure S33 (Supporting Information) shows that the average temperatures of fluorescent yellow and green coatings were slightly higher than the white bottom coating by ≈0.3 °C at noon time, indicating the high effective solar reflectance of these two coatings.

To assess the cooling capacity under varied yet usual climatic conditions, a field test was also conducted on a subsequent cloudy day (Figures S34, Supporting Information). Figure S34a (Supporting Information) shows temperature reductions of colored fluorescent coatings on this overcast day. The cooling effect is evidently diminished due to higher humidity (≈63%) (Figure S31, Supporting Information). Nevertheless, the red fluorescent coating maintained ambient‐level temperatures, while the yellow and green fluorescent coatings achieved sub‐ambient cooling temperatures of up to ≈3 °C, even on this heavily overcast day, indicating the high radiative cooling performance of PFTPCs. Simultaneously, the fluorescent coatings demonstrated noticeably lower temperatures compared to their commercial counterparts, with reductions of up to 3.6, 3.2, and 4.6 °C for red, yellow, and green colors, respectively. To further validate the accuracy of our fitted ESRs, we calculated the modeling temperatures of PFTPCs based on the heat transfer model (Figure S7, Supporting Information), and meteorological data during 13‐14 April 2024 (Figure S31, Supporting Information), which shows a good agreement with real‐time recorded temperatures (Figure S35, Supporting Information).

Further building energy simulations across China were conducted to evaluate the annual cooling energy‐saving performance of the PFTPCs in various climate zones (Figure S35 and Tables S5–S7, Supporting Information). Our PFTPCs exhibit significant application potential in colored cooling roof and wall coatings (Figure 3f). The roof PFTPC yielded an exceptional energy‐saving performance across almost all different climates (Figure 3g,h), achieving energy saving of up to 18 808 kWh (≈67.7 GJ with nearly a 30% saving percentage, Taipei), underscoring the potential significance of fluorescence in colored cooling roof coatings. To further illustrate the importance of PFTPC design, we performed simulations of the PFTPC as a colored cooling wall coating. The building model with the PFTPC applied on walls achieved a high energy‐saving performance across the whole China (Figures S36 and S37, Supporting Information), with an energy saving of up to 5786 kWh (≈20.8 GJ with a ≈10% saving percentage, Hong Kong), demonstrating its applicability and substantial energy‐saving potential in wall coating application. However, in regions where buildings experience both cold winters and hot summers, the application of the PFTPC requires careful evaluation. In colder seasons, excessive thermal emission might reduce heat conservation, whereas high solar reflectance may hinder natural solar warming. This limitation could compromise the year‐round energy‐saving capacity of the PFTPC, which is a common issue of radiative cooling coating applications under these mixed climates.^[^ ^38^ ^]^ Addressing this balance, a possible approach is to create radiative cooling materials with adjustable seasonal performance, like temporarily disabling radiative cooling and solar‐reflective features during winter. Such systems could incorporate responsive devices for activating or deactivating cooling based on thermal needs.^[^ ^39^ ^]^ Another solution is to investigate thermochromic vanadium oxide (VO_2_) photonic structures that dynamically adjust radiative cooling capacity according to environmental changes.^[^ ^40^, ^41^, ^42^ ^]^

### 2.5 Durability and Self‐Cleaning Performance

**Figure 4:**
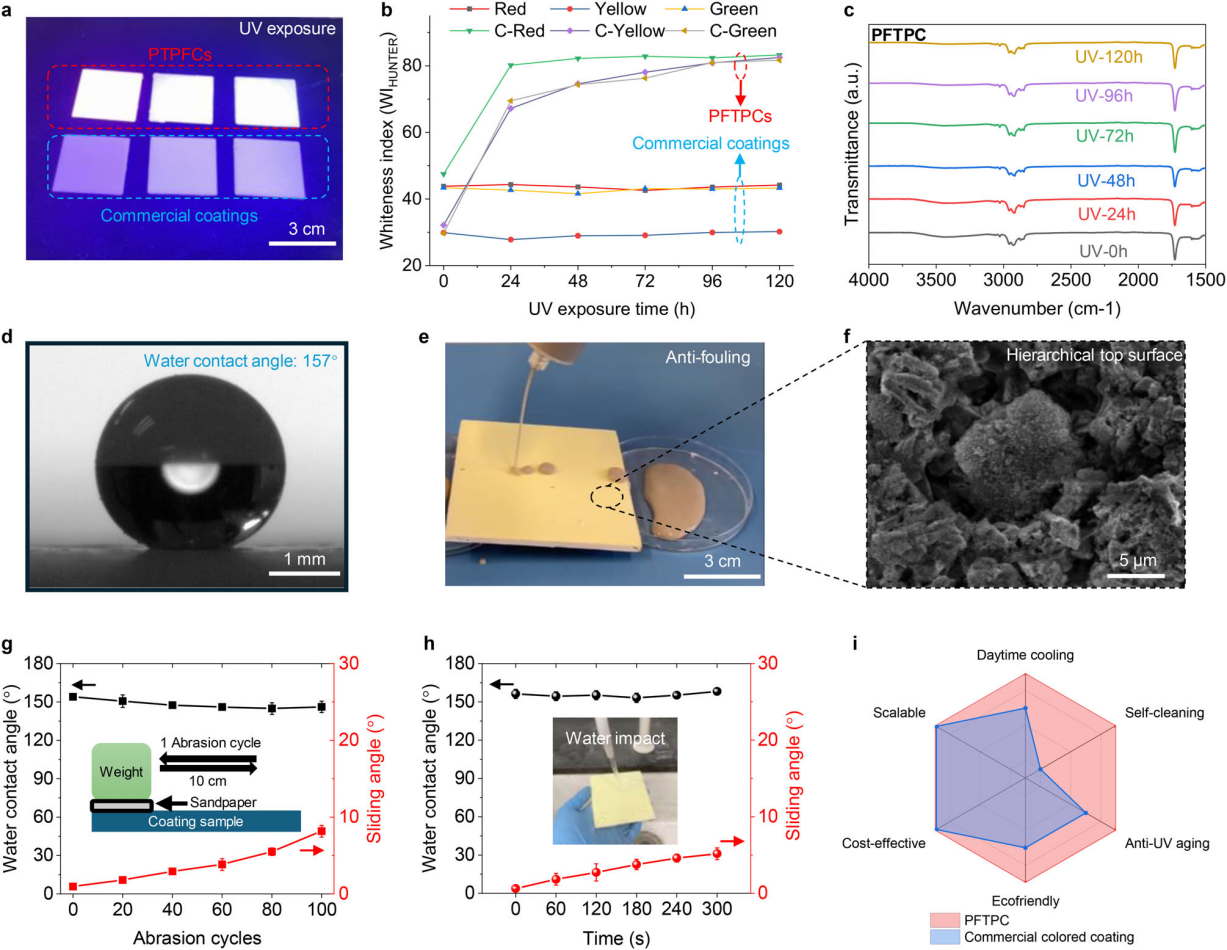
Durability, self‐cleaning, and performance comparison. a) Photographs of the PFTPCs under UV light (upper row), compared with commercial colored counterparts (lower row). b) Whiteness indexes variation of the PFTPCs compared with commercial counterparts during the UV exposure test. c) FTIR spectra of the PFTPCs during the UV exposure test. d) Optical image of the water contact angle of the PFTPC. e) Photograph of the antifouling test. f) Top surface morphology of the PFTPCs. g) Water contact angle and sliding angle variations during the mechanical abrasion test. h) Water contact angle and sliding angle variations during the water impact test. i) 6D performance comparison between the PFTPC and commercial coating counterpart.

The anti‐UV performance of the PFTPCs was evaluated through UV exposure tests and compared with commercial counterparts, as shown in **Figures** **4**a and S38 (Supporting Information). The highly efficient UV‐to‐visible fluorescence conversion ability of phosphors embedded in polymer matrix contributes a shining appearance of the PFTPCs, which could also mitigate common UV‐induced degradation in organic polymer coating materials (Figure S39, Supporting Information), thereby enhancing the durability and long‐term performance of these polymeric fluorescent coatings. As shown in Figure 4b, the whiteness indexes of the colored fluorescent coatings remained stable after 120 h of UV exposure, underscoring their high resistance to color fading. In contrast, commercial counterparts exhibited significant whitening within the first 24 h, which can be ascribed to the UV‐induced degradation of organic dyes in commercial colored paints (Figure S38c, Supporting Information). Additionally, Fourier‐transform infrared (FT‐IR) spectroscopy revealed that the composition of the polymer matrix in the colored fluorescent coatings remained almost unchanged after 120 h of UV exposure (Figure 4c), suggesting a potential service life exceeding one year under Hong Kong climatic conditions (Estimation details in Supporting Information).

The PFTPCs exhibit highly superhydrophobic wetting properties (Figure 4d) and excellent antifouling performance (Figure 4e; Movie S1, Supporting Information). Figure 4f presents a hierarchical micro/nano pillar surface morphology of the PFTPCs. The uppermost silanized SiO_2_ NP layer facilitates surface roughness (Figure S40, Supporting Information), enhancing the water contact angle (WCA) from 139° to 157° and achieving a nearly perfect sliding angle (SA) of 0.5°. Moreover, it can remove solid dust particles with the assistance of water droplets (Figure S41 and Movie S2, Supporting Information), repelling representative dust in diverse climate zones, including moderate, hot and humid, as well as hot and dry zones (Movie S3, Supporting Information). To assess the mechanical robustness of the PFTPCs, a mechanical abrasion test was conducted. Additionally, Figure 4g indicates that a high WCA of 150° and a low SA of less than 10° could be maintained after 100‐cycle mechanical abrasion tests. Considering the rainy weather in real‐world applications, a high‐speed water impact test was performed (Figure S42 and Movie S4, Supporting Information). The results showed that a high WCA of over 150° and a low SA of less than 6° were preserved during the water impact test (Figure 4h), confirming the practicality and robustness of the PFTPCs.

Furthermore, we evaluated our PFTPCs and commercial coating counterparts across six dimensions, including daytime cooling capacity, scalability, cost‐effectiveness, eco‐friendliness, UV tolerance, as well as self‐cleaning properties (Figure 4i). The material cost estimation for the PFTPCs is shown in Table S8 (Supporting Information). The lab‐scale cost is ≈6.7 $ m^−2^, which is comparable to commercial polymer coatings^[^ ^43^ ^]^ and is expected to decrease with large‐scale production. Additionally, we compared our PFTPCs with recently reported colored radiative coolers (Table S9, Supporting Information), demonstrating the superiority of PFTPCs in various aspects, including cooling capacity, self‐cleaning properties, cost, and scalability. Based on comparative analyses, the PFTPCs exhibit a significant radiative cooling effect, coupled with superior sustainability, scalability for large‐scale applications, and substantial commercialization potential.

## 3 Conclusion

The photon‐engineered fluorescent tri‐layer polymeric cooling coatings achieve high effective solar reflectance of 94%, 96.3%, and 96.1% for red, yellow, and green colors, respectively, enabled by Purcell‐enhanced fluorescent emission. Additionally, the PFTPCs exhibit high infrared emittance exceeding 96% in the atmospheric window, achieving daytime sub‐ambient cooling by 5.4–7.2 °C and superior radiative cooling performance compared to commercial colored coating counterparts in outdoor field tests (3.7–5.1 °C cooler). The theoretically calculated effective solar reflectivity properties for PFTPCs show a good agreement with the experimentally fitted effective solar reflectance. Building modeling further corroborates the substantial energy‐saving potential of the PFTPCs as roof and wall coatings. Our PFTPCs also demonstrate excellent self‐cleaning properties, including a strong anti‐fouling capability and high resistance to solid dust and various soiling. Additionally, the PFTPCs exhibit high durability, with exceptional anti‐UV ageing capabilities, resistance to mechanical abrasion, and high‐speed water impact. A comprehensive assessment confirmed the PFTPCs’ superior performance over their commercial counterparts in terms of cooling capacity, scalability, eco‐friendliness, and other aspects, indicating their sustainability and commercial potential. This work addresses the intricate design challenges of high‐performance fluorescence‐assisted colored radiative cooling coatings, advancing their applications and contributions to sustainable societal development.

## 4 Experimental Section

### Fabrication of Photon‐Engineered Fluorescent Tri‐Layer Polymeric Coatings (PFTPCs)

The fabrication of fluorescent tri‐layer polymeric coatings proceeded from bottom to up. Initially, the optimal white bottom layer was prepared by mixing 10 g poly‐styrene‐acrylic emulsion (solid content: ≈50%, emulsion density: ≈1.02 g cm^−3^, EC702, BASF Co. Ltd.), 65 g BaSO_4_ nanoparticles (Shenzhen Haiyang Powder Technology Co. Ltd.), and an appropriate amount of water was first mixed in a 200 mL plastic beaker with continuous stirring. To this mixture, 0.1 g dispersant agent (SN5040, SAN NOPCO Co., Ltd) and 0.2 g rheology modifying agent (Polyurethane, Guang Zhou Run Hong Chemical Co., Ltd) were added and stirred at 800 rpm for 1 h. Subsequently, the stirring speed was reduced to 400 rpm, and 0.3 g anti‐foaming agent (NXZ, SAN NOPCO Co., Ltd) and 0.8 g film‐forming agent (TEXNOL, Eastman Chemical Co., Ltd) were added to minimize micropores. After 30 min of stirring, the mixture was evenly applied on a cement board with a spray gun under a pressure of 3 MPa. The thickness of the bottom layer was controlled at ≈500 µm, confirmed by micrometers, according to the standard of ASTM D1005‐95. The coating thickness was measured using a digital display micrometer (SAN LIANG Co., Ltd.) with a precision of ± 1 µm, by averaging the results from three positions on each sample.

Then, the preparation of the colored middle layers with phosphors was conducted as follows: 10 g poly‐styrene‐acrylic emulsion, 19 g Y_2_O_3_ nanoparticles (Guang Zhou Metal Metallurgy Co., Ltd), phosphors (4.3 g for yellow and 6.4 g for green, purchased from Shenzhen Looking Long Technology), and an appropriate amount of water were initially mixed in a 100 mL plastic beaker with continuous stirring. Subsequently, 0.1 g dispersant and 0.1 g leveling agents were added. The mixture was stirred at 800 rpm for an hour, after which the stirring speed was reduced to 400 rpm, and 0.4 g antifoaming agent, along with 1 g film‐forming agent, were added. After an additional 30 min of stirring, the mixture was evenly sprayed onto the white bottom layer using a spray gun at a pressure of 3 MPa for 5 s. For the red middle layer, 10 g poly‐styrene‐acrylic emulsion, 41 g Y_2_O_3_ NPs, and 2.4 g red phosphors were used, following the same preparation procedures as for the yellow and green layers. The thickness of the colored layer was maintained at ≈20 µm, as confirmed by the cross‐sectional image of the eventual tri‐layer coating (Figure 2b) and micrometer measurements. The photographs and density information of primary raw materials are provided in Figure S43 and Table S10 (Supporting Information), respectively.

Subsequently, the topmost ultrathin transparent layer atop the colored layer was fabricated as follows: 0.4 g 20‐nm SiO_2_ nanoparticles chemically modified by 3‐methacryloxypropyltrimethoxysilane (KH‐570, JINAN ZHIDING WELDING MATERIALS Co., Ltd), 0.8 g tetraethyl orthosilicate (TEOS, GUANGZHOU, RUISHI BIOTECH Co., Ltd), and 0.1 g ammonia (WABCAN Co., Ltd) was used as catalytic for the hydrolysis reaction were mixed with 10 g ethanol. After 30 min of stirring, the mixture was sprayed evenly on the colored layer with a spray gun under a pressure of 3 MPa for 3 s. The thickness was controlled to be ≈3 µm since this self‐cleaning enhancing layer was attached onto the colored layer and helps to construct hierarchical surface micro/nano structures and exhibits superhydrophobicity.

### Fabrication of Commercial Colored Coating Counterparts

To demonstrate the superior efficacy of fluorescence in enhancing solar reflectivity, colored bilayer commercial coating counterparts were prepared as follows: Commercial colored acrylic paint (20 g for red, 14 g for yellow, and 5 g for green) from JIALIAN series (Colored latex paints, Dulux, Co. Ltd.) was hybridized with 10 g of white commercial acrylic paint to approach the color appearance of the PFTPCs (Figure S30, Supporting Information). The mixture was stirred at 800 rpm for 1 h. Subsequently, the stirring speed was reduced to 400 rpm, and 0.4 g anti‐foaming agent and 1 g film‐forming agent were added. After 30 min of additional stirring, the mixture was evenly sprayed onto the white bottom layer using a spray gun at a pressure of 3 MPa for 5 s, achieving a colored layer thickness of ≈20 µm. The white bottom layer for all commercial coating counterparts was identical to that of the PFTPCs.

### Material Characterizations

The cross‐section of coating samples was characterized by an FEI Quanta 450 Field‐Emission Scanning Electron Microscopy under a voltage of 15 kV, and elemental maps were obtained by performing energy dispersive spectroscopy (EDS). The size distributions of nanoparticles were obtained using a Malvern Mastersizer 3000 Particle Size Analyzer. FT‐IR spectra were obtained by a Thermo Fisher Nicolet iS10 FTIR spectrophotometer (Thermo Fisher, Germany) to analyze the functional groups and corresponding characteristic peaks of coating samples during the UV exposure test.

### Optical Characterizations

The solar reflectivity of nonfluorescent coating samples was measured on a PerkinElmer Lambda 1050+ UV/VIS/NIR Wide Band Spectrometer equipped with an integrating sphere. The infrared emissivity of coating samples in the wavelength range of 2.5–15.4 µm was measured by a Fourier transform infrared spectrometer (Nicoleti S50, Thermo Fisher Scientific) coupled with a transmittance attachment and a gold‐coated integrating sphere. Excitation spectra, emission spectra, PL lifetime, and quantum yield of pure phosphors/fluorescent cooling coatings were collected using the Edinburgh Instruments FLS900 Fluorescence spectrometer.

### Color Calculation

To characterize chromaticity according to the matching functions in the CIE XYZ system,^[^ ^44^ ^]^ the tristimulus values *X*, *Y*, and *Z* were first calculated to represent the response level of human eyes to incident light. The spectra *I*(λ) of Illuminant D65,^[^ ^14^ ^]^ which represents standard open illumination system conditions for color characterization, was then used for further calculations. With the reflectance spectra of the cooler *R*(*λ*) and color‐matching functions^[^ ^44^ ^]^ $\bar{x}\left(\lambda \right)$, $\bar{y}\left(\lambda \right)\;$ and $\bar{z}\left(\lambda \right)$, *X*, *Y*, and *Z* can be obtained by the equations below:

(3) $$X=100\frac{\int I\left(\lambda \right)R\left(\lambda \right)\bar{x}\left(\lambda \right)d\lambda }{\int I\left(\lambda \right)\bar{y}\left(\lambda \right)d\lambda }$$

(4) $$Y=100\frac{\int I\left(\lambda \right)R\left(\lambda \right)\bar{y}\left(\lambda \right)d\lambda }{\int I\left(\lambda \right)\bar{y}\left(\lambda \right)d\lambda }$$

(5) $$Z=100\frac{\int I\left(\lambda \right)R\left(\lambda \right)\bar{z}\left(\lambda \right)d\lambda }{\int I\left(\lambda \right)\bar{y}\left(\lambda \right)d\lambda }$$

The chromaticity of color was represented by the two normalized values (*x* and *y*) calculated with Equation (6) and Equation (7), which were derived from the tristimulus values *X*, *Y*, and *Z*. Then, the coordinate of the measured color was mapped within the CIE 1931 color space:

(6) $$x=\frac{X}{X+Y+Z}$$

(7) $$y=\frac{Y}{X+Y+Z}$$

### Outdoor Cooling Test

Outdoor cooling measurements were conducted in Kowloon, Hong Kong (22.4° N, 114.2° E) on 13‐14 April 2024 and 6 January 2025. Real‐time temperatures of coating samples were recorded using a MEMORY HiLOGGER LR8431‐30 multichannel temperature collector with K‐type thermocouples. Solar irradiation intensity and relative humidity were accurately recorded by a pyranometer (EKO MS‐802) and a mini weather station. The ambient temperature is measured with a thermocouple placed in a louvered box, simulating a Stevenson screen to avoid direct sunlight and facilitate thermal convection with ambient air through passive ventilation.^[^ ^45^ ^]^

### Surface Wetting Behavior Characterization

To characterize the surface‐wetting behavior of the PFTPCs, a movie‐based contact angle measurement system (DataPhysics Contact Angle Tester) was used to measure the contact angles and sliding angles using a sessile drop method. The liquid droplet volume was 5 µL for the contact angle measurements.

### Self‐Cleaning Performance Evaluations

Mud was formulated by mixing dust and water collected outdoors, and then poured over the PFTPCs to investigate soiling effects. The antifouling effect can be found in Figure 4e and Movie S1 (Supporting Information). Additionally, a solid soiling test was conducted using carbon black to demonstrate the self‐cleaning effect with water (Movie S2, Supporting Information). The soiling resistance of developed PFTPCs was evaluated in accordance with the standard of ASTM D7897‐18. Four soiling agents, mineral dust, inorganic salts, humic acids, and carbon black−representing atmospheric particles such as dust, salts, particulate organic matter (POM), and soot, were prepared to mimic natural soiling. As shown in Figure S45 (Supporting Information), 0.3 g Fe_2_O_3_ particles, 1.0 g montmorillonite K10 powders, and 1.0 g bentonite nano clay were dispersed into 1.0 L of water to prepare the mineral dust suspension. 0.3 g sodium chloride (NaCl), 0.3 g sodium nitrate (NaNO_3_), and 0.4 g calcium sulfate dihydrate (CaSO_4_·2H_2_O) were dissolved into 1.0 L of water to prepare the inorganic salt solution. 1.4 g of humid acid was dissolved into 1.0 L of water to prepare the POM solution. 0.26 g carbon black was dispersed into 1.0 L of water and stirred for 10 min to prepare the soot suspension. Given the variations in natural soiling across different climates, three mixtures of these four soiling agents (i.e., mineral dust, inorganic salts, humic acid, and carbon black) were prepared to simulate the three typical climates suitable for the application of the radiative cooling materials, namely, hot and dry (e.g., Xian, Shanxi province), hot and humid (e.g., Hong Kong), and moderate (i.e., hot summer and cold winter, e.g., Hefei, Anhui province), respectively. The mixing proportions of these three mixtures are provided in Table S12 (Supporting Information). The soiling resistance effect can be seen in Movie S3 (Supporting Information).

### UV Exposure Test

The UV exposure test involved subjecting the PFTPCs and their commercial counterparts to intense UV irradiation (175 W m^−2^, 60 °C). FT‐IR spectrometry and color characterization were performed after every 24 h of continuous UV exposure. The solar reflectivity of commercial counterparts was tested for the pristine state and after 120‐h UV exposure. Additionally, to estimate the influence of UV irradiation on the color appearance of coatings, the whiteness index and yellowness index were represented using the whiteness formula with CIE LAB coordinates^[^ ^46^ ^]^ and CIELAB b^*^, respectively. The CIE LAB coordinates can also be measured by a colorimeter (LS171, LINSHANG, Co, Ltd).

(8) $$WI_{\mathrm{HUNTER}}=L^{*}-3b^{*}$$

### Abrasion Test

The linear abrasion test of the PFTPCs was conducted using standard 240‐grit sandpaper with a 100 g abrading load,^[^ ^47^ ^]^ as illustrated in Figure 4g. During the abrasion test, variations in water contact angles and sliding angles at the three points along the abrasion path on the coating sample were recorded at every 20‐cycle abrasion.

### Water Impact Test

A water jet with a high speed (≈80 ml s^−1^) out of a faucet (10 cm from a nozzle with a diameter of 0.8 cm to the sample) impacted the coating surface, as can be seen in Figure S42 and Movie S4 (Supporting Information). Additionally, the water contact angles and sliding angles were measured at three different locations of the coating sample every 60 s during the water impact test.

## Conflict of Interest

The authors declare no competing interests.

## Supporting information



Supporting Information

Supplemental Movie 1

Supplemental Movie 2

Supplemental Movie 3

Supplemental Movie 4

## Data Availability

The data that support the findings of this study are available from the corresponding author upon reasonable request.
