# Supplementary material for: Innovative Photon‐Engineered Fluorescent Tri‐Layer Polymeric Coatings for Sub‐Ambient Colored Radiative Cooling
Source: Adv Sci (Weinh). 2025 Sep 3;12(43):e11599. doi: 10.1002/advs.202511599 (PMC12631870; doi:10.1002/advs.202511599)
Supplement: Supplementary file 1 — Supporting Information [file ADVS-12-e11599-s001.docx]

Supporting Information for

**Innovative photon-engineered fluorescent tri-layer polymeric coatings for sub-ambient colored radiative cooling**

Tao Wang, Ying Liu, Qingdong Xuan, Ma Xue, Yanbo Fang, You Dong, Dangyuan Lei * and Jian-Guo Dai **

**Table of Contents**

**S1. Supplementary Texts**

S1.1. Theoretical modeling of the scattering and absorption properties of the BaSO_4_ and Y_2_O_3_ particles

S1.2. Calculation of solar reflectance and LWIR emittance

S1.3. Heat transfer analysis for fluorescent radiative coolers

S1.4. The modified Monte Carlo method for spectral properties of coatings with fluorescent pigments

S1.5. Validation of the modified Monte Carlo method

S1.6. PL lifetime decay of phosphors embedded in the coating

S1.7. Estimation of UV aging time for the PFTPC in real-world applications

S1.8. Fitting of effective solar reflectance

**S2. Supporting Figures**

Figure S1 to S45

**S3. Supporting Tables**

Table S1 to S12

**S4. Supporting Movies**

Movie S1 to S4

**S5. References**

# S1. Supplementary Texts

## S1.1. Theoretical modeling of the scattering and absorption properties of the BaSO_4_ and Y_2_O_3_ particles

The scattering efficiency was calculated to optimize the nanoparticle size in the white bottom and colored middle layer. According to Mie theory,^[1]^ the scattering efficiency $Q_{\mathrm{sca}}$, extinction efficiency $Q_{\mathrm{ext}}$, and absorption efficiency$Q_{\mathrm{abs}}$, were determined by solving Maxwell equations: ^[1]^

$\begin{aligned} Q_{\mathrm{sca}}=\frac{2}{x^{2}}\sum_{n=1}^{\infty} \left( 2n+1 \right)\left( \left| a_{n} \right|^{2}+\left| b_{n} \right|^{2} \right) \#\left( 1 \right) \end{aligned}$

$$\begin{aligned} Q_{\mathrm{ext}}=\frac{2}{x^{2}}\sum_{n=1}^{\infty} \left( 2n+1 \right)\mathrm{Re}\left\{ a_{n}+b_{n} \right\}\#\left( 2 \right) \end{aligned}$$

$$\begin{aligned} Q_{\mathrm{abs}}=Q_{\mathrm{ext}}-Q_{\mathrm{sca}}\#\left( 3 \right) \end{aligned}$$

where *x* is the size parameter defined by the particle size wavelength and refractive index of coating matrix, *a*_n_ and *b*_n_ are Mie coefficients.

The refractive index of coating constituents (**Figure S1**) allows for the calculation of the scattering and absorption efficiencies of BaSO_4_ nanoparticles (NPs) of varied sizes, as illustrated in **Figure S2**. For the bottom layer, BaSO_4_ NPs with a mean diameter of 1.5 µm were chosen to provide high solar scattering and exploit phonon resonance to thermal radiation. For the colored layer, Y_2_O_3_ NPs with a mean diameter of 1 µm were selected to boost fluorescence re-emission of phosphors embedded in the coating matrix via Purcell enhancement.^[2]^ Efficient Purcell enhancement can be achieved by matching the scattering efficiencies of Y_2_O_3_ NPs to the emission peak wavelengths of the phosphors (**Figure S6**).

## S1.2. Calculation of solar reflectance and LWIR emittance

The solar reflectance (*R*_solar_) is the ratio of the reflected solar irradiation in the *λ* of 0.25-2.5 µm to the total input of solar irradiation energy in this wavelength range:^[3]^

$$\begin{aligned} R_{\mathrm{solar}}=\frac{\int_{0.25 \mu m}^{2.5 \mu m} I_{\mathrm{solar}}\left( \lambda\right)R\left( \lambda\right)d\lambda}{\int_{0.25 \mu m}^{2.5 \mu m} I_{\mathrm{solar}}\left( \lambda\right)d\lambda}\#\left( 4 \right) \end{aligned}$$

where *I*_solar_(*λ*) denotes the ASTM G173-03 Global solar intensity spectrum, and *R*(*λ*) is the spectral reflectance of the cooler.

The long-wave infrared (LWIR) emittance ($\varepsilon_{\mathrm{LWIR}}$) refers to the ratio of the thermal radiation power emitted by the cooler within the atmospheric window, typically encompassing wavelengths between 8 and 13 µm, to the integral of the thermal radiation intensity emitted by a standard blackbody over this wavelength range:

$$\begin{aligned} \varepsilon_{\mathrm{LWIR}}=\frac{\int_{8\mu m}^{13\mu m} I_{\mathrm{bb}}\left( T,\lambda\right)\varepsilon\left( T,\lambda\right)d\lambda}{\int_{8\mu m}^{13\mu m} I_{\mathrm{bb}}\left( T,\lambda\right)d\lambda}\#\left( 5 \right) \end{aligned}$$

where *ε* (*T*, *λ*) represents the spectral emittance of the cooler and *I*_bb_ (*T, λ*) refers to the standard blackbody radiation intensity at a temperature of *T*, which can be calculated by Planck’s law:

$$\begin{aligned} I_{\mathrm{bb}}\left( T,\lambda\right)=\frac{2c^{2}\hbar}{\lambda^{5}}\frac{1}{e^{\frac{\hbar c}{\lambda k_{B}T}}-1}\#\left( 6 \right) \end{aligned}$$

where *k*_B_ is the Boltzmann constant, *c* is the speed of light in a vacuum environment, $\hbar$ is the Planck constant.

## S1.3. Heat transfer analysis for fluorescent radiative coolers

To quantify the cooling performance of the cooler, including performance metrics such as net cooling power and achievable temperature reduction relative to the ambient temperature, the thermal equilibrium of a daytime fluorescent radiative cooler has been comprehensively analyzed, as shown in **Figure S7**a. The effective cooling power, denoted as *P*_cool,eff_, can be calculated as follows:^[4]^

$$\begin{aligned} P_{cool,\mathrm{eff}}\left( T_{c} \right)=P_{\mathrm{rad}}\left( T_{c} \right)-P_{\mathrm{atm}}\left( T_{\mathrm{amb}} \right)-P_{solar,\mathrm{eff}}-P_{conv+cond}\left( T_{c}, T_{\mathrm{amb}} \right)\#\left( 7 \right) \end{aligned}$$

where *P*_rad_, *P*_solar_, and *P*_atm_ are the thermal radiation power by the cooler, the absorbed power from solar irradiation, and the absorbed power from downwelling atmospheric radiation, respectively. *P*_conv+cond_ is the cooling power loss caused by heat convection and conduction process of the cooler with surrounding environment. *T*_c_ and *T*_amb_ are the temperatures of cooler and ambient temperature, respectively.

In Eq. (7), *P*_rad_ has been resolved as follows:

$$\begin{aligned} P_{\mathrm{rad}}\left( T_{c} \right)=2\pi\int_{0}^{\frac{\pi}{2}} sin\theta cos\theta\int_{0}^{\infty} I_{\mathrm{bb}}\left( T_{c},\lambda\right)\varepsilon\left( \lambda,\theta\right)d\theta d\lambda\#\left( 8 \right) \end{aligned}$$

where *I*_bb_(*T*_c_*, λ*) is shown in Eq. (7). *ε*(*λ,θ*) represents the emissivity of the cooler and varies with wavelength and direction.

Regarding *P*_atm_:

$$\begin{aligned} P_{\mathrm{atm}}\left( T_{\mathrm{amb}} \right)=2\pi\int_{0}^{\frac{\pi}{2}} sin\theta cos\theta\int_{0}^{\infty} I_{\mathrm{bb}}\left( T_{\mathrm{amb}},\lambda\right)\varepsilon_{\mathrm{atm}}\left( \lambda,\theta\right)\varepsilon\left( \lambda,\theta\right)d\theta d\lambda\#\left( 9 \right) \end{aligned}$$

$\varepsilon_{\mathrm{atm}}\left( \lambda,\theta\right)$ represents the atmospheric emittance, which can be obtained with the atmospheric transmittance *t*(*λ*) given by $\varepsilon_{\mathrm{atm}}\left( \lambda,\theta\right)=1-{t\left( \lambda\right)}^{1/cos\theta}$. Here, *t*(*λ*) is computed by the MODTRAN modeling.^[5]^

*P*_solar, eff_ denotes the effective solar heat intake, the formula is given by:

$$\begin{aligned} P_{solar, eff}=Incident solar flux-backward energy flux \\ =\int_{0}^{\infty} \left[ 1-\mathrm{ESR}\left( \lambda\right) \right]I_{\mathrm{solar}}\left( \lambda\right)d\lambda\#\left( 10 \right) \end{aligned}$$

where $\mathrm{ESR}\left( \lambda\right)$ refers to Eq. (1) in the main text, *I*_solar_ is the local solar irradiation.

Separately, *P*_conv+cond_ is expressed as:

$$\begin{aligned} P_{conv+cond}\left( T_{c},T_{\mathrm{amb}} \right)= h_{c}\left( T_{\mathrm{amb}}-T_{c} \right)\#\left( 11 \right) \end{aligned}$$

where *h*_c_ is the non-radiative heat transfer coefficient caused by heat convection and conduction effect of the cooler with surrounding environment.

In the initial state, the cooling power is calculated using Eq. (10) when *T*_c_ = *T*_amb_. Additionally, the final steady-state temperature of the cooler is computed by setting *P*_cool_ = 0 in Eq. (10) once the radiative cooler reaches thermal equilibrium. **Figure S7**b demonstrates the impact of the nonradiative heat transfer coefficient on the temperature reduction capability of the ideal selective radiative cooler (SRC) and the broadband radiative cooler (BRC). It is apparent that nonradiative heat transfer significantly reduces the SRC’s cooling effect. For instance, when *h*_c_ is 10 W m^-2^ K^-1^, the temperature reduction effects of the SRC and the BRC are comparable. However, when *h*_c_ is reduced to 0 W m^-2^ K^-1^, the SRC achieves a substantially lower temperature, approximately 40 ℃ lower than the BRC. Regarding the effects of solar reflectance and LWIR emittance on cooling power, **Figure S7**c indicates that solar reflectance exerts a considerably more significant effect than LWIR emittance.

To validate the accuracy of fitted effective solar reflectance of fluorescent coatings, an energy balance analysis was conducted for the steady-state temperature. Atmospheric transmittance in tropical urban areas condition was modeled by MODTRAN program,^[5]^ with a total water column of 3000 atm-cm for 50-60% relative humidity (RH). *h*_c_ was set to 6.9 W/m^2^/k, considering wind coverage and a similar holder configuration in outdoor tests as in Raman’s work,^[4]^ and all coating samples were tested the identical heat conduction conditions in outdoor cooling tests. The theoretical temperature values for three fluorescent coatings were calculated at each datapoint to satisfy the conservation Eq. (7) and are shown in **Figure S35** against the measured temperatures, which fit well with the recorded data.

## S1.4. The modified Monte Carlo method for spectral properties of coatings with fluorescent pigments

To estimate the spectral properties of coating structures with fluorescent pigments, a modified Monte Carlo (MMC) method was developed to tackle the fluorescence conversion process and trace the photon’s destiny in the multilayer coating system, including absorption, reflection and transmission. Building on Fu et al.’s ray-tracing framework for multilayer coating structures^[6]^ and Yalçın’s logic for considering fluorescence emission in coating matrix,^[7]^ we focused on modifying the ray-tracing in the layer that contains fluorescent pigments. For the colored layer with phosphors, the absorption coefficient of the composite, phosphors and remaining non-fluorescent constituents (i.e., polymer matrix and Y_2_O_3_ NPs), are denoted as *μ*_a_, *μ*_af_ and *μ*_anf_, respectively. The same naming rule applies to the scattering coefficients, for instance, the scattering coefficient of the composite is *μ*_s_.

Thus,

$$\begin{aligned} \mu_{s}=\mu_{\mathrm{snf}}+\mu_{\mathrm{sf}}\#\left( 12 \right) \end{aligned}$$

$$\begin{aligned} \mu_{a}=\mu_{\mathrm{anf}}+\mu_{\mathrm{af}}\#\left( 13 \right) \end{aligned}$$

For the dependent scattering induced by high concentration of nanoparticles (>5 vol.%), a simplified correction can be utilized to consider its effect on scattering/absorption coefficients.^[8]^

$$\begin{aligned} \mu_{s,dep}=\mu_{s}\left( 1+1.5\mathrm{fv}-0.75\mathrm{fv}^{2} \right)\#\left( 14 \right) \end{aligned}$$

$$\begin{aligned} \mu_{a,dep}=\mu_{a}\left( 1+1.5\mathrm{fv}-0.75\mathrm{fv}^{2} \right)\#\left( 15 \right) \end{aligned}$$

where fv is the volume fraction of nanoparticles. $\mu_{s,dep}$ and $\mu_{a,dep}$ are scattering and absorption coefficients, respectively, after correcting the dependent scattering effect.

So, the extinction coefficient of the composite is resolved as follows:

$$\begin{aligned} \mu_{e}=\mu_{a}+\mu_{s}\#\left( 16 \right) \end{aligned}$$

The probability of the photon absorption is quantified as follows:

$$\begin{aligned} P_{\mathrm{abs}}=\frac{\mu_{a}}{\mu_{e}}\#\left( 17 \right) \end{aligned}$$

Specifically, the probability that the photon is absorbed by either the other coating constituents or phosphors without generating fluorescence is determined as follows:^[9]^

$$\begin{aligned} \frac{\mu_{\mathrm{anf}}+\mu_{\mathrm{af}}\cdot\left( 1-\mathrm{PLQY} \right)}{\mu_{e}}\#\left( 18 \right) \end{aligned}$$

where PLQY is the photoluminescence quantum yield of the phosphor.

The probability that the photon is converted into fluorescence and re-emitted is:

$$\begin{aligned} P_{\mathrm{unconverted}}=\frac{\mu_{\mathrm{af}}\cdot\mathrm{PLQY}}{\mu_{e}}\#\left( 19 \right) \end{aligned}$$

Additionally, the probability that the photon is scattered by this fluorescent layer is:

$$\begin{aligned} P_{\mathrm{converted}}=\frac{\mu_{s}}{\mu_{e}}\#\left( 20 \right) \end{aligned}$$

When the photon is re-emitted, the pristine energy of photon at the excited wavelength is *Ф*_ex_, then changed to:

$$\begin{aligned} \phi_{\mathrm{em}}=\phi_{\mathrm{ex}}\cdot\frac{\lambda_{\mathrm{ex}}}{\lambda_{\mathrm{em}}}\#\left( 21 \right) \end{aligned}$$

where $\lambda_{\mathrm{ex}}$ and $\lambda_{\mathrm{em}}$ are the excitation and emission wavelength, respectively. $\frac{\lambda_{\mathrm{ex}}}{\lambda_{\mathrm{em}}}$ is the ratio of Stoke shift. Since the emission wavelength for re-emitted photon can be determined by probability density function (PDF) based on the emission spectra profile of corresponding phosphor (**Figure S10**), unit photon energy is utilized in each loop for sampled wavelength points during the modified Monte Carlo simulation. Additionally, the scattering direction of re-emitted photon will be randomized to approximate fluorescence conversion process of the phosphor (**Figure S11**).

Then the effective scattering and absorption properties of the *i*th layer can be expressed as:

$$\begin{aligned} \sigma_{i,\lambda}=\sum\frac{3f_{i,j}Q_{sca,j}}{4r_{j}}\#\left( 22 \right) \end{aligned}$$

$$\begin{aligned} \kappa_{i,\lambda}=\sum\frac{3f_{i,j}Q_{abs,j}}{4r_{j}}+\frac{4\pi k_{\mathrm{matrix}}}{\lambda}\#\left( 23 \right) \end{aligned}$$

The solar reflectance and infrared emittance spectra of the coating can be obtained by solving the modified quasi-steady form of the radiative transfer equation:^[10]^

$$\begin{aligned} \frac{dI_{\lambda}}{ds}=\kappa_{\lambda}I_{b,\lambda}-\left( \kappa_{\lambda}+\sigma_{\lambda} \right)I_{\lambda}+\frac{\sigma_{\lambda}}{4\pi}\int I_{\lambda}\left( \hat{S}_{i} \right)\Phi_{\lambda}\left( \hat{S}_{i},\hat{S} \right)d\Omega^{'}+S_{\lambda_{\mathrm{em}}}\#\left( 24 \right) \end{aligned}$$

where $I_{\lambda}$ and $I_{b,\lambda}$are the spectral radiative intensities of the coating and a blackbody, *S* denotes the geometric path of radiative transfer, $\Phi_{\lambda}\left( \hat{S}_{i},\hat{S} \right)$ is the scattering phase function describing the scattering probability from direction $\hat{S}_{i}$to direction $\hat{S}$ of the incoming heat flux at solid angle of $d\Omega^{'}$, $\sigma_{\lambda}$ and $\kappa_{\lambda}$ are the effective properties obtained by Eqs. (22) and (23).

$$\begin{aligned} S_{\lambda_{\mathrm{em}}}=\frac{1}{4\pi}\int_{0}^{\infty} I_{\mathrm{ex}}\mu_{\mathrm{af}}\mathrm{PLQY}\left( \lambda_{\mathrm{ex}} \right)\frac{\lambda_{\mathrm{ex}}}{\lambda_{\mathrm{em}}}P_{\mathrm{fl}}\left( \lambda_{\mathrm{em}} \right)d\lambda_{\mathrm{ex}}\#\left( 25 \right) \end{aligned}$$

where $I_{\mathrm{ex}}$ is the spectral intensities of the excited part of incident light, $P_{\mathrm{fl}}(\lambda_{\mathrm{em}})$ is the ratio of energy emitted at a particular wavelength $\lambda_{\mathrm{em}}$ to the total energy emitted obtained by Eq. (26). The energy emitted due to fluorescent excitation follows a distribution that typically characterized by normalized fluorescent intensity, $f_{\mathrm{fl}}(\lambda_{\mathrm{em}})$, which represents the fluorescent emission at a specific wavelength relative to its maximum value. The ratio of energy emitted at a particular wavelength, $\lambda_{\mathrm{em}}$, to the total energy emitted is defined as follows:

$$\begin{aligned} P_{\mathrm{fl}}\left( \lambda_{\mathrm{em}} \right)= \frac{f_{\mathrm{fl}}\left( \lambda_{\mathrm{em}} \right)}{\int_{0}^{\infty} f_{\mathrm{fl}}\left( \lambda_{\mathrm{em}} \right)d\lambda_{\mathrm{em}}}\#\left( 26 \right) \end{aligned}$$

The flow chart of our modified Monte Carlo simulation, designed to solve the radiative heat transfer equation of the multilayer coating with fluorescent pigments, is presented in **Figure S12**. In this model, random numbers (RN) are employed to statistically trace the light path. The effective properties of sublayers and the scattering characteristics of particles are identified using the layer flag (*i*) and particle type flag (*j*), respectively.

Complex refractive indexes of Y_3_Al_5_O_12_:Ce^3+^, Lu_3_Al_5_O_12_:Ce^3+^, and Sr_2_Si_5_N_8_:Eu^2+^ were extracted from references.^[11-14]^ Since the minimal rare-earth element is doped in phosphors,^[15]^ the refractive indexes of utilized phosphors within the MIR wavelength range were taken as 1.79 for Y_3_Al_5_O_12_:Ce^3+^, 1.72 for Lu_3_Al_5_O_12_:Ce^3+^, and 1.61 for Sr_2_Si_5_N_8_:Eu^2+^, respectively. Since the uppermost silanized SiO_2_ NP layer, being ultrathin (~3 µm) and solar-transparent, was excluded from the MMC simulation for the spectral properties of our tri-layer coatings. Spectral properties and color appearance of radiative cooling coatings with phosphors can be estimated using our MMC method. Indeed, the effective spectral response of fluorescent coating materials can be attained by our MMC method when provided with the illumination intensity spectra.

## S1.5. Validation of the modified Monte Carlo method

In this study, we designed colored fluorescent tri-layer polymeric coatings featuring a solar-transparent thin uppermost layer. To validate the accuracy of our MMC method for generic bilayer coating structures, we compared our simulated results with the experimental findings from Lin’s work.^[16]^ The simulated bilayer coating structure comprises 20 vol.% Al_2_O_3_ particles with a diameter of 250 nm in the top layer, and 25 vol.% TiO_2_ particles with a diameter of 200 nm in the bottom layer. The total thickness of bilayer coating is 500 µm, with a thickness ratio of the top layer to the bottom layer of 0.5.

In light of the precision of the extracted refractive index of Y_2_O_3_, we also conducted simulations for a PDMS-based coating structure with Y_2_O_3_ NPs in Luo et al.’s work.^[17]^ The simulated monolayered coating structure comprises 10 vol.% Y_2_O_3_ NPs with a diameter of 500 nm, and the monolayer’s thickness is 500 μm. The simulated results for these two coating cases are shown in **Figure S13**, which demonstrate the accuracy of out MMC method for predicting optical properties of non-fluorescent coating structures.

Furthermore, to valid simulated spectral properties of fluorescent coatings incorporating phosphors, we compared the experimentally fitted effective solar reflectance of fabricated colored coating with the integration values from three corresponding simulated effective solar reflectivity spectra. The comparison results are shown in **Figure S32**c-d, showing good agreement when considering a slight underestimation of the bottom’s solar reflectance by our MMC method.

Consequently, the validity of the modified Monte Carlo method for estimating optical properties of is confirmed, indicating it can severe as an effective tool for spectral properties prediction of both nonfluorescent and fluorescent coating materials.

## S1.6. PL lifetime decay of phosphors embedded in the coating

Since phosphor is surrounded by other nanoparticles, a Purcell photonic microcavity is formed, which enhances the emission rate and shortens PL lifetime. For Sr_2_Si_5_N_8_:Eu^2+^, two distinct host Sr^2+^ ion sites (Sr^2+^ 1 and Sr^2+^ 2) experiencing different crystal field strengths can be occupied by the Eu^2+^ ions (Eu^2+^ 1 and Eu^2+^ 2), resulting in different energy levels for the excited states 4f^6^5d^1^ of Eu^2+^ ions.^[18-20]^ For Y_3_Al_5_O_12_:Ce^3+^ and Lu_3_Al_5_O_12_:Ce^3+^, the doped Ce^3+^ are subjected to identical crystal field strengths.^[21-24]^ PL lifetimes of three phosphors are shown as **Figure S25**. The PL emission of the red phosphors Sr_2_Si_5_N_8_:Eu^2+^ can be fitted by two peaks, each corresponding to Eu^2+^ ions experiencing different crystal fields. Consequently, the PL lifetime of Sr_2_Si_5_N_8_:Eu^2+^ should be fitted tri-exponentially by the following equation:

$$\begin{aligned} I\left( t \right)=I_{0}+A_{1}\exp\left( -\frac{t}{\tau_{1}} \right)+A_{2}\exp\left( -\frac{t}{\tau_{2}} \right)+A_{3}\exp\left( -\frac{t}{\tau_{3}} \right)\#\left( 27 \right) \end{aligned}$$

where $\tau_{1}$ and $\tau_{2}$ are radiative lifetimes of two energy level transitions, respectively. $A_{1}$ and $A_{2}$ are corresponding amplitudes for the two radiative transitions. $\tau_{3}$ and $A_{3}$ are the non-radiative lifetime and amplitude. Different from the red phosphors, the PL emission of Y_3_Al_5_O_12_:Ce^3+^ and Lu_3_Al_5_O_12_:Ce^3+^ both come from the doped Ce^3+^ ions which experience the same crystal strength. Thus, the lifetime of these two phosphors should be fitted biexponentially by the following equation:

$\begin{aligned} I\left( t \right)=I_{0}+A_{1}\exp\left( -\frac{t}{\tau_{1}} \right)+A_{2}\exp\left( -\frac{t}{\tau_{2}} \right)\#\left( 28 \right) \end{aligned}$

where $\tau_{1}$ and $\tau_{2}$ are radiative lifetimes and nonradiative lifetime of two energy level transitions, respectively. $A_{1}$ and $A_{2}$ are corresponding amplitudes for radiative and nonradiative decay. **Table S1** shows that the radiative lifetime of $\tau_{1}$ and $\tau_{2}$ of the red phosphor were shortened by 36% and 20%, respectively, after being embedded into the coating matrix with 1-μm Y_2_O_3_ NPs, which indicate that the formed Purcell cavity achieves a highly efficient lifetime reduction. The fitted radiative and non-radiative time for green and yellow phosphor were listed in **Table S2** and **Table S3**, were shortened by 9% and 4% respectively.

## S1.7. Estimation of UV aging time for the PFTPC in real-world applications

The estimated duration of UV aging is influenced by various real-world factors, including daytime duration, solar irradiation intensity, ambient temperature, and humidity.^[25]^ Taking Hong Kong as an example, the annual solar irradiation duration is about 1870 hours with a total solar irradiation power of about 1420 kWh m^−2^ year^−1^ (Data was obtained from <https://www.weather.gov.hk/tc/publica/mws/files> (Hong Kong Observatory)). Additionally, UV radiation constitutes about 5% of the total solar irradiation. Consequently, the total annual UV radiation in this study was computed to be 71 kWh m^−2^ year^−1^. Subsequently, the average annual solar irradiation power per unit area was determined to be: 71 kWh m^−2^ year^-1^ ÷ 1870 h year^−1^ ≈ 38.0 W m^−2^. The radiation intensity under UV exposure test was 175 W m^−2^. By dividing this value by 38 W m^−2^, we arrived at a ratio of 4.61. Hence, it was estimated that the simulated radiation was about 4.61 times higher than outdoor UV irradiation in Hong Kong.

UV aging is intrinsically linked to temperature. Hong Kong experiences an average annual temperature of 24.5 ℃. The empirical evaluation of aging effects shows a 1.5-fold increase for every 1°C rise in temperature.^[26]^ Given that the actual test temperature exceeded 60°C due to continual UV irradiation, it was standardized to 60°C. Through the aforementioned analysis, a correlation between the estimated UV aging duration and accelerated UV aging time was derived as 4.61*60/24.5*1.5 = 16.93. Considering the 120-hour duration of UV irradiation testing in this study, the estimated UV aging time in real-world environments was computed as 120 hours *16.93 = 2032 hours (~85 days). Furthermore, the daily outdoor sunlight exposure lasts approximately 8 hours, excluding rainy and cloudy days. Consequently, the total estimated UV aging time stands at around 85 days *24/8 = 255 days. However, when accounting for cloudy and rainy days, the average annual outdoor sunlight irradiation time is about 1870 hours ÷ 365 days ≈ 5.1 hours per day, ensuring normal usage over 85 days *24/5.1 = 400 days (over one year).

## S1.8. Fitting of effective solar reflectance

Since effective solar reflectance cannot be accurately measured using a UV-visible-NIR spectrometer, the effective solar reflectance of all colored fluorescent coatings was determined by fitting the linear relationship between solar reflectance and temperature observed in all non-fluorescent coatings within the reference group:^[2, 27, 28]^

$$\begin{aligned} T=\frac{\left[ \left( 1-\mathrm{ESR} \right)I_{\mathrm{solar}}+h_{c}T_{\mathrm{amb}}+h_{\mathrm{rad}}T_{\mathrm{atm}} \right]}{h_{c}+h_{\mathrm{rad}}}\#\left( 29 \right) \end{aligned}$$

where $I_{\mathrm{solar}}$ is the solar irradiation intensity, $h_{c}$ and $h_{\mathrm{rad}}$ are non-radiative and radiative heat transfer coefficients, respectively; $T_{\mathrm{amb}}$ and $T_{\mathrm{atm}}$ are the temperature of the ambient and atmosphere, respectively. Then, this equation can be further expressed as follows:

$\begin{aligned} T=a\cdot\mathrm{ESR}+b\#\left( 30 \right) \end{aligned}$

where $a$ and $b$ are environmental parameters fitted from the solar reflectance of non-fluorescent coatings in the reference group including three commercial colored coating counterparts and the white bottom layer coating. To ensure the accuracy of fitted ESRs, the fitting range was chosen from 11:30 to 12:30 on 13 April 2024 due to the stable solar irradiation during this period (**Figure S31**a). The solar reflectivity data of coating samples in the reference group is provided in **Figure S44** and **Table S11**.

# S2. Supporting Figures

| 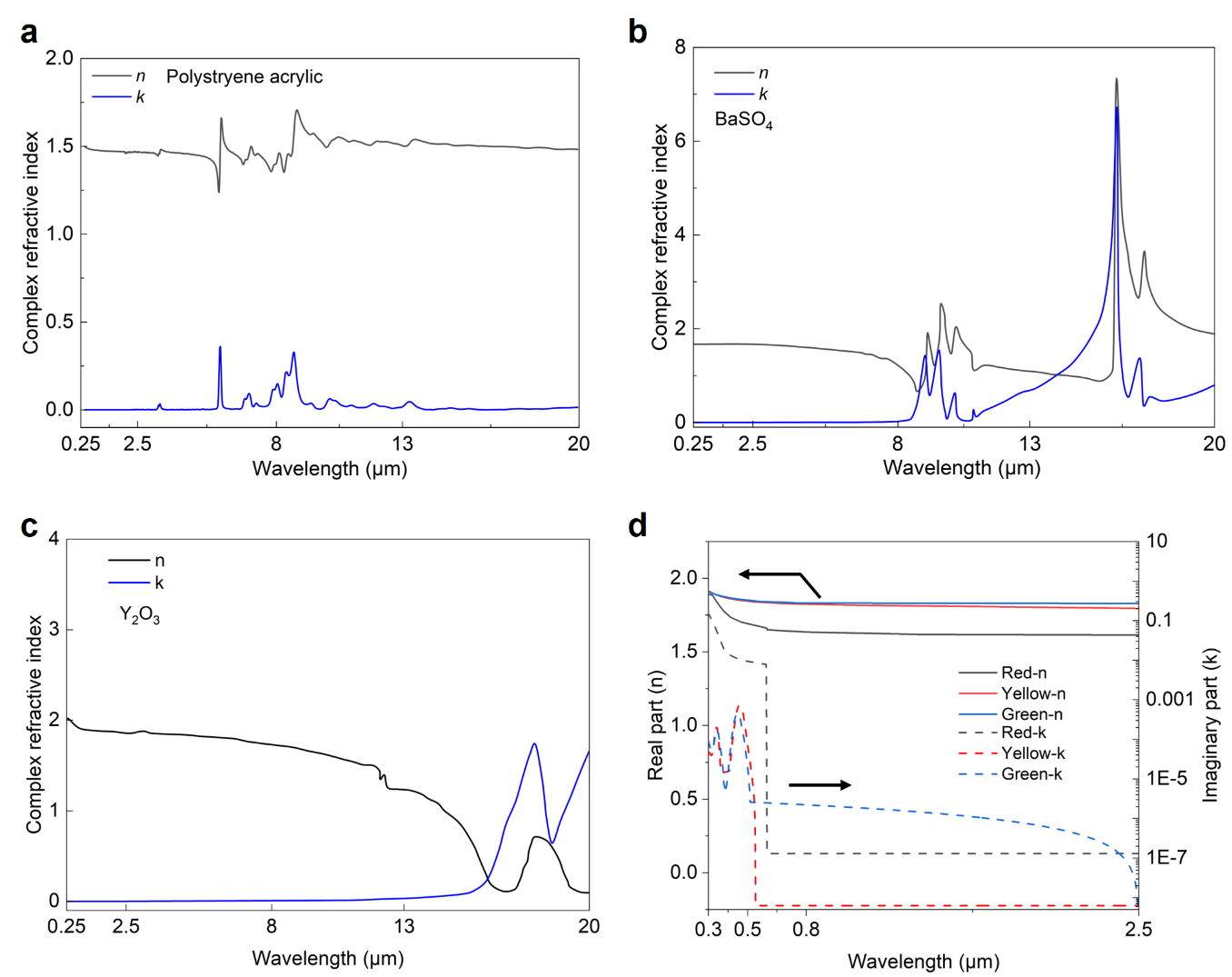 |
| --- |
| **Figure S1**. Complex refractive index of (a) poly-styrene-acrylic^[27]^. (b) BaSO_4_ particles^[29]^. (c) Y_2_O_3_ particles^[17]^. (d) the yellow phosphor (Y_3_Al_5_O_12_:Ce^3+^)^[11, 13]^, the green phosphor (Lu_3_Al_5_O_12_:Ce^3+^)^[14]^ and the red phosphor (Sr_2_Si_5_N_8_:Eu^2+^)^[12]^. |

| 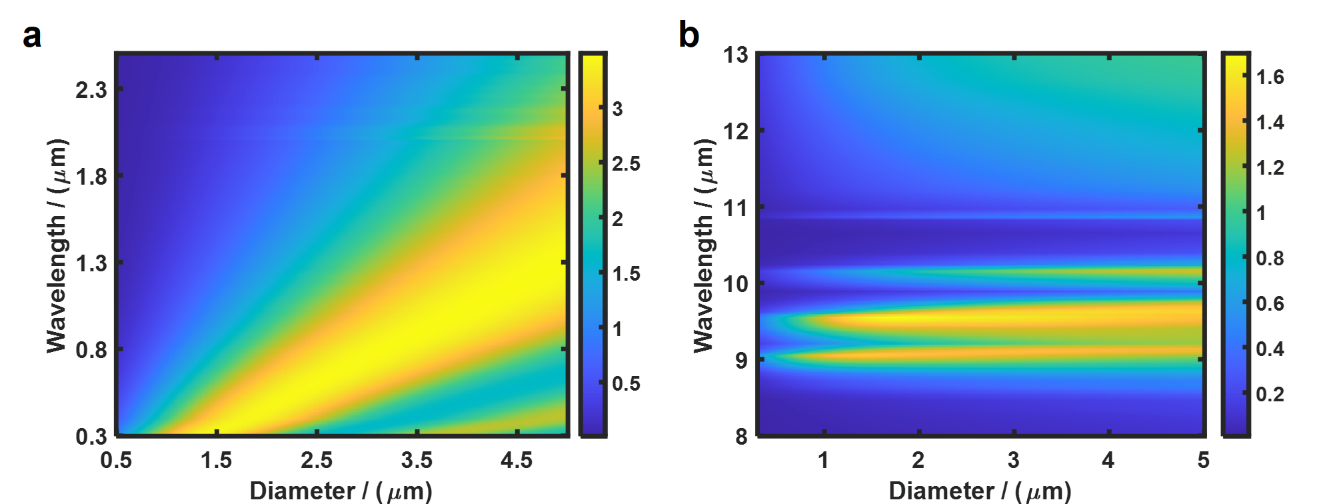 |
| --- |
| **Figure S2.** (a) Scattering efficiency and (b) absorption efficiency for the BaSO_4_ nanoparticle with diverse sizes embedded in poly-styrene-acrylic matrix. The BaSO_4_ nanoparticle with a size ranging from 1.5 to 2.5 µm enables efficient solar scattering across solar wavelengths while exhibits two absorption resonant peaks within the atmospheric window. |

| 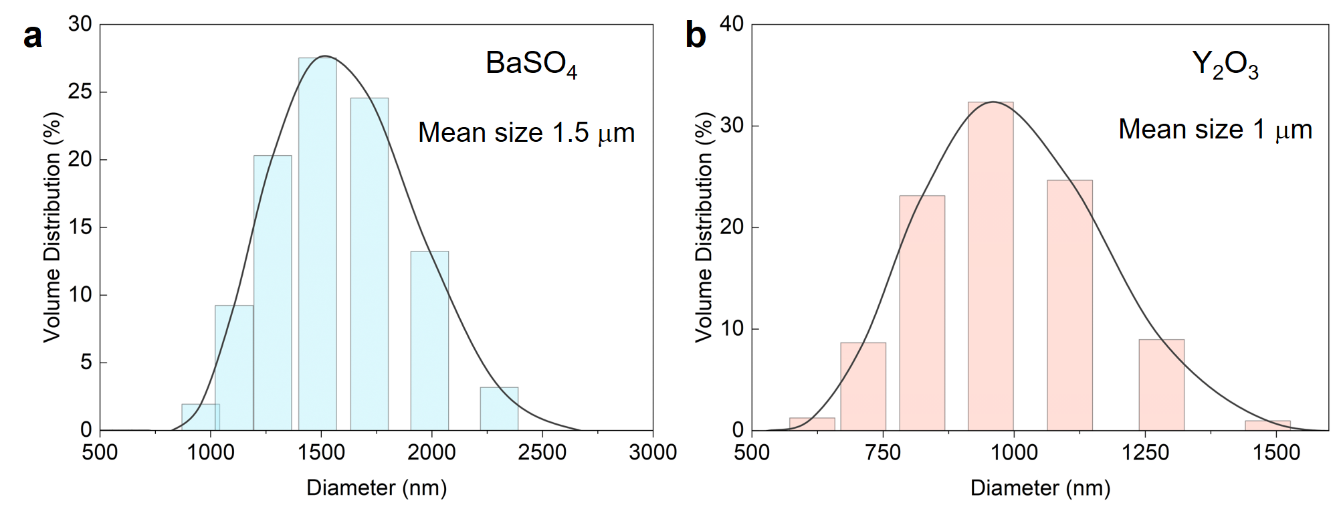 |
| --- |
| **Figure S3.** Particle size distributions of (a) BaSO_4_ and (b) Y_2_O_3_ nanoparticles in experiments. BaSO_4_ nanoparticle size spans from 900 nm to 2500 nm, with a mean size of 1500 nm. Y_2_O_3_ nanoparticle size spans from 600 nm to 1500 nm, with a mean size of 1000 nm. |

|  | |
| --- | --- |
|  | |
| 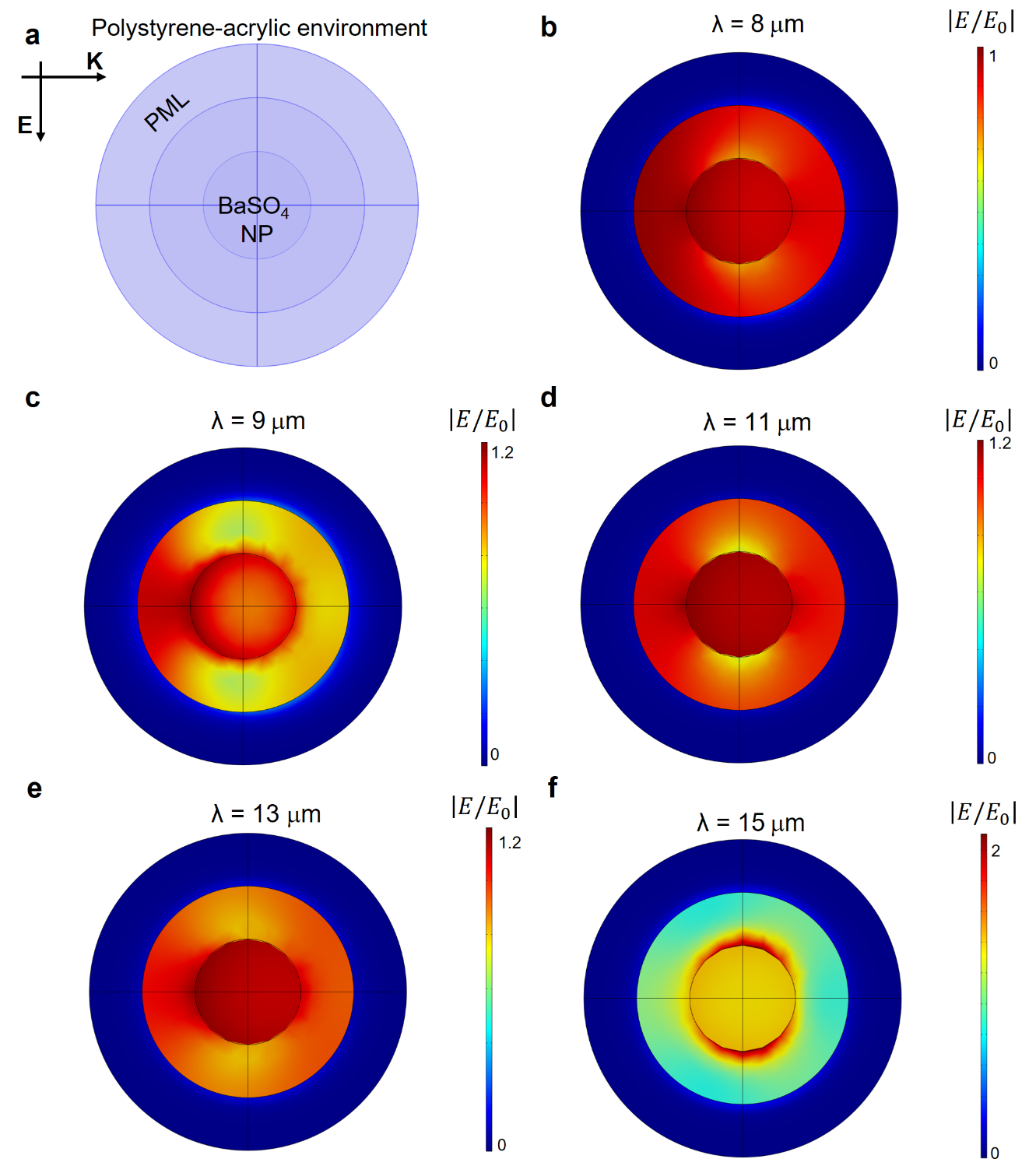 |  |
| **Figure S4.** COMSOL modeling for resonance behavior of the 1.5-µm BaSO_4_ nanoparticle in poly-styrene-acrylic matrix within MIR spectrum. (a) Schematic of simulation field with a plan wave incident and Perfect Matching Layer (PML) boundary condition. (b-f) Electric field distribution profiles of the 1.5-µm BaSO_4_ nanoparticle within 8-15 μm mid-infrared wavelength range. The resonance is prominently electric-dipolar. *E*_0_ indicates the incident electric field intensity. |  |

| 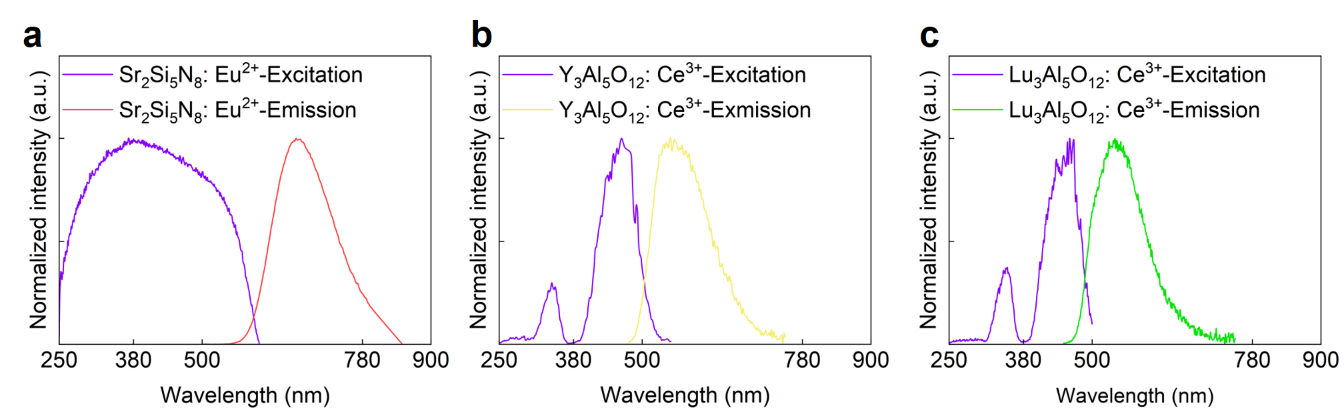 |
| --- |
| **Figure S5.** Excitation and emission spectra of (a) the red phosphor, (b) the yellow phosphor and (c) the green phosphor. |

| 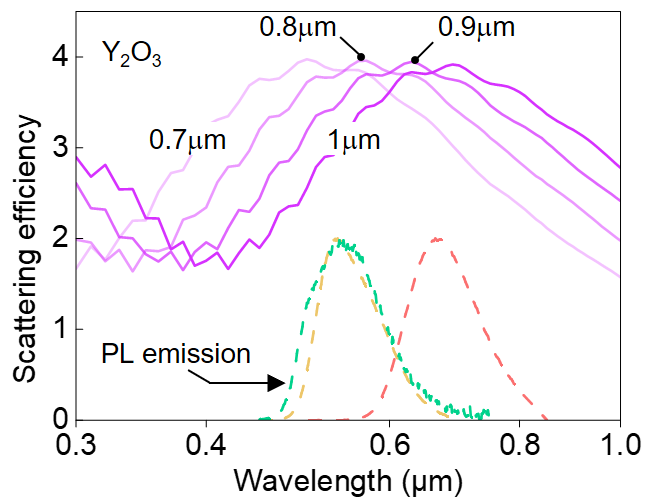 |
| --- |
| **Figure S6.** Scattering efficiencies of Y_2_O_3_ nanoparticles in the colored layer, which cover the emission peak wavelengths of three phosphors. |

| 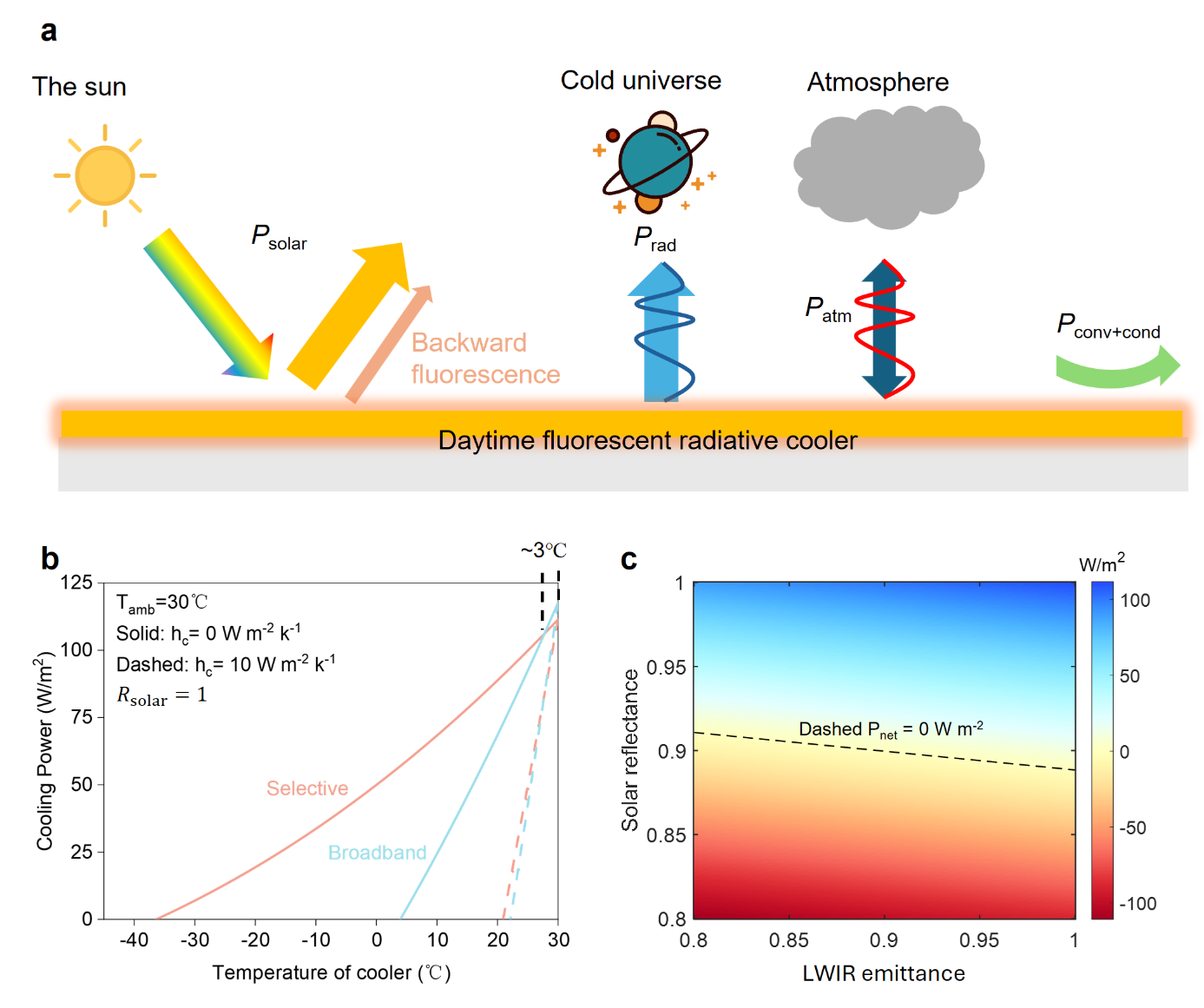 |
| --- |
| **Figure S7.** (a) Schematic of daytime heat transfer process of a fluorescent radiative cooler. (b) Relationship between cooling power and temperature of the ideal selective and broadband radiative coolers when ambient temperature is 30 ℃. (c) Effects of solar reflectance and LWIR emittance on net cooling power of an ideal broadband radiative cooler. |

| 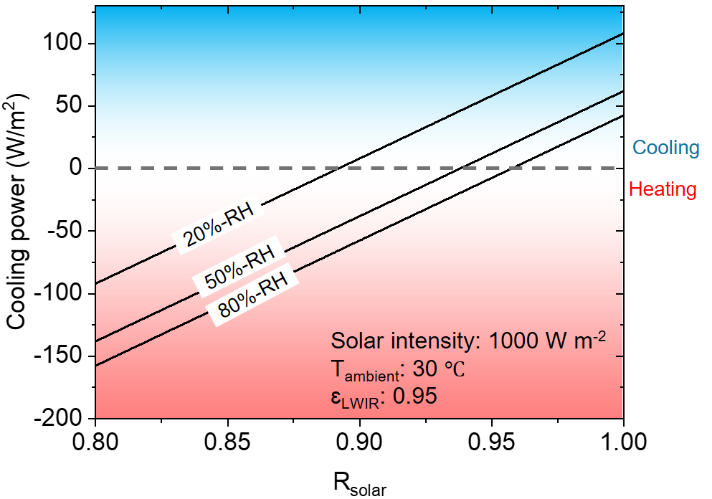 |
| --- |
| **Figure S8** Cooling power of radiative coolers with increasing solar reflectance ($R_{\mathrm{solar}}$) facing towards desert sky with low humidity and tropical sky with high humidity, where the corresponding atmosphere transparency spectra were obtained from MODTRAN modeling^[5]^ by setting Total Water Column as 1000, 3000 and 5000 atm-cm, which refer to 20%, 50% and 80% .relative humidity^[30]^, respectively. |

| 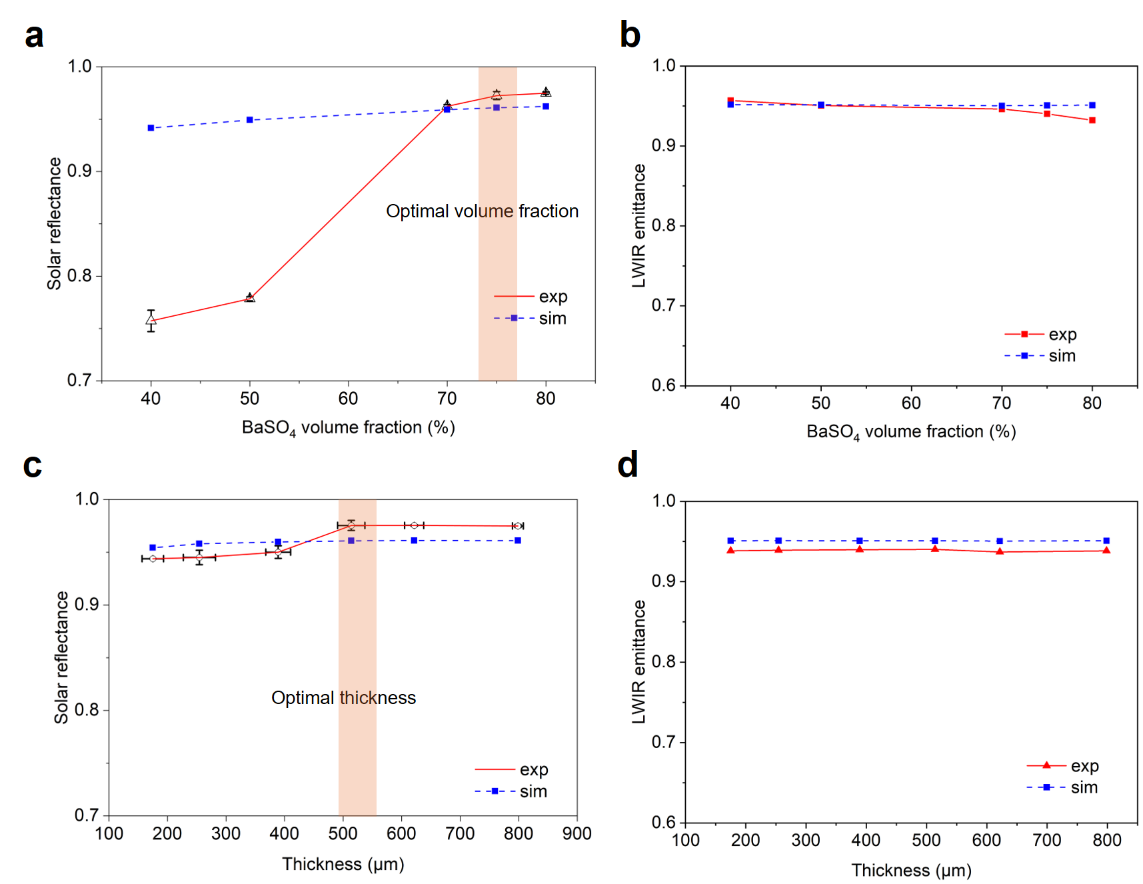 |
| --- |
| **Figure S9.** Experimental results of optimization for bottom layer. (a) Solar reflectance and (b) infrared emissivity of experimental groups for optimization of BaSO_4_ NPs’ volume fraction as thickness was fixed at ~500 μm. When volume fraction was 60%, the crack happens on the coating surface. Then, the optimal volume fraction of BaSO_4_ NPs was selected at 75%. (c) Solar reflectance and (d) Infrared emissivity of experimental groups for optimization of the bottom layer’s thickness as volume fraction of BaSO_4_ NPs was fixed at 75 vol.%. The optimal thickness of bottom was chosen as 500-600 μm. The optimal bottom coating exhibited a solar reflectance of 97.6% with a high LWIR emittance of 94.0%. |

| 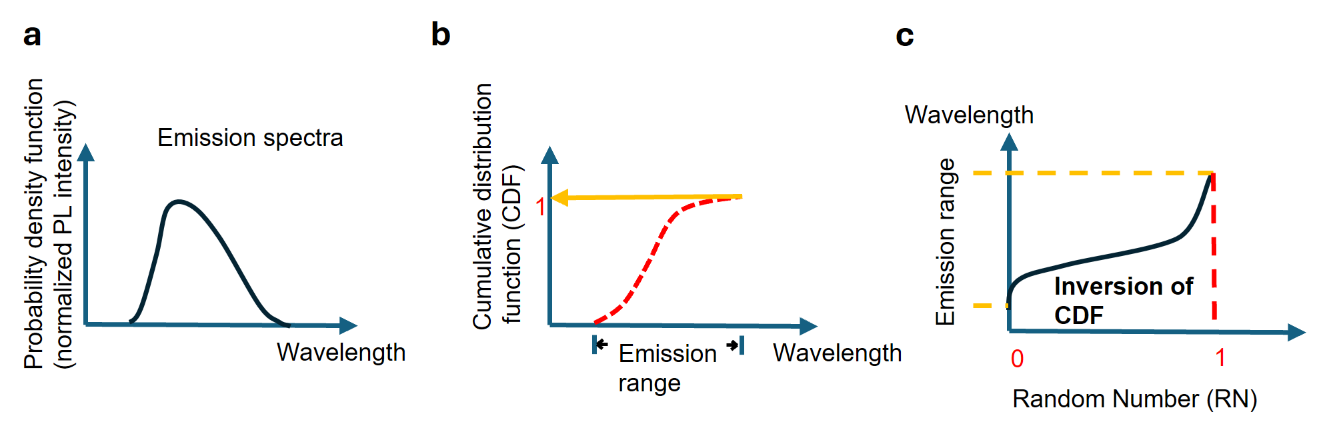 |
| --- |
| **Figure S10.** Schematic of reemitted fluorescence wavelength determination during ray-tracing process of modified Monte Carlo simulation. (a) Input probability density function (PDF), i.e., normalized PL emission spectra of phosphor. (b) Cumulative distribution function (CDF) versus emission wavelength range. (c) Determination of reemission wavelength by the inversion of CDF and random number from 0 to 1. |

| 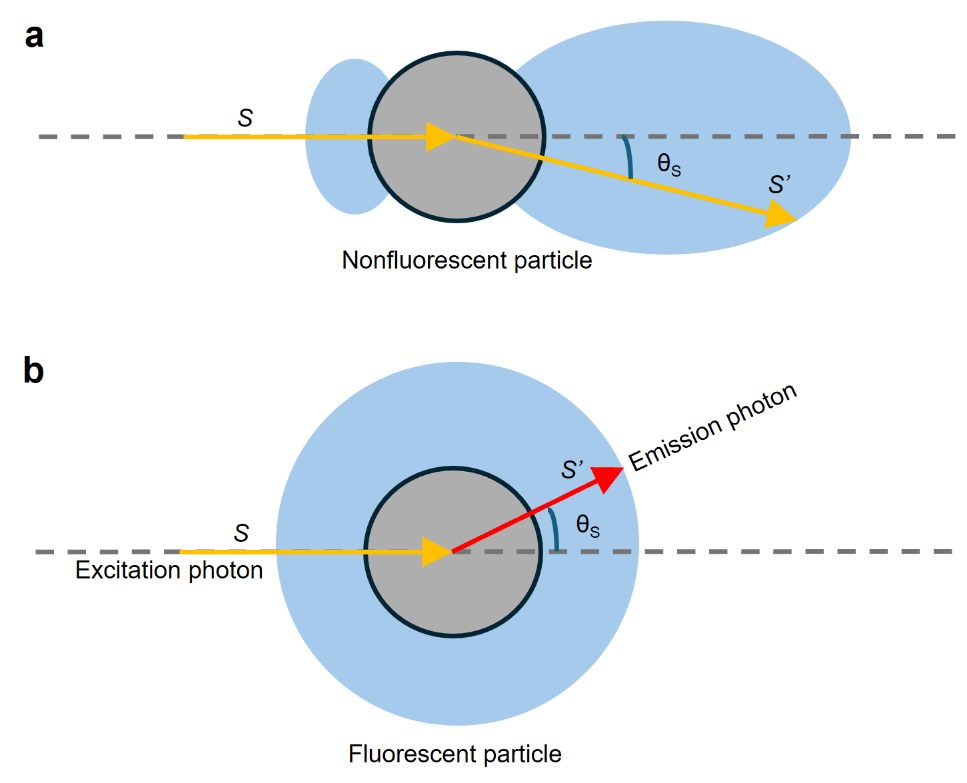 |
| --- |
| **Figure S11.** Schematic representations of ray bundle propagating from direction *S* is scattered into direction *S*’ by (a) the nonfluorescent particle and (b) the fluorescent particle. For the nonfluorescent particle, Mie scattering is prominently in the forward direction. In terms of the fluorescent particle, wavelength conversion and randomized scattering angle of re-emitted photons are considered for the appropriate fluorescence generation process. The blue shade is the schematic for the distribution of scattering rays. |

|  |
| --- |
| **Figure S12.** Flowchart of the modified Monte Carlo method for multilayer coatings with fluorescent pigments. |

| 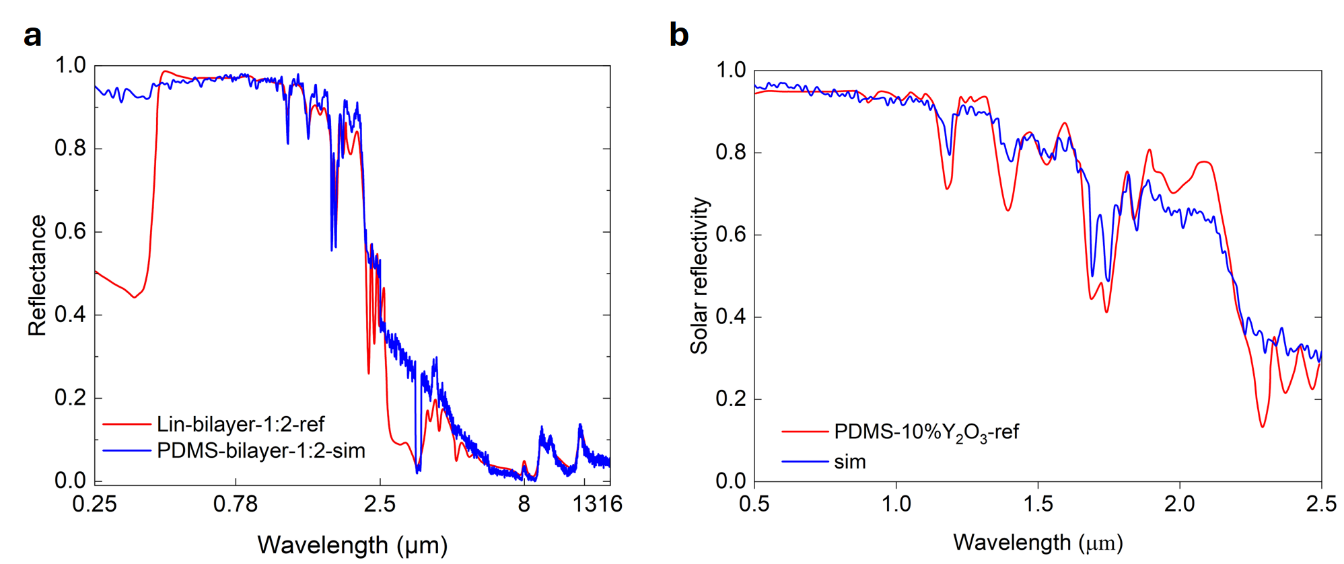 |
| --- |
| **Figure S13.** Validation of proposed Monte Carlo simulation via comparing with reported experiment results: (a) The bilayer PDMS-based coating in Lin et al.’s work^[16]^. (b**)** The monolayer PDMS-based coating with 10vol.% Y_2_O_3_ NPs^[17]^. These two cases verify the correctness of the MMC method handling multiplayer coating structure and the accuracy of the extracted refractive index of Y_2_O_3_. |

| 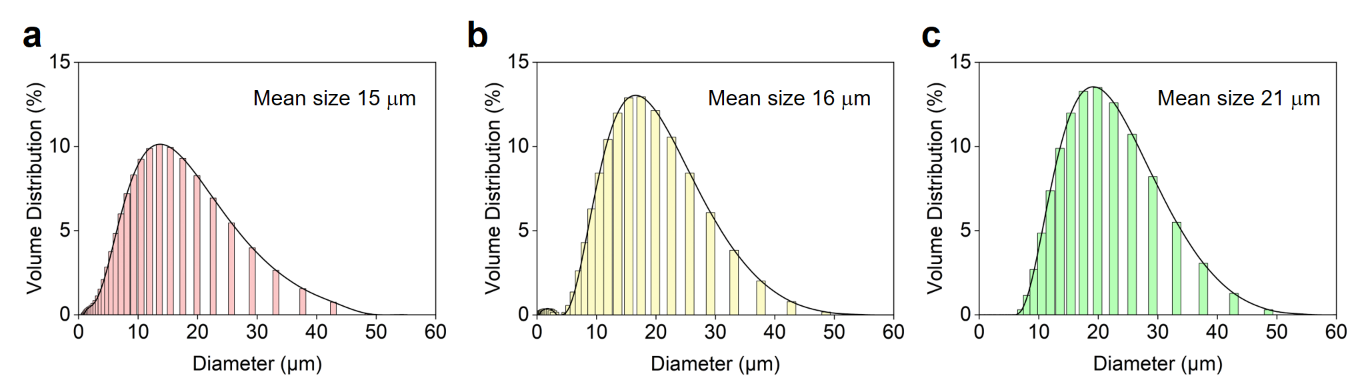 |
| --- |
| **Figure S14.** Particle size distributions of the (a) red phosphor, (b) yellow phosphor and (c) green phosphor. |

| 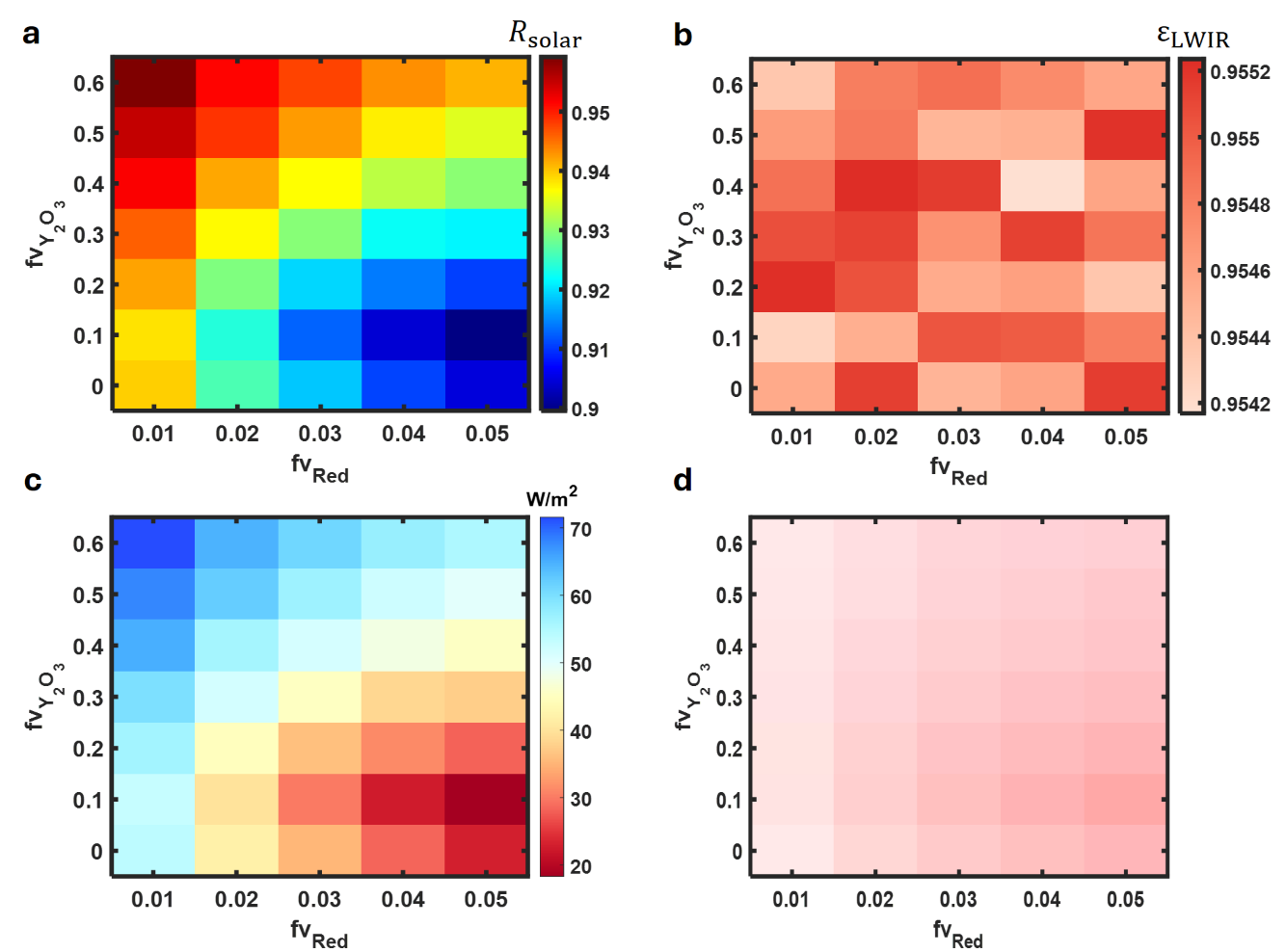 |
| --- |
| **Figure S15.** Effects of volume fractions of Y_2_O_3_ NPs and the red phosphors on (a) solar reflectance, (b) LWIR emittance, (c) cooling performance and (d) color appearance of the colored fluorescent tri-layer polymeric coating. The thickness of the colored layer was fixed at 20 µm. |

| 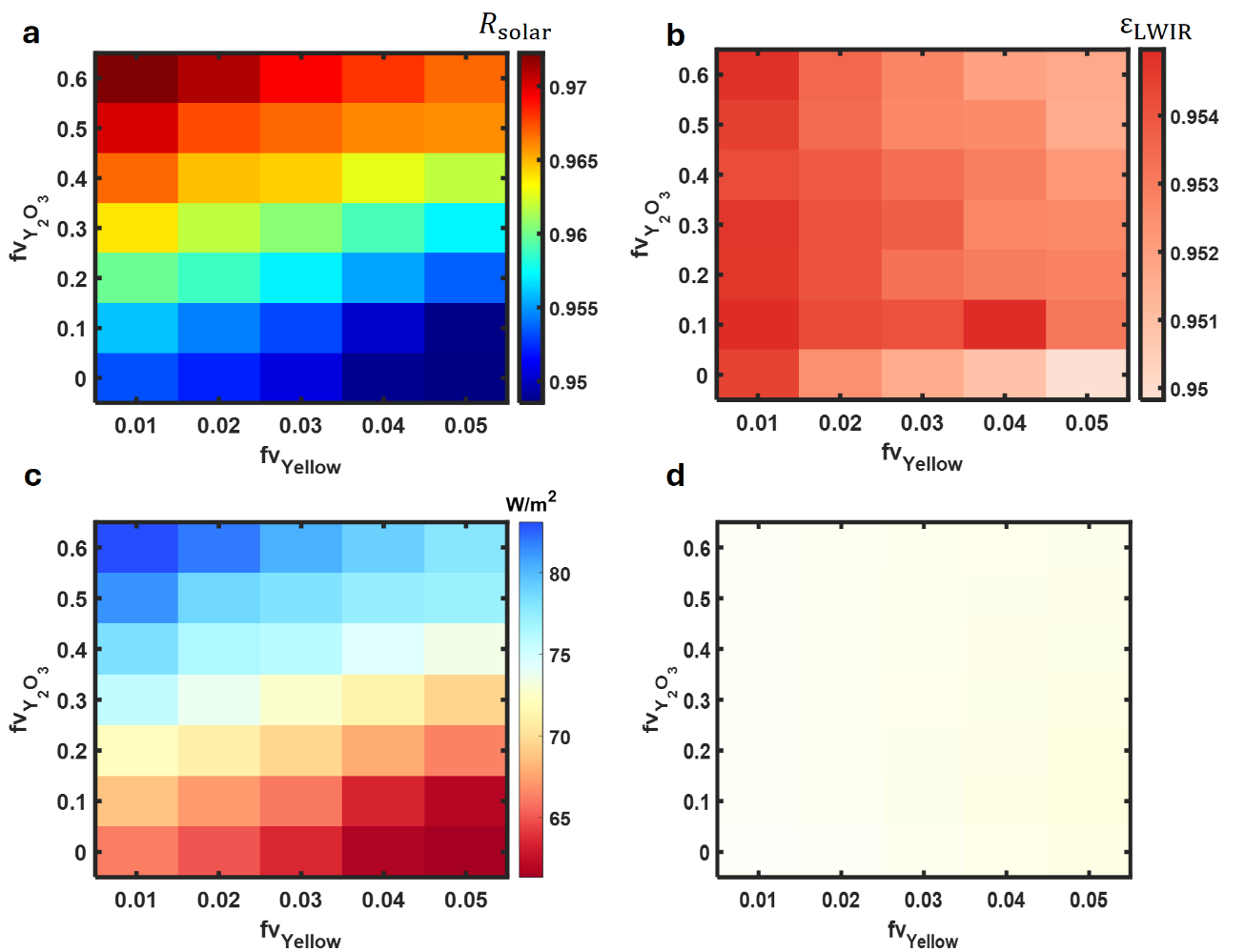 |
| --- |
| **Figure S16.** Effects of volume fractions of Y_2_O_3_ NPs and the yellow phosphors on (a) solar reflectance, (b) LWIR emittance, (c) cooling performance and (d) color appearance of the colored fluorescent tri-layer polymeric coating. The thickness of the colored layer was fixed at 20 µm. |

| 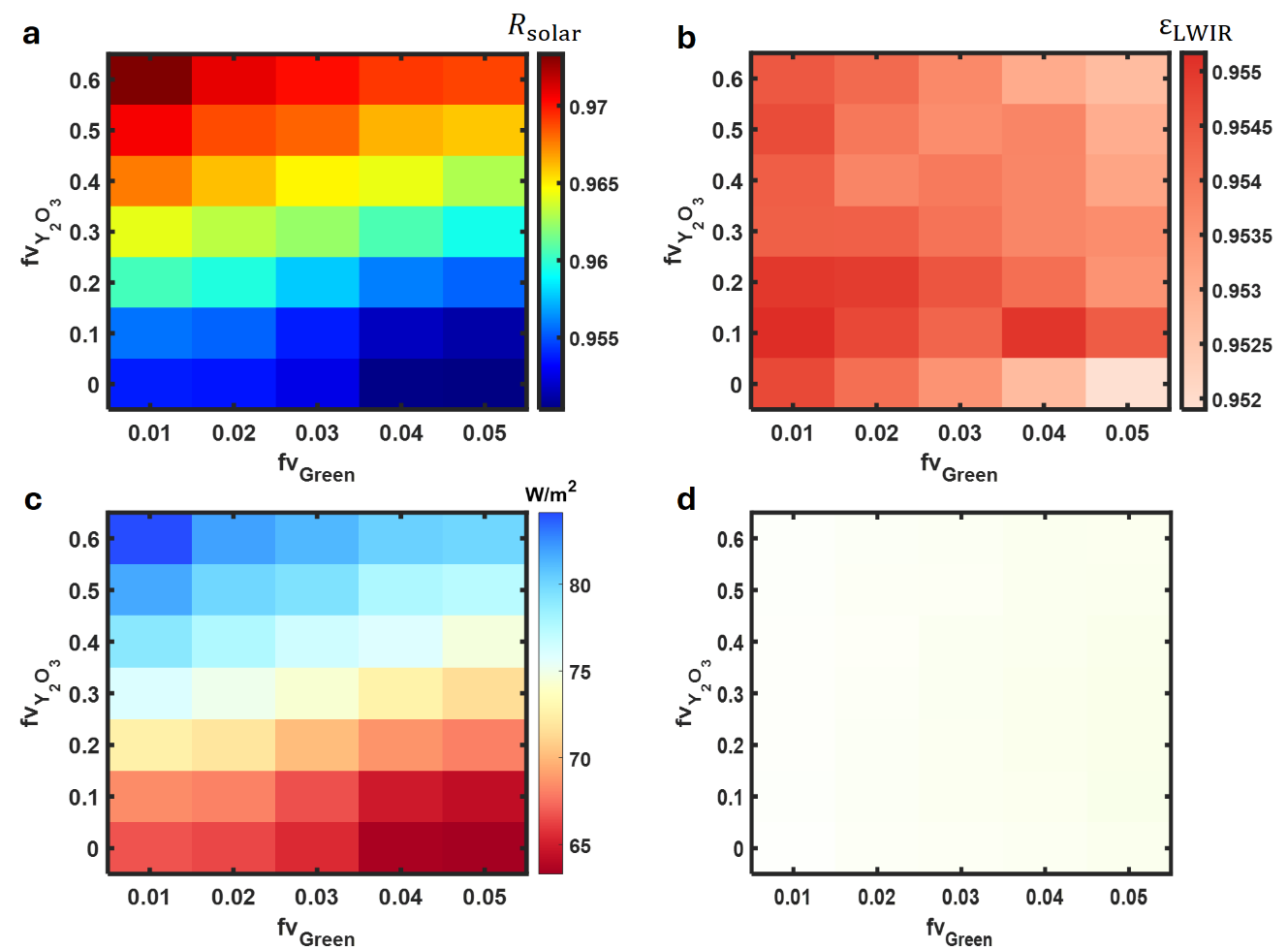 |
| --- |
| **Figure S17.** Effects of volume fractions of Y_2_O_3_ NPs and the green phosphors on (a) solar reflectance, (b) LWIR emittance, (c) cooling performance and (d) color appearance of the colored fluorescent tri-layer polymeric coating. Thickness of the colored layer was fixed at 20 µm. |

| 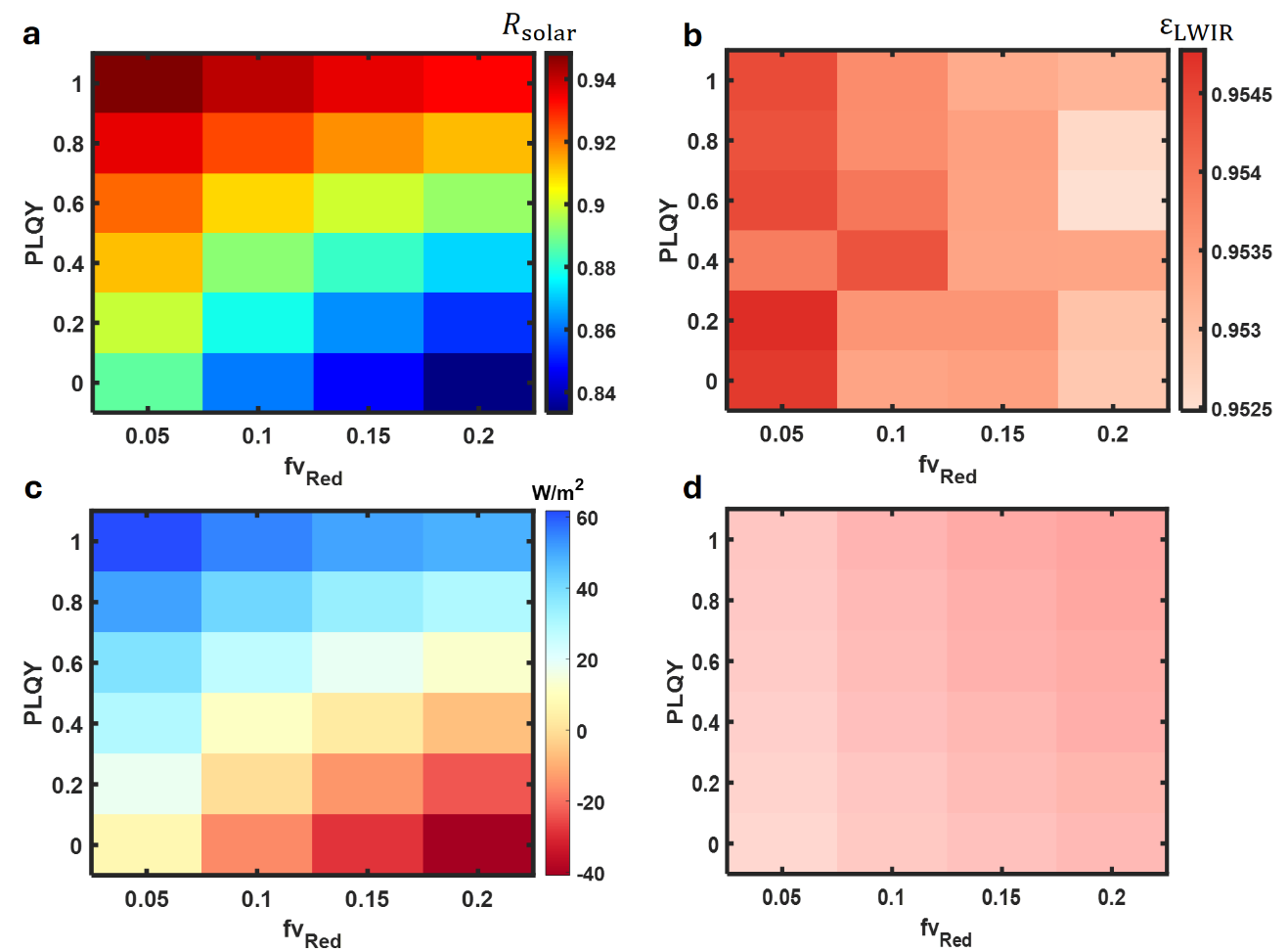 |
| --- |
| **Figure S18.** Effects of volume fractions of the red phosphor and its PLQY on (a) solar reflectance, (b) LWIR emittance, (c) cooling performance and (d) color appearance of the colored fluorescent tri-layer polymeric coating. The volume fraction of Y_2_O_3_ NPs and the colored layer’s thickness were fixed at 60% and 20 µm, respectively. |

| 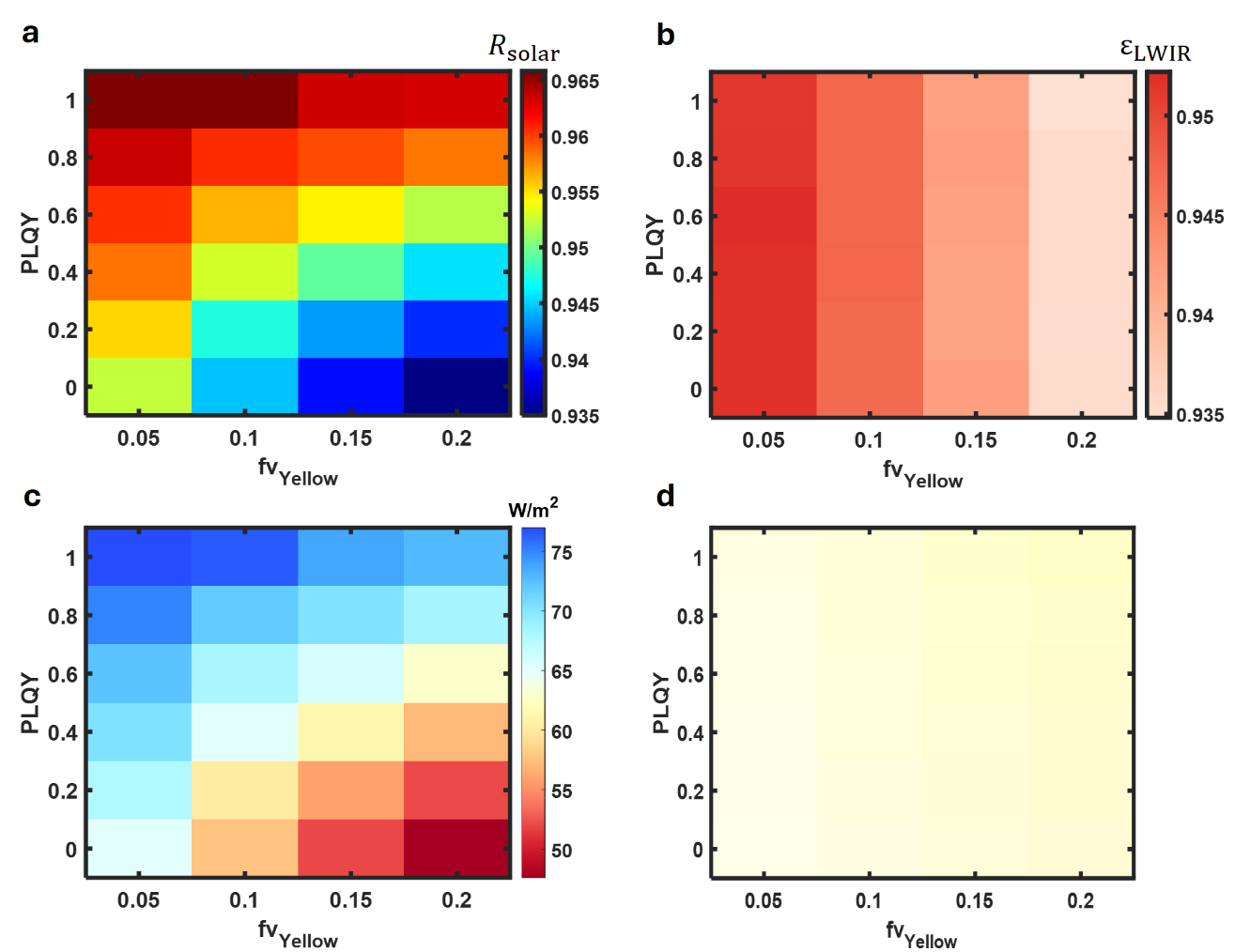 |
| --- |
| **Figure S19.** Effects of volume fractions of the yellow phosphor and its PLQY (a) solar reflectance, (b) LWIR emittance, (c) cooling performance and (d) color appearance of the colored fluorescent tri-layer polymeric coating. The volume fraction of Y_2_O_3_ NPs and the colored layer’s thickness were fixed at 40% and 20 µm, respectively. |

| 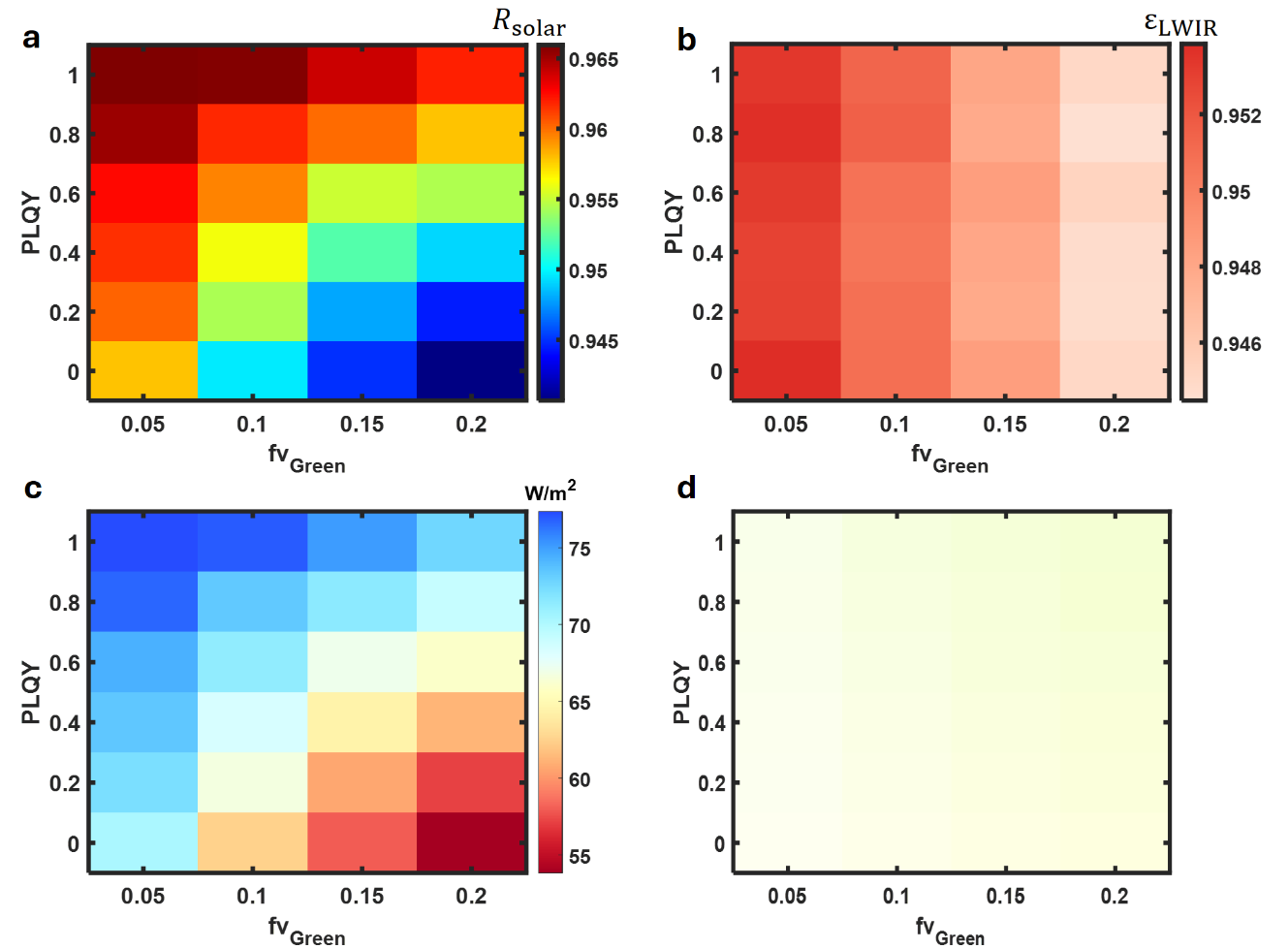 |
| --- |
| **Figure S20.** Effects of volume fractions of the green phosphor and its PLQY on (a) solar reflectance, (b) LWIR emittance, (c) cooling performance and (d) color appearance of the colored fluorescent tri-layer polymeric coating. The volume fraction of Y_2_O_3_ NPs and the colored layer’s thickness were fixed at 40% and 20 µm, respectively. |

| **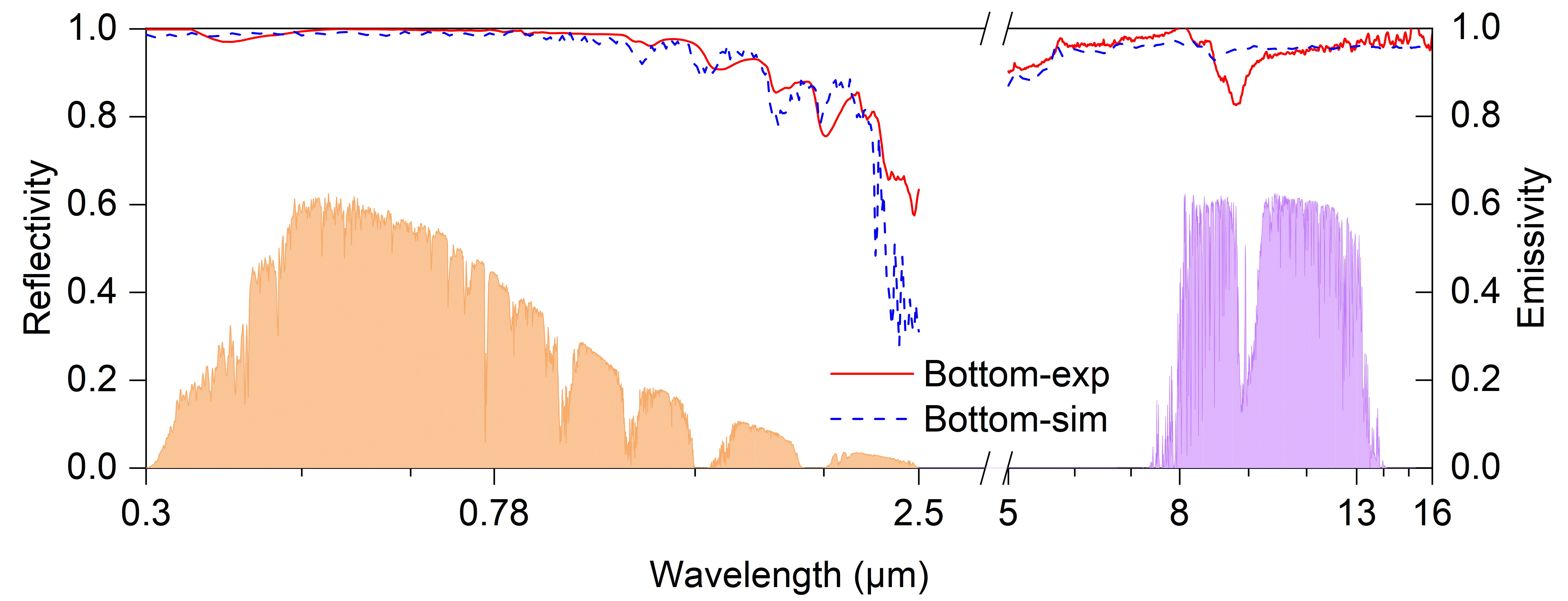** |
| --- |
| **Figure S21.** Solar reflectivity and infrared emissivity of the white bottom layer obtained by experiment tests and Monte Carlo simulation, respectively. The experimental solar reflectivity fits well with simulation result. The infrared emissivity difference within 9-11 μm wavelength can be ascribed to the deviation of extracted refractive index of BaSO_4_ from the reference. |

| 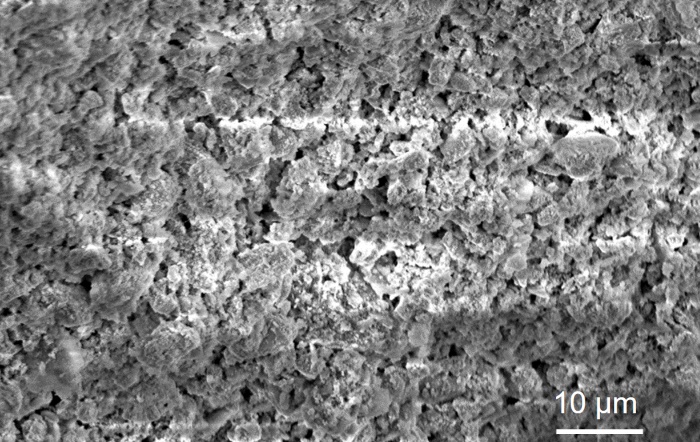 |
| --- |
| **Figure S22.** SEM images of cross-section of the white bottom layer. There are few air pores in the bottom layer, facilitating the backscattering of solar light. |

| 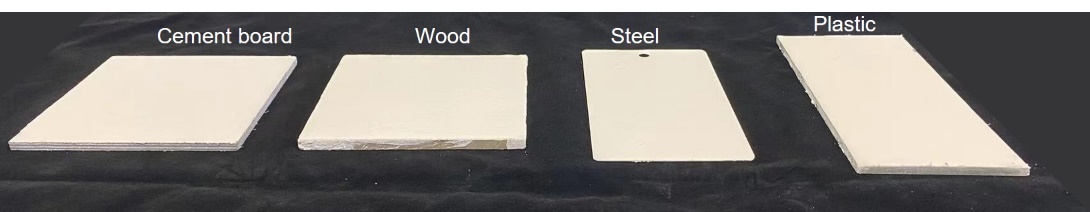 |
| --- |
| **Figure S23.** Fabrication of the white bottom layer on diverse substrates: cement board, wood, steel, and plastic (from left to right). |

| 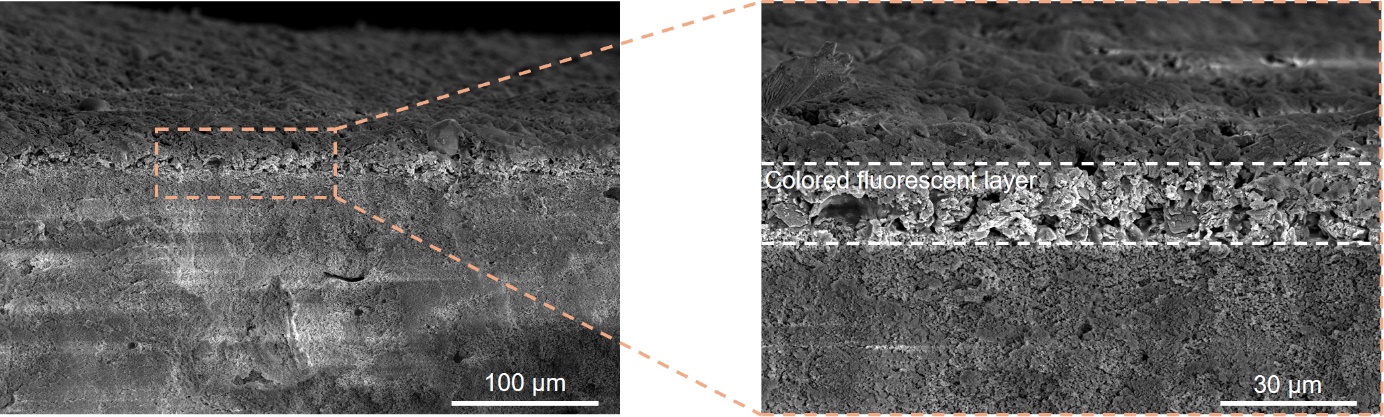 |
| --- |
| **Figure S24.** SEM images of enlarged cross-section of the colored layer in the tri-layer coating structure. The thickness of colored layer was about 20 µm. |

| 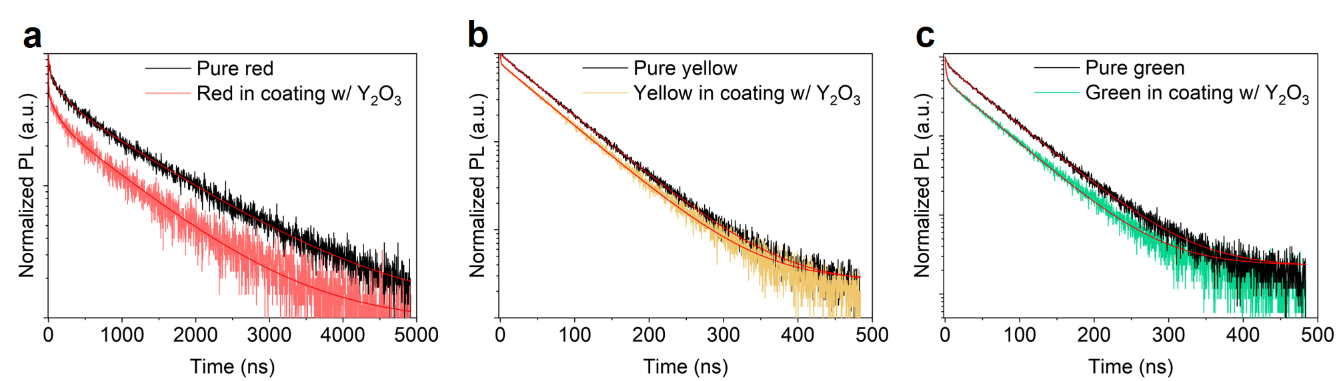 |
| --- |
| **Figure S25.** PL lifetime reduction of phosphors embedded in coating with Y_2_O_3_ NPs. Red lines in graphs indicates fitting lines. |

| 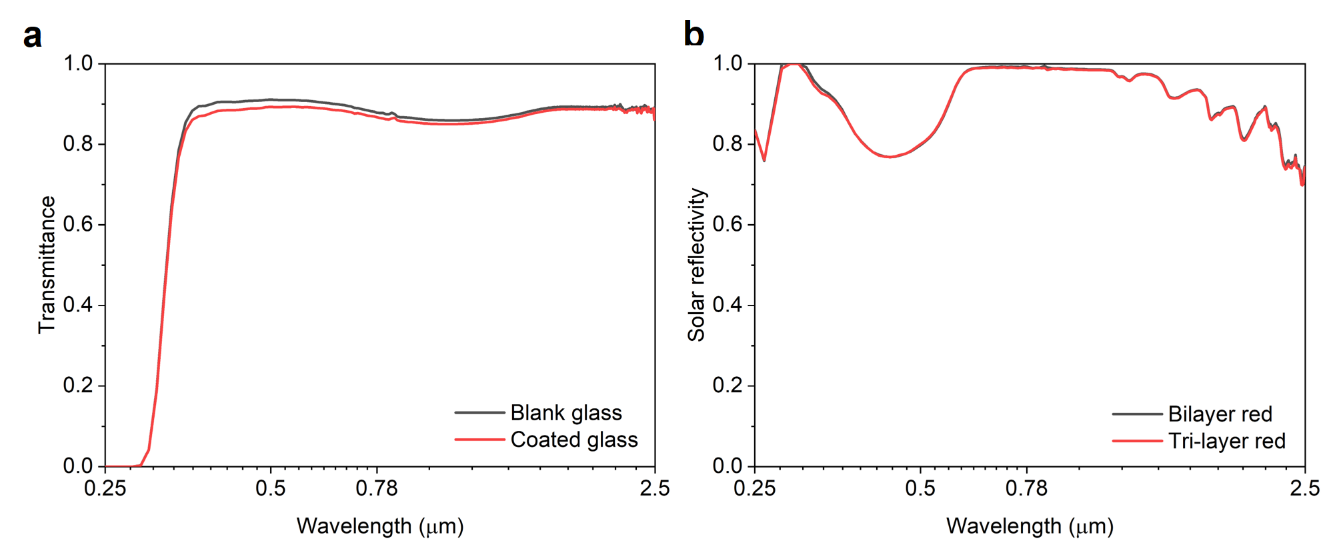 |
| --- |
| **Figure S26.** The effects of the uppermost silanized SiO_2_ NP layer on solar reflectivity. (a) Transmittance of the glass substrate before and after fabricating the uppermost silanized SiO_2_ NP layer (see Method). (b) “Solar reflectivity” of the red fluorescent coating before and after fabricating the topmost layer obtained by UV-visible-NIR spectrometric test. |

| **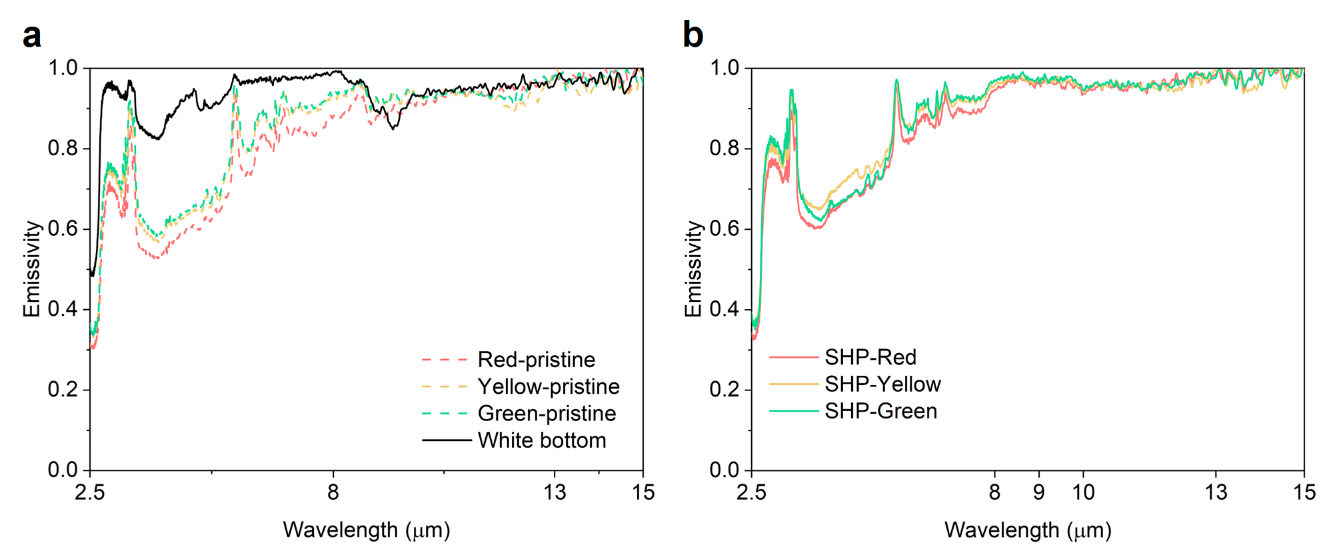** |
| --- |
| **Figure S27.** (a) Infrared emissivity spectra of white bottom, three colored bottom-middle bilayer coatings. (b) Infrared emissivity of self-cleaning colored fluorescent tri-layer polymeric coatings. |

| 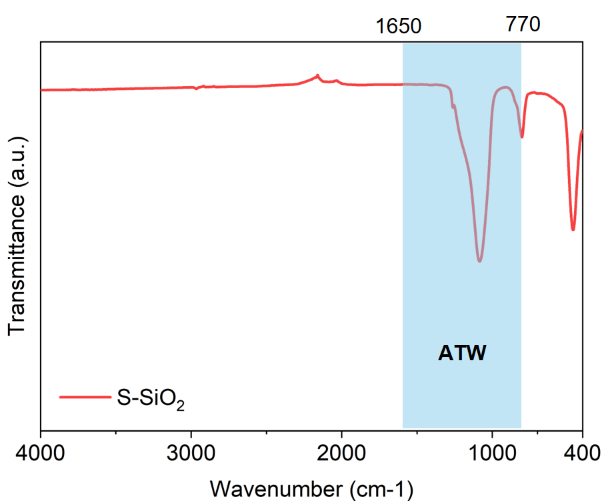 |
| --- |
| **Figure S28.** FT-IR spectra of silanized SiO_2_ nanoparticles. A strong infrared absorption was observed within the atmospheric transparent window (ATW) wavelength range. |

| 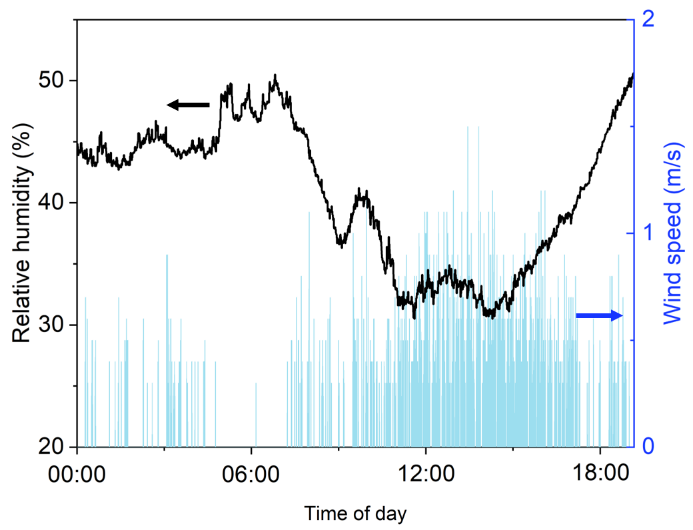 |
| --- |
| **Figure S29.** Meteorological data during outdoor field test (6 Jan 2025), including relative humidity (black) and wind speed (blue). |

| 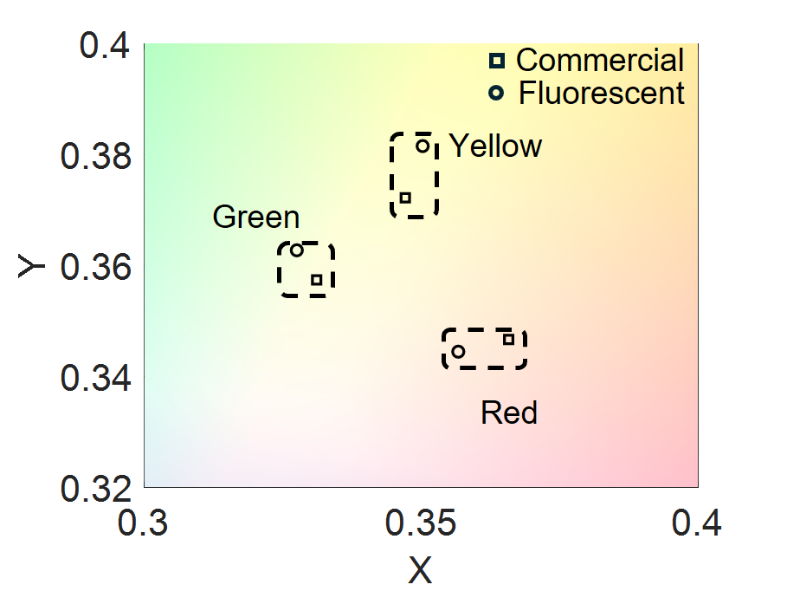 |
| --- |
| **Figure S30.** Comparison of chromaticity between fluorescent coatings and commercial coatings in the field test. |

| 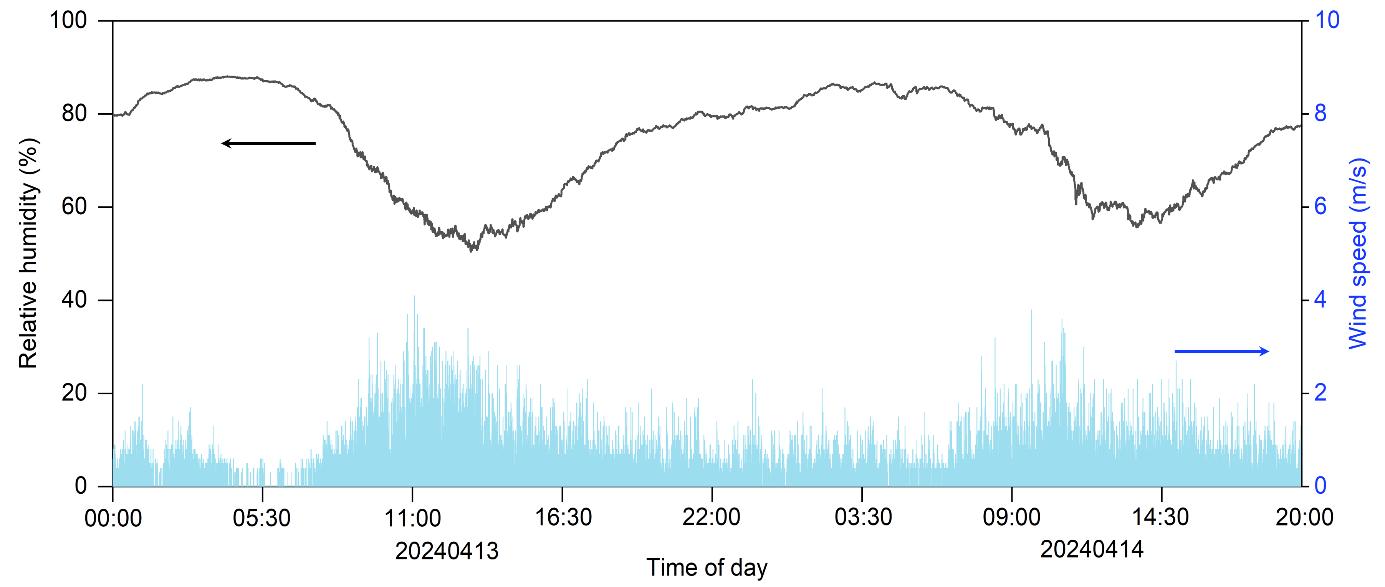 |
| --- |
| **Figure S31.** Meteorological data during outdoor field test (13-14 April 2024), including relative humidity (black) and wind speed (blue). |

| 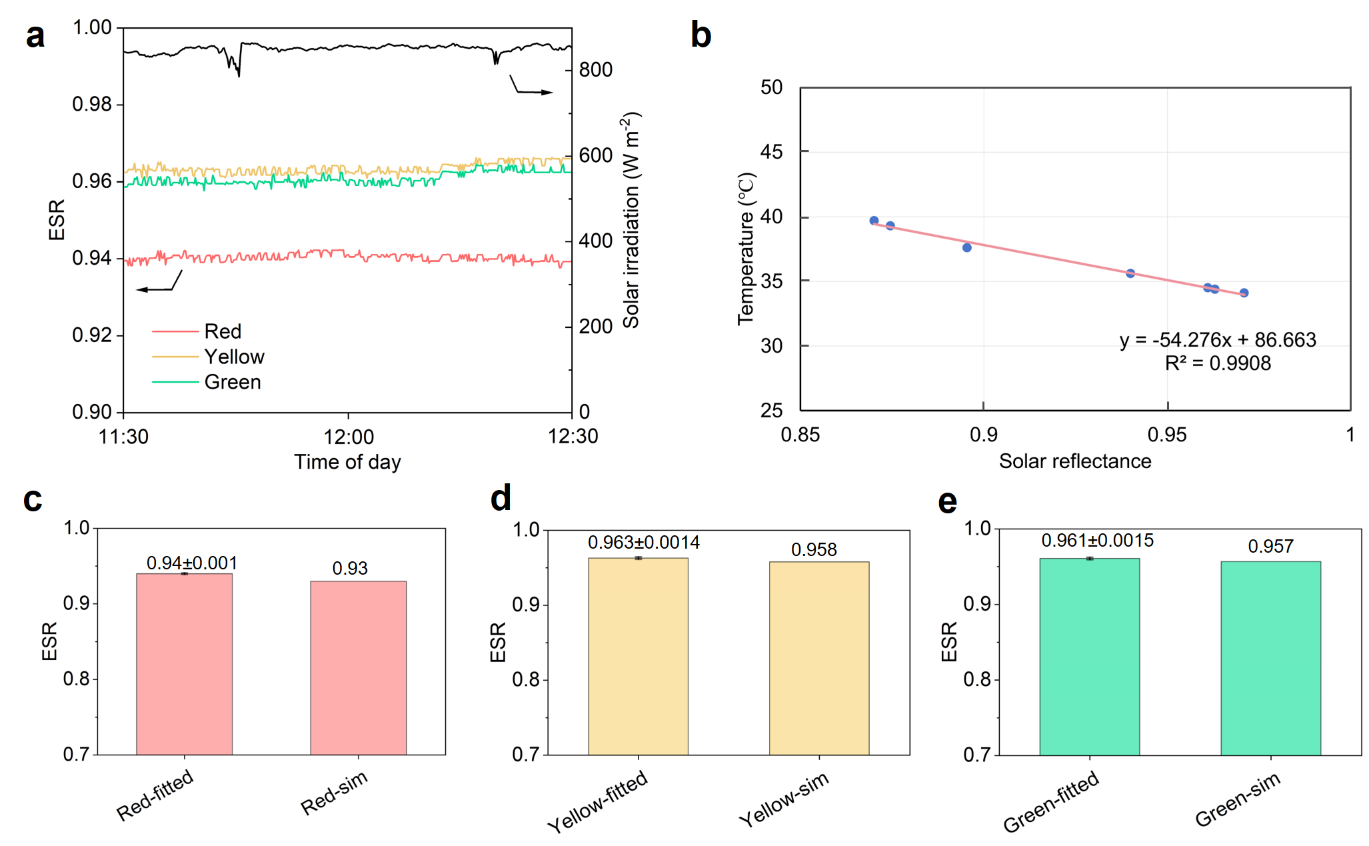 |
| --- |
| **Figure S32.** (a) Fitted effective solar reflectance of three colored fluorescent coating samples in the field test at noontime on 13 April 2024. (b) Fitting of the linear relationship between solar reflectance and temperature of coating samples in the field test (Taking temperature data at 12:00 as an example). The obtained fitting equation is $T= -54.276\times\mathrm{SR}+86.663$ with a high Goodness of Fit of about 0.99. (c-e) Comparison of effective solar reflectance results of the (c) red, (d) yellow, and (e) green fluorescent coatings, which were obtained by fitting method and MMC simulation. |

| 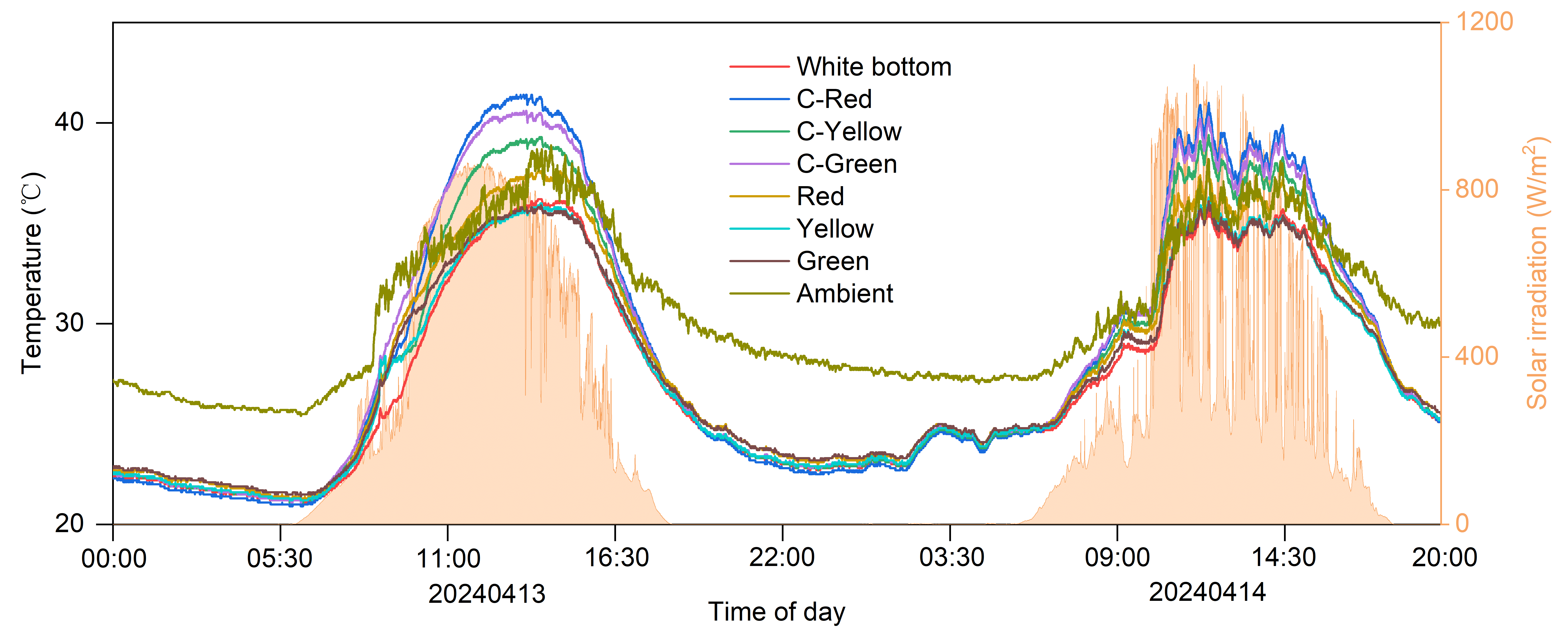 |
| --- |
| **Figure S33.** Field test results of all samples in Hong Kong during the outdoor field test (13-14 April 2024). |

| 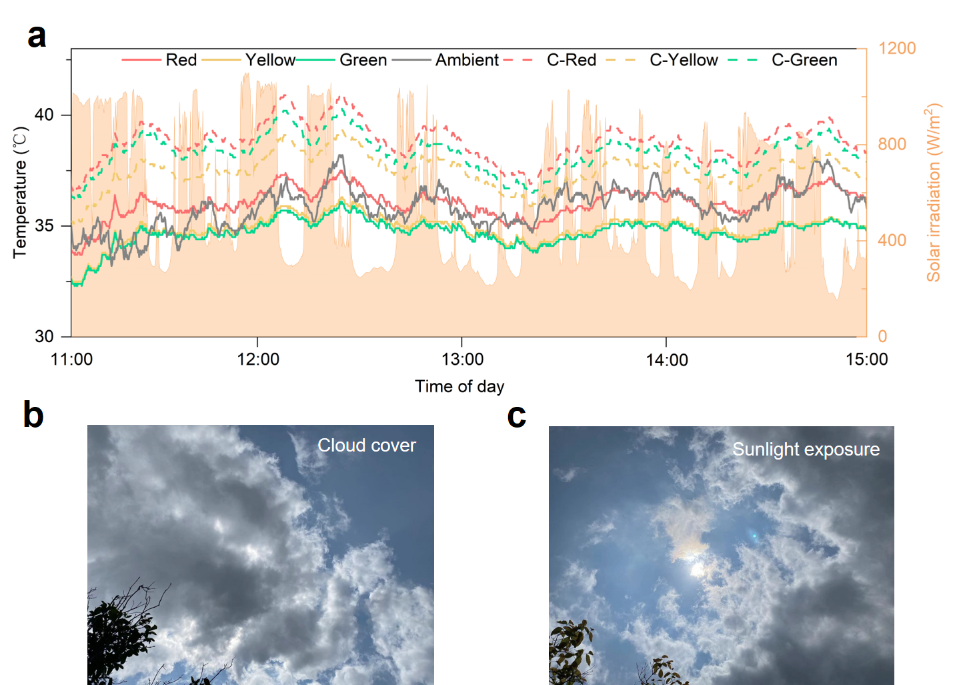 |
| --- |
| **Figure S34.** (a) Temperature of coating samples on a cloudy day (14 April 2024). Photographs of the sky at the time that (b) cloud cover and (c) sunlight exposure during outdoor cooling test. |

| 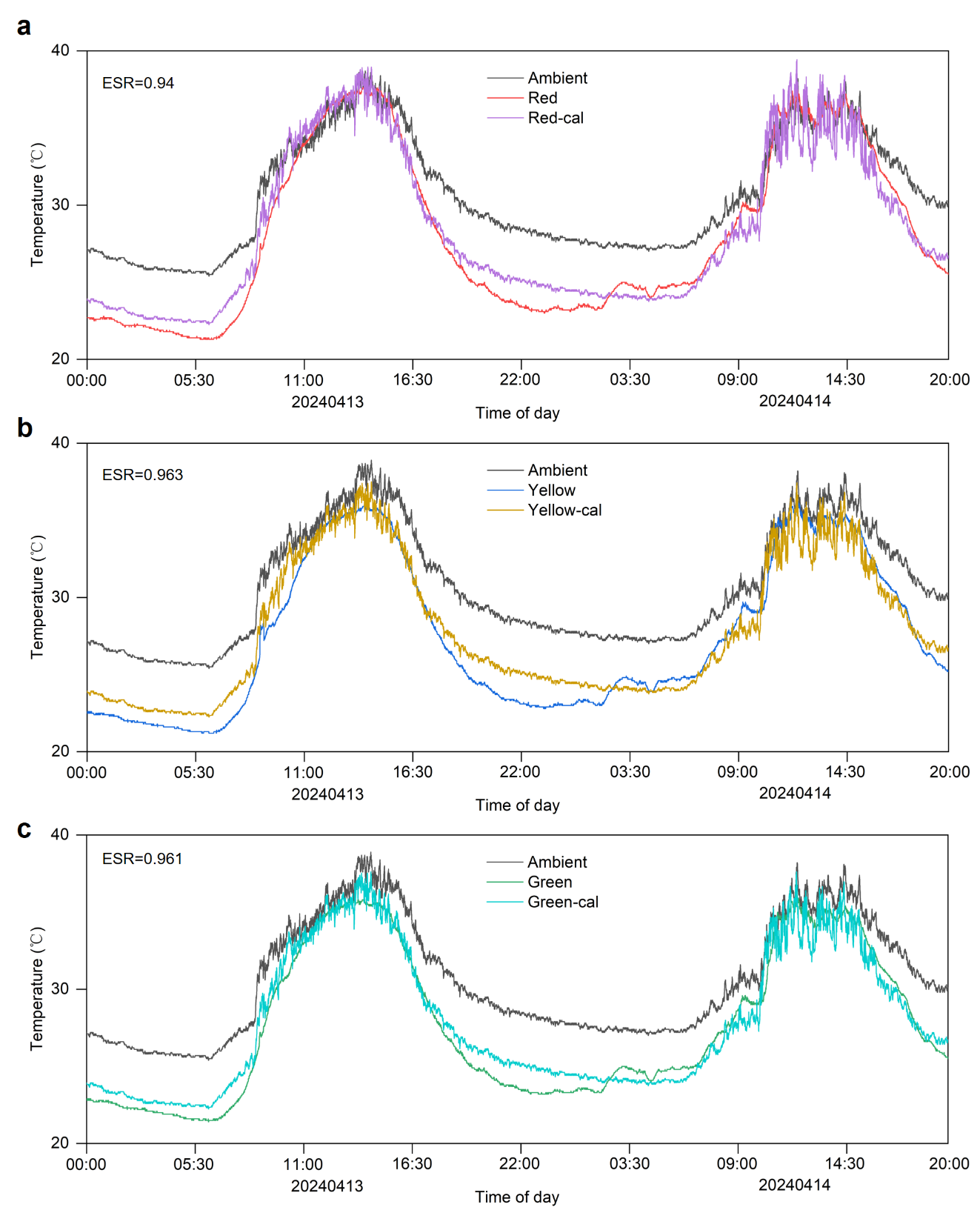 |
| --- |
| **Figure S35.** Comparison between real-time recorded temperature (13-14 April 2024) and calculated temperature through the heat transfer model. |

| 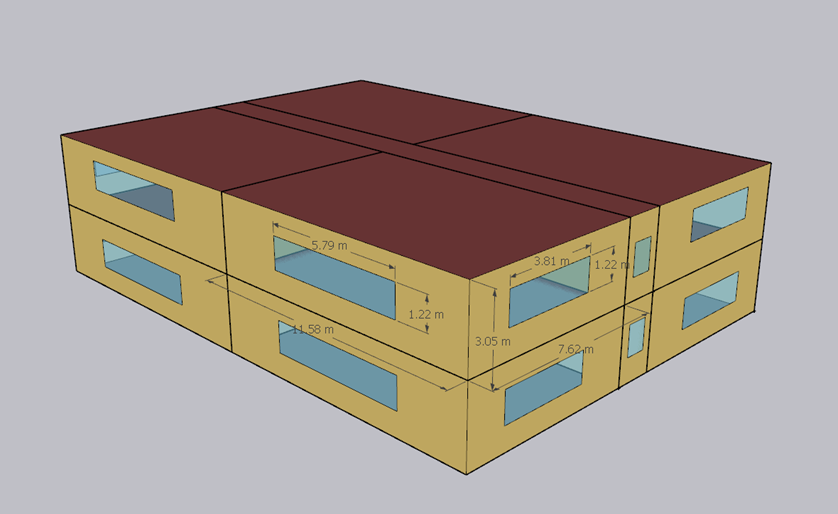 |
| --- |
| **Figure S36.** Mid-rise apartment model used in the EnergyPlus simulation. The building structure information and model definition are listed in **Tables S**5-7. |

| 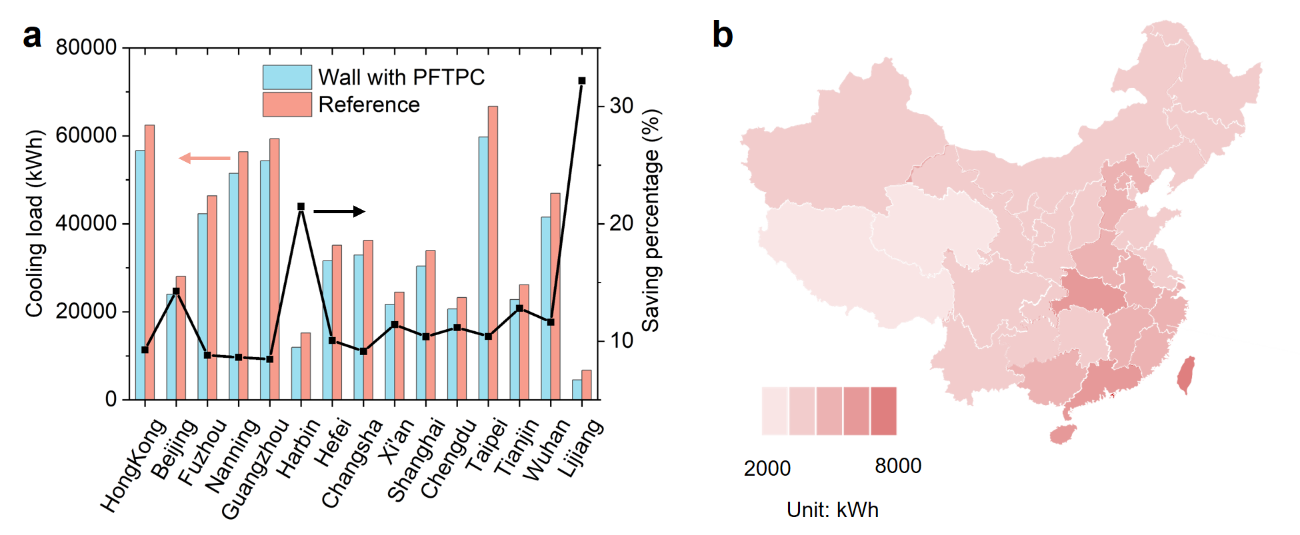 |
| --- |
| **Figure S37.** (a) Cooling loads and energy saving percentages of the PFTPC as a colored cooling wall coating in primary cities in China. (b) Cooling energy saving results mapping across China. |

| **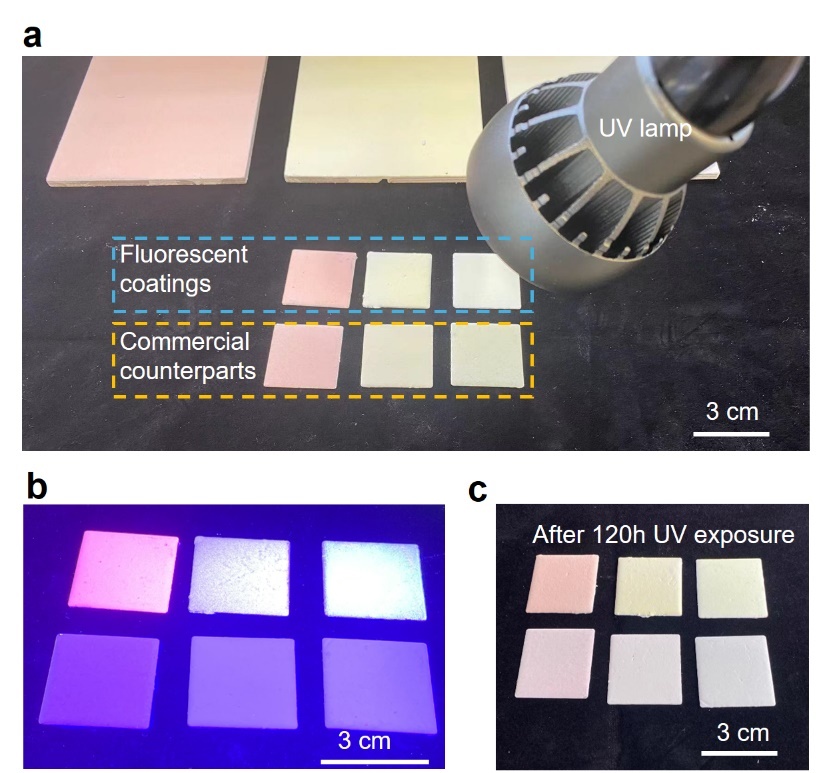** |
| --- |
| **Figure S38. UV exposure test setup.** (a) Photographs of fluorescent coatings and commercial counterparts where UV irradiation wavelength and power was 405 nm and ~175 W/m^2^, respectively. (b) Photographs of fluorescent coatings and commercial counterparts under UV illumination. (c) Photographs of coatings after UV exposure for 120 hours. |

| 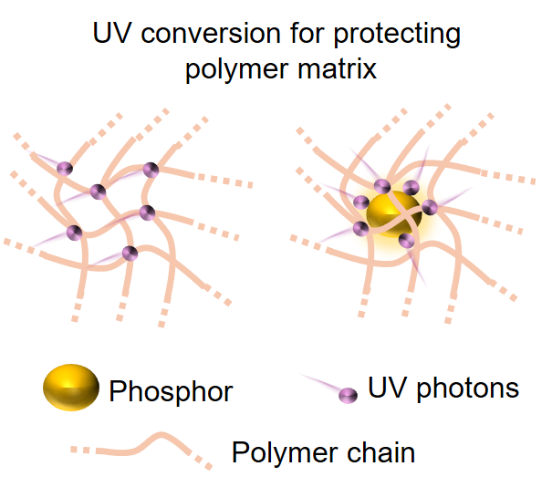 |
| --- |
| **Figure S39.** Schematic of protection role of phosphors on organic polymer matrix through UV conversion. |

| 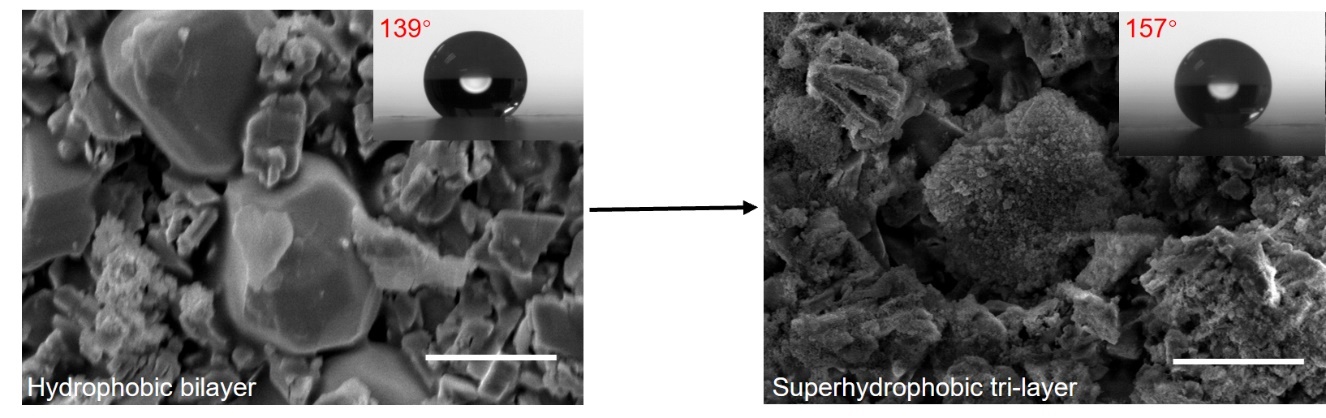 |
| --- |
| **Figure S40.** Change of surface morphology and surface wetting behavior after fabricating the uppermost silanized SiO_2_ NP layer. |

| 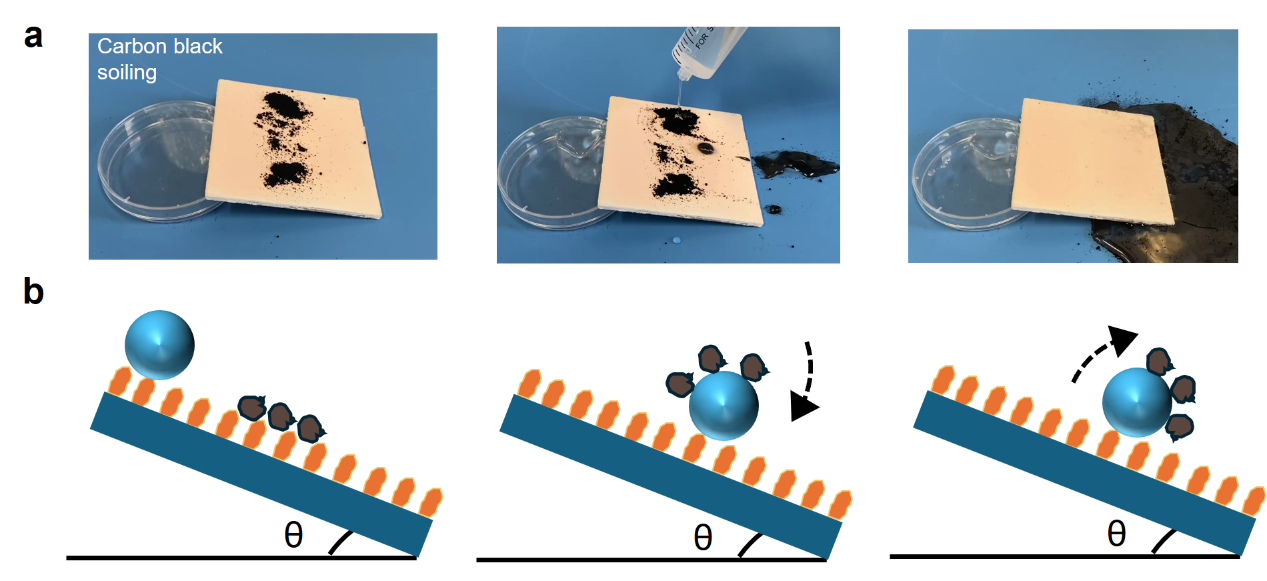 |
| --- |
| **Figure S41.** (a) Photographs of water droplet driven cleaning effect against the soiling of Carbon black particles. (b) Illustration of the PFTPC showing self-cleaning properties. |

| 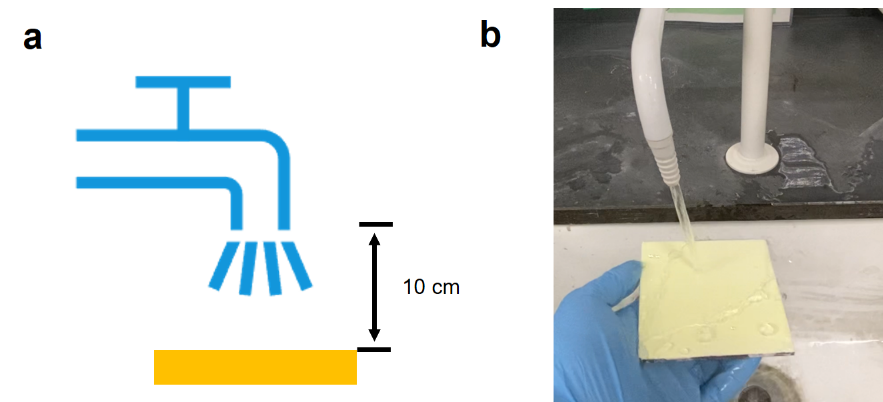 |
| --- |
| **Figure S42.** (a) Schematic of high-speed water impact test. (b) Photograph of high-speed water impact test. |

| 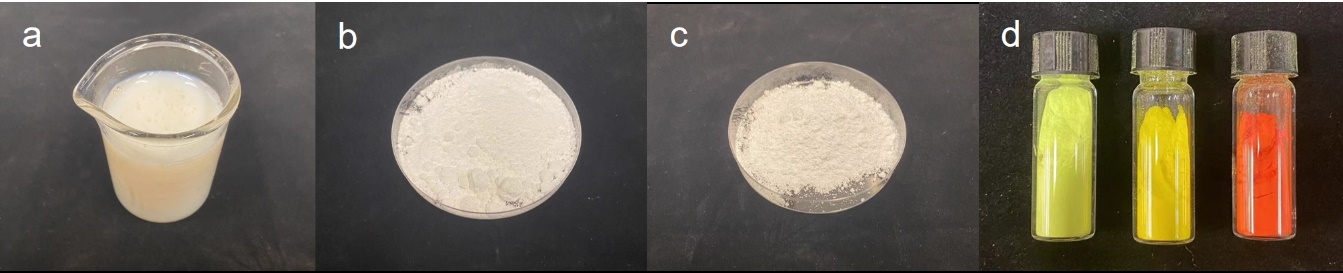 |
| --- |
| **Figure S43.** Optical images of primary raw materials including (a) poly-styrene-acrylic emulsion, (b) BaSO_4_ nanoparticles, (c) Y_2_O_3_ nanoparticles and (d) phosphors. |

| 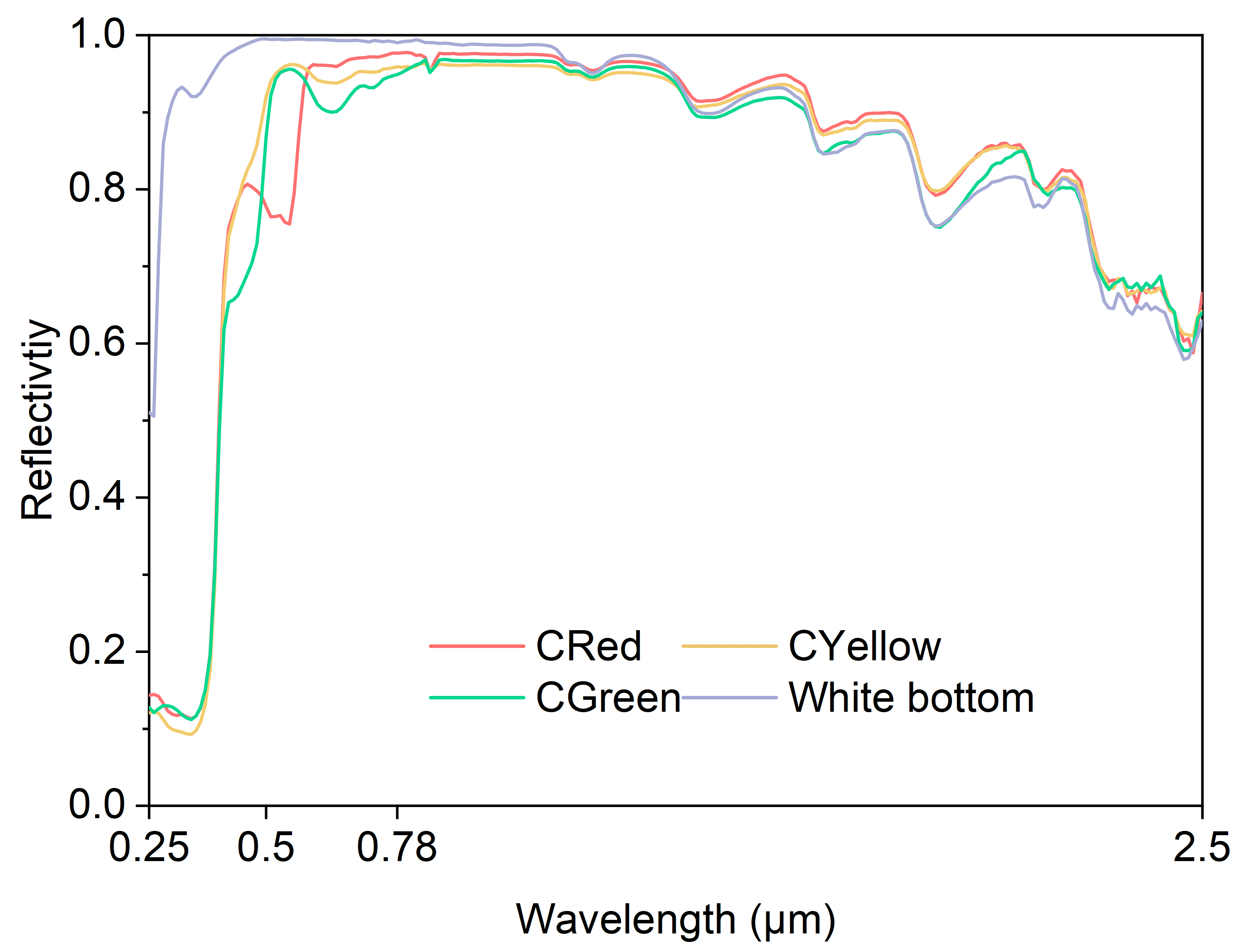 |
| --- |
| **Figure S44.** Solar reflectivity spectra of bilayer commercial colored coating counterparts and white bottom coating in field test. |

| 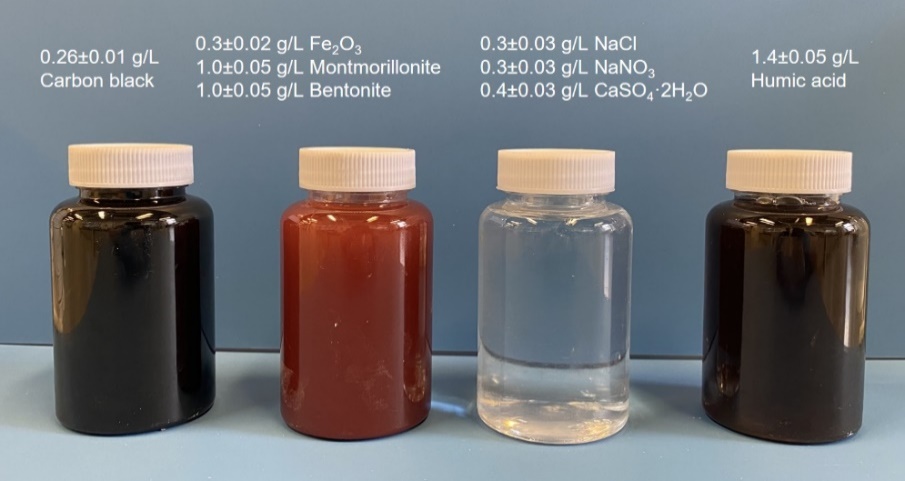 |
| --- |
| **Figure S45.** Photographs of soiling dusts used in soiling resistance test. |

# S3. Supporting Tables

**Table S1.** Fitted radiative lifetime ($\tau_{1})$ and ($\tau_{2}$) and non-radiative lifetime ($\tau_{3}$) for pure red phosphors and phosphors in cooling coating with Y_2_O_3_ NPs.

|  | Pure red phosphor | Red phosphor in Y_2_O_3_ coating |
| --- | --- | --- |
| $\boldsymbol{\tau}_{\boldsymbol{1}}$ | 181.2 | 116.7 |
| $\boldsymbol{\tau}_{\boldsymbol{2}}$ | 1205.9 | 961.3 |
| $\boldsymbol{\tau}_{\boldsymbol{3}}$ | 15.8 | 1.0 |

**Table S2.** Fitted radiative lifetime ($\tau_{1})$ and non-radiative lifetime ($\tau_{2}$) for pure yellow phosphors and phosphors in cooling coating with Y_2_O_3_ NPs.

|  | Pure yellow phosphor | Yellow phosphor in Y_2_O_3_ coating |
| --- | --- | --- |
| $\boldsymbol{\tau}_{\boldsymbol{1}}$ | 63.2 | 60.7 |
| $\boldsymbol{\tau}_{\boldsymbol{2}}$ | 13.7 | 0.4 |

**Table S3.** Fitted radiative lifetime ($\tau_{1})$ and non-radiative lifetime ($\tau_{2}$) for pure green phosphors and phosphors in cooling coating with Y_2_O_3_ NPs.

|  | Pure green phosphor | Green phosphor in Y_2_O_3_ coating |
| --- | --- | --- |
| $\boldsymbol{\tau}_{\boldsymbol{1}}$ | 56.7 | 51.8 |
| $\boldsymbol{\tau}_{\boldsymbol{2}}$ | 3.6 | 0.6 |

**Table S4.** Infrared emittance of bottom, bottom-middle bilayer coatings and self-cleaning tri-layer coating.

| $\boldsymbol{\varepsilon}_{\mathbf{LWIR}}\boldsymbol{/}\boldsymbol{\varepsilon}_{\boldsymbol{2.5-15.4\mu m}}$ | Green | Yellow | Red |
| --- | --- | --- | --- |
| Bottom | 0.943/0.953 | | |
| Bottom-middle bilayer | 0.938/0.923 | 0.931/0.914 | 0.924/0.901 |
| Self-cleaning tri-layer | 0.968/0.948 | 0.963/0.945 | 0.961/0.939 |

**Table S5.** Envelop structures and their thermophysical properties.

| Envelope | Materials (from outside to inside) | Thickness (mm) | Thermal Conductivity (Wm^-1^ K^-1^) | Density (kg/m^3^) | Specific heat (J kg^-1^K^-1^) |
| --- | --- | --- | --- | --- | --- |
| Roof | Roof Membrane | 9.5 | 0.16 | 1121.29 | 1460 |
|  | Concrete Slab | 101.6 | 0.53 | 1280 | 840 |
|  | Gypsum | 12.7 | 0.16 | 784.9 | 830 |
| Walls | Stucco | 25.3 | 0.6918 | 1858 | 837 |
|  | Gypsum Board | 19 | 0.16 | 800 | 1090 |
|  | Wall Insulation | 33.7 | 0.04322 | 91 | 837 |
|  | Gypsum Board | 19 | 0.16 | 800 | 1090 |
| Window | Clear glass | 6 | 0.9 | - | - |

**Table S6.** Building model definitions.

| Items | Values |
| --- | --- |
| Running period | 6:00 – 18:00, 1^st^ Jan to 31^st^ Dec |
| A/C system | Ideal air load system |
| Cooling setpoint | 26 ℃ |
| People | 0.056511 people/m^2^ |
| Electric Lighting | 10.656271 W/m^2^ |
| Electric load | 7.642376 W/m^2^ |
| Infiltration | 0.0003026 m^3^/s/floor area(m^2^) |

**Table S7.** Optical properties of developed PFTPC and reference standard roof and wall in building modeling.

|  | PFTPC | Reference standard roof | Reference standard wall |
| --- | --- | --- | --- |
| Solar absorptance | 0.037 | 0.5 | 0.5 |
| Visible absorptance | 0.037 | 0.5 | 0.5 |
| Thermal absorptance | 0.96 | 0.92 | 0.92 |

**Table S8.** Preliminary cost estimation of the colored fluorescent tri-layer polymeric coating.

| Layer (thickness) | Raw materials items | Price ^a^ ($/kg) | Weight (g) | Fabrication area (m^2^) | Cost of each layer per unit area ($/m^2^) | Total cost of the tri-layer coating per unit area ($/m^2^) |
| --- | --- | --- | --- | --- | --- | --- |
| Bottom (~500µm) | Polystyrene acrylic emulsion | ~1.2 | 10 | ~0.038 | ~1.0 | ~6.7 |
|  | BaSO_4_ nanoparticle | ~0.4 | 65 |  |  |  |
| Middle ^b^ (~20µm) | Polystyrene acrylic emulsion | ~1.2 | 10 | ~0.476 | ~5.64 |  |
|  | Y_2_O_3_ nanoparticle | ~5 | 19 |  |  |  |
|  | Phosphor | ~600 | 4.3 |  |  |  |
| Uppermost  (~1µm) | Silanized SiO_2_ nanoparticle | ~42 | 0.4 | ~3 | ~0.038 |  |
|  | Ethanol | ~7 | 10 |  |  |  |
|  | Tetraethyl orthosilicate | ~34 | 0.8 |  |  |  |

^a^ Price was directly referred to lab-scale purchase cost.

^b^ The mixing proportion here was referred to the yellow coating for example.

**Table S9.** Comparison of the colored fluorescent tri-layer polymeric coatings and recently reported colored radiative coolers.

| References | Structures | Colors | Optical performance* | | Self-cleaning | Fabrication | Scalability and applicability | Cost |
| --- | --- | --- | --- | --- | --- | --- | --- | --- |
|  |  |  | $R_{\mathrm{solar}}$ | $\varepsilon_{\mathrm{LWIR}}$ |  |  |  |  |
| S. Fan, 2018, *Nat. commun*^[31]^ | Multilayer photonic structure | Pink | 0.33 | ~0.8 | No | Electron beam deposition | Low | High |
| Y. Song, 2018, *Adv. Opt. Mat* ^[32]^ | Multilayer resonant structure | Cyan  Magenta  Yellow | NA* | 0.8 | No | Electron beam deposition | Low | High |
| S. Kim, 2022, *Nano Lett* ^[33]^ | Thin-film stacking photonic structure | Yellow  Magenta  Cyan  Green | 0.46  0.69  0.61  0.57 | 0.92  0.93  0.92  0.92 | No | Electron beam deposition | Low | High |
| T. Li, 2022, *Adv. Sci* ^[34]^ | Colored cooling cellulose-based films | Blue  Green  Red | >0.90 (0.4-2 µm) | ~0.9 | No | Blading; ambient drying | Moderate | Moderate |
| R. Ma, 2022, Nano Lett ^[35]^ | Interferometric retroreflection-based assembly structure | Blue  Green  Red | NA | ~0.96 | No | Unidirectional rubbing and spinning; heating | Low | Moderate |
| Y. Yang, 2020, *Sci. Adv* ^[36]^ | Bilayer polymer with dyes | Blue  Yellow  Red | 0.4  0.72  0.61 | 0.95 | No | Brushing; ambient drying | High | Low |
| Z. Wang, 2023, *Science* ^[37]^ | Porous Al_2_O_3_ ceramic with dyes | Yellow  Red  Green | 0.919  0.907  0.912 | 0.96 | Yes | Molding; 1000 ℃-sintering | Moderate | Moderate |
| J. Zhu, 2023, *Sci. Bull.* ^[38]^ | Bilayer films with perovskite quantum dot | Yellow  Green  Red | >0.90 | 0.95 | No | Electricspinning and spraying; ambient drying | High | Moderate |
| S. Oh, 2021, *Nano Energy* ^[39]^ | Colored emitters with perovskite nanocrystal | Green  Red | 0.81  0.78 | ~0.98 | No | Sputtering, blading and spinning | Low | Moderate |
| D. Lei, 2024,  *Adv. Opt. Mater* ^[40]^ | Bilayer polymer with phosphors | Red  Yellow  Green | 0.93  0.94  0.936 | >0.90 | No | Spraying | High | Low |
| D. Lei, 2024, *J. Mater. Chem. A* ^[41]^ | Bilayer polymer with phosphors | Light-blue | 0.94 | 0.889 | No | Spraying | High | Low |
| M. Li,  2025, *Matter* ^[42]^ | Self-assembled polystyrene nanoparticles on porous polymer base | Green | 0.934 | 0.923 | No | Blading | Moderate | Moderate |
| Z. Mao, 2025, *ACS Nano* ^[43]^ | Porous fiber-based fabric with carbon dots | Red  Yellow  Blue  Green | NA | NA | No | Wet-spinning, freeze-drying, coating, and weaving procedures | High | Moderate |
| R. Xiong, 2025, *ACS Nano* ^[44]^ | Cellulose nanocrystals on cellulose nanofiber-based textile | Blue  Green  Red | 0.92 | >0.90 | No | Ambient drying and self-assembly | Moderate | Moderate |
| This work | Colored fluorescent tri-layer polymeric coatings | Red  Yellow  Green | 0.94  0.963  0.961 | >0.96 | Yes | Spraying or brushing; ambient drying | High | Low |

*Data was directly extracted from reported values in the corresponding references.

*“NA” refers to no reported data.

**Table S10.** Density information of primary raw materials in experiments.

| Materials | Density (g/cm^3^) |
| --- | --- |
| Polystyrene acrylic (solid) | 1.05 |
| BaSO_4_ nanoparticle | 4.5 |
| Y_2_O_3_ nanoparticle | 5.01 |
| Red phosphor (Sr_2_Si_5_N_8_:Eu^2+^) | 3.6 |
| Yellow phosphor (Y_3_Al_5_O_12_:Ce^3+^) | 4.57 |
| Green phosphor (Lu_3_Al_5_O_12_:Ce^3+^) | 6.73 |

**Table S11.** Solar reflectance of coatings of the reference group in the field test.

|  | UV | Vis | NIR | Total |
| --- | --- | --- | --- | --- |
| C-Red | 0.133 | 0.864 | 0.9476 | 0.8701 |
| C-Green | 0.1346 | 0.864 | 0.9343 | 0.8745 |
| C-Yellow | 0.1151 | 0.905 | 0.935 | 0.8954 |
| White bottom | 0.929 | 0.9914 | 0.9496 | 0.9711 |

**Table S12.** Composition of soiling mixture for diverse climates.

|  | Dusts | POMs | Soots | Salts |
| --- | --- | --- | --- | --- |
| Hot and Dry | 79% | 0% | 1% | 20% |
| Hot and Humid | 16% | 69% | 8% | 7% |
| Moderate | 61% | 0% | 8% | 31% |

# S4. Supporting Movies

**Movie S1.** Antifouling properties of the PFTPC.

**Movie S2.** Self-cleaning effect of the PFTPC on Carbon Black soiling agent.

**Movie S3.** Soiling resistance of the PFTPC based on the standard of ASTM D7897-18.

**Movie S4.** High-speed water jets impact the PFTPC.

# S5. References

[1] M. I. Mishchenko, P. Yang, *J. Quant. Spectrosc. Radiat. Transfer* **2018**, 205, 241.

[2] X. Ma, Y. Fu, A. Portniagin, N. Yang, D. J. Liu, A. L. Rogach, J. G. Dai, D. Y. Lei, *J. Mater. Chem. A* **2022**, 10, 19635.

[3] M. Chen, D. Pang, J. Mandal, X. Chen, H. Yan, Y. He, N. Yu, Y. Yang, *Nano Lett.* **2021**, 21, 1412.

[4] A. P. Raman, M. A. Anoma, L. Zhu, E. Rephaeli, S. Fan, *Nature* **2014**, 515, 540.

[5]

[6] Y. Fu, Y. D. An, Y. K. Xu, J. G. Dai, D. Y. Lei, *EcoMat* **2022**, 4, e12169, e12169.

[7] R. A. Yalçın, H. Ertürk, *Mater. Res. Express* **2019**, 6, 085551.

[8] X. Li, J. Peoples, Z. Huang, Z. Zhao, J. Qiu, X. Ruan, *Cell Rep. Phys. Sci.* **2020**, 1, 100221.

[9] L. Quan, Z. Changfang, R. Nirmala, *J. Biomed. Opt.* **2003**, 8, 223.

[10] M. Neuman, S. Edvardsson, P. Edström, *Opt. Lett.* **2015**, 40, 4325.

[11] Z. Liu, S. Liu, K. Wang, X. Luo, *Appl. Opt.* **2010**, 49, 247.

[12] S. Azam, M. Irfan, S. A. Khan, Z. Ali, I. V. Kityk, S. Muhammad, A. G. Al-Sehemi, *J. Alloys Compd.* **2019**, 771, 1072.

[13] M. Kučera, P. Hasa, J. Hakenová, *J. Alloys Compd.* **2008**, 451, 146.

[14] A. Solodovnyk, D. Riedel, B. Lipovšek, A. Osvet, J. Gast, E. Stern, K. Forberich, M. Batentschuk, J. Krč, M. Topič, C. J. Brabec, *Opt. Mater. Express* **2017**, 7, 2943.

[15] J. Xu, R. Wan, W. Xu, Z. Ma, X. Cheng, R. Yang, X. Yin, *Mater. Today Nano* **2022**, 19, 100239.

[16] K. X. Lin, Y. W. Du, S. R. Chen, L. K. Chao, H. H. Lee, T. C. Ho, Y. H. Zhu, Y. J. Zeng, A. Q. Pan, C. Y. Tso, *Energy Build.* **2022**, 276, 112507, 112507.

[17] C.-L. Luo, L.-X. Zheng, J.-Y. Jiao, W.-G. Yan, J. Zhao, G.-Z. Jia, Z.-F. Liu, Z.-B. Liu, J.-G. Tian, *Opt. Mater.* **2023**, 138, 113710.

[18] W. Wu, Z. Zhang, R. Dong, G. Xie, J. Zhou, K. Wu, H. Zhang, Q. Cai, B. Lei, *J. Rare Earths* **2020**, 38, 539.

[19] R. Cao, X. Wang, X. Ouyang, Y. Jiao, Y. Li, H. Wan, W. Li, Z. Luo, *J. Lumin.* **2020**, 224, 117292.

[20] K. A. Denault, J. Brgoch, M. W. Gaultois, A. Mikhailovsky, R. Petry, H. Winkler, S. P. DenBaars, R. Seshadri, *Chem. Mater.* **2014**, 26, 2275.

[21] H. Yang, Y.-S. Kim, *J. Lumin.* **2008**, 128, 1570.

[22] V. Bachmann, C. Ronda, A. Meijerink, *Chem. Mater.* **2009**, 21, 2077.

[23] E. K. Tekgül, Ş. Yaltkaya, *J. Lumin.* **2024**, 120517.

[24] J. Kim, Y. J. Kim, *J. Korean Ceram. Soc.* **2020**, 57, 85.

[25] F. Chen, Y. Huang, R. Li, S. Zhang, Q. Jiang, Y. Luo, B. Wang, W. Zhang, X. Wu, F. Wang, P. Lyu, S. Zhao, W. Xu, F. Wei, R. Zhang, *Sci. Adv.*, 8, eabn5882.

[26] X. Wu, J. Li, Q. Jiang, W. Zhang, B. Wang, R. Li, S. Zhao, F. Wang, Y. Huang, P. Lyu, Y. Zhao, J. Zhu, R. Zhang, *Nat. Sustain.* **2023**, 6, 1446.

[27] J. Li, Y. Fu, J. Zhou, K. Yao, X. Ma, S. Gao, Z. Wang, J.-G. Dai, D. Lei, X. Yu, *Sci. Adv.* **2023**, 9, eadg1837.

[28] R. Levinson, S. Chen, C. Ferrari, P. Berdahl, J. Slack, *Energy Build.* **2017**, 152, 752.

[29] Z. Tong, J. Peoples, X. Li, X. Yang, H. Bao, X. Ruan, *arXiv preprint arXiv:2101.05053* **2021**.

[30] C. Ziming, L. Bo, S. Xuhang, W. Fuqiang, L. Huaxu, S. Yong, *AIMS Energy* **2021**, 9, 96.

[31] W. Li, Y. Shi, Z. Chen, S. Fan, *Nat. Commun.* **2018**, 9, 4240.

[32] G. J. Lee, Y. J. Kim, H. M. Kim, Y. J. Yoo, Y. M. Song, *Adv. Opt. Mater.* **2018**, 6, 1800707.

[33] J. W. Cho, E. J. Lee, S. K. Kim, *Nano Lett.* **2022**, 22, 380.

[34] W. Zhu, B. Droguet, Q. Shen, Y. Zhang, T. Parton, X. Shan, R. Parker, M. Volder, T. Deng, S. Vignolini, T. Li, *Adv. Sci.* **2022**, 9, 2202061.

[35] S. X. Yu, Q. Zhang, Y. F. Wang, Y. W. Lv, R. J. Ma, *Nano Lett.* **2022**, 22, 4925.

[36] Y. J. Chen, J. Mandal, W. X. Li, A. Smith-Washington, C. C. Tsai, W. L. Huang, S. Shrestha, N. F. Yu, R. P. S. Han, A. Cao, Y. Yang, *Sci. Adv.* **2020**, 6, eaaz5413.

[37] K. Lin, S. Chen, Y. Zeng, T. C. Ho, Y. Zhu, X. Wang, F. Liu, B. Huang, C. Y.-H. Chao, Z. Wang, C. Y. Tso, *Science* **2023**, 382, 691.

[38] X. Wang, Q. Zhang, S. Wang, C. Jin, B. Zhu, Y. Su, X. Dong, J. Liang, Z. Lu, L. Zhou, W. Li, S. Zhu, J. Zhu, *Sci. Bull.* **2022**, 67, 1874.

[39] S. Son, S. Jeon, D. Chae, S. Y. Lee, Y. Liu, H. Lim, S. J. Oh, H. Lee, *Nano Energy* **2021**, 79, 105461.

[40] X. Ma, Y. Fu, D. Liu, N. Yang, J. G. Dai, D. Lei, *Adv. Opt. Mater.* **2024**, 12, 2303296.

[41] X. Ma, Y. Fu, N. Yang, X. Hu, J.-G. Dai, B. Fei, D. Lei, *J. Mater. Chem. A* **2024**, 12, 20921.

[42] X. Hou, K. Zhang, X. Lai, L. Hu, F. Vogelbacher, Y. Song, L. Jiang, M. Li, *Matter* **2025**, 8, 101898.

[43] J. Liu, B. Ji, Y. Zhong, L. Zhang, B. Wang, X. Feng, H. Xu, Z. Mao, *ACS Nano* **2025**, 19, 10263.

[44] Y. Zhou, C. Lu, R. Xiong, *ACS Nano* **2025**, 19, 5029.
